# A revision of *Spondias* L. (Anacardiaceae) in the Neotropics

**DOI:** 10.3897/phytokeys.55.8489

**Published:** 2015-08-05

**Authors:** John D. Mitchell, Douglas C. Daly

**Affiliations:** 1 Institute of Systematic Botany, The New York Botanical Garden, 2900 Southern Blvd., Bronx, NY 10458-5126

**Keywords:** Anacardiaceae, fruit trees, leaf architecture, Neotropics, new species, *Spondias*, taxonomy, tropical crops

## Abstract

As part of an ongoing study of Anacardiaceae subfamily Spondioideae, the ten native and one introduced species of *Spondias* in the Neotropics are revised. The genus is circumscribed. Three new species, *Spondias admirabilis*, *Spondias expeditionaria*, and *Spondias globosa*, are described and illustrated; a key to the taxa found in the Neotropics and distribution maps are provided. The Paleotropical species and allied genera are reviewed. Diagnostic character sets include leaf architecture, habit, flower morphology, and gross fruit morphology. Notes on the ecology and economic botany of the species are provided.

## Introduction

*Spondias* L. is a genus of fruit trees that comprises 18 species native to tropical America and Asia, and Madagascar. It is the type genus of the subfamily Spondioideae Takht. emend. Pell & J. D. Mitch., which is indicated by molecular systematic work currently under way as being the more basal (but possibly polyphyletic) group of a monophyletic Anacardiaceae sister to the Burseraceae (Pell 2004; Mitchell et al. 2006; Pell et al. 2011). This corroborates previous conclusions by Wannan and Quinn (e.g., 1990), who noted that the endocarp structure of Spondioideae is similar to that of *Canarium* (Burseraceae), by Bachelier and Endress (2009), who noted that those floral morphology and anatomy characters shared by Burseraceae and Anacardiaceae are usually found in the Spondioideae (as Spondiadoideae), and by Terrazas (1994), whose results from cladistic analyses of wood and leaf anatomical characters and rbcL sequences also showed the group as basal.

The subfamily is characterized by consistently obdiplostemonous flowers (*sensu* Bachelier and Endress 2009) and by usually separate styles, 4–5-carpellate ovaries, apical ovule insertion, thick exocarp, and a usually bony endocarp composed of a mass of strongly lignified and irregularly oriented sclerenchyma, often but not always operculate.

## Taxonomic history

Generic limits and definitions in the Anacardiaceae have been re-drawn several times during the past century and still need some work; revision of *Spondias* has required further re-examination of subfamily Spondioideae and led to the discovery of at least one new genus from Madagascar (Mitchell et al. 2006) and a few possible additional segregate genera.

*Spondias* was one of the first genera of Anacardiaceae described by Linnaeus (1737: 365), with the type species *Spondias mombin* published in 1753. Inexplicably, he later published two names homotypic with *Spondias mombin* (and therefore illegitimate), *Spondias myrobalanus* (Linnaeus 1759a: 1036) and *Spondias lutea* (Linnaeus 1762: 613). Furthermore, he published the name *Spondias myrobalanus* a second time, in *Flora jamaicensis* (Linnaeus 1759b: 16), in this instance an illegitimate homonym homotypic with *Spondias purpurea*. The syntypes for the latter *Spondias myrobalanus* served as the basis for *Spondias purpurea*, for which Bornstein (1989) selected a lectotype. Despite its early establishment and economic importance, numerous taxa have been assigned to *Spondias* that are no longer accepted by most authors. This fact is illustrated by Kostermans (1991) who in his treament of Asian *Spondias*, referred the excluded species to 15 different genera, three of these not even in the Spondioideae as currently defined.

Bentham and Hooker (1862) were the first to formulate groupings of genera in the Anacardiaceae, dividing the family into two tribes, the Anacardieae and Spondieae [sic]. Subsequently, Marchand (1869) published the tribe Spondiadeae (as Spondieae) and was the first to formulate a relatively modern concept of *Spondias*, in which he included *Evia* Blume, *Cytheraea* Wight & Arn., and *Wirtgenia* Jung. ex Hassk. (nom. illegit., non *Wirtgenia* Sch. Bip.). On the other hand, of the taxa he either accepted in *Spondias* or recognized as synonyms of species in the genus, four are considered here to belong to other genera (see excluded species section).

Two treatments of Asian Spondioideae took opposite extremes in the circumscription of the genus. In their revision of tropical Asian *Spondias*, Airy Shaw and Forman (1967) lumped *Allospondias* and *Solenocarpus* with a rather broadly defined *Spondias*, but this would leave *Spondias* without a single synapomorphy and in fact joins taxa with disparate character states: simple vs. bipinnately compound leaves, unicarpellate vs. multicarpellate ovaries, and leaflets with or without an intramarginal vein. Ding Hou’s (1978) treatment of the family for *Flora Malesiana* followed Airy Shaw and Forman’s broad circumscription.

In contrast, Kostermans (1981, 1991) defined the genera of the Spondioideae rather narrowly, maintaining *Allospondias* and *Solenocarpus*, transferring *Spondias philippinensis* (Elmer) Airy Shaw and Forman to the latter, describing the new genus *Haplospondias*, and formally returning the South Pacific species *Spondias dulcis* Parkinson into the pre-existing genus *Evia* Commerson ex Blume *emend*. Kosterm. Pending completion of a molecular systematic-based generic revision of the subfamily, we comment briefly on these in turn:

We agree with Kostermans that *Allospondias lakonensis* (Pierre) Stapf [syn.: *Spondias lakonensis* Pierre] should be kept out of *Spondias*, on the basis of the former’s lack of an intramarginal vein and presence of perpendicular epimedial tertiary veins, highly branched sclereids terminating the FEVs (freely ending leaf veinlets), styles connivent at anthesis and stigmas extrorse on the developing fruit, lack of a fibrous matrix on the endocarp, and presence of parenchyma-filled chambers in the endocarp.

Kostermans’ circumscription of *Allospondias* encompassed *Spondias laxiflora* (Kurz) Airy Shaw & Forman; we concur that it does not belong in *Spondias* because of its eucamptodromous secondary venation (vs. intramarginal vein), versatile (vs. fixed) anthers, and endocarp apertures not covered by the fibrous matrix, but we reserve judgment on its placement in *Allospondias*: while *Allospondias laxiflora* and *Allospondias lakonensis* (the type species of *Allospondias*) share an apert calyx, valvate corolla, and papillate stigmas, and both lack a fibrous matrix outside the endocarp, *Allospondias lakonensis* has the styles continuous with the lateral lobes on the pistil and connivent or appearing connate apically, the stigmas spathulate, while *Allospondias laxiflora* has the styles free and not continuous with the lateral lobes on the pistil, the stigmas capitate or discoid. Moreover, the fruit in *Allospondias lakonensis* is radially symmetrical and 4–5-lobed and has parenchyma-filled chambers, while in *Allospondias laxiflora* it is strongly oblique and unlobed and lacks parenchyma-filled chambers.

We agree provisionally with Kostermans that *Solenocarpus indicus* Wight & Arn. does not belong in *Spondias*. While like *Spondias* it has admedial tertiary veins originating near the margin, an intramarginal vein, apert calyx (imbricate in *Spondias purpurea*), and valvate corolla (quincuncial in *Spondias purpurea*), in contrast to *Spondias* s.s. it has a single flabellate style, single stigma and unicarpellate gynoecium, and it lacks a fibrous matrix on the endocarp. Moreover, in *Solenocarpus* the sclereids in the mesocarp occur only in a layer just outside the endocarp, while in *Spondias* they essentially sheath the resin canals, which are scattered throughout the mesocarp (Wannan and Quinn 1990).

We agree with Kostermans that *Spondias philippinensis* should be removed from *Spondias* on the basis of its eucamptodromous secondary venation (vs. intramarginal vein), single narrowly flabellate style, single stigma, unicarpellate ovary, and strongly oblique fruit, and we tentatively agree with Kostermans’ placement of this species in *Solenocarpus*, because although *Spondias philippinensis* lacks an intramarginal vein, its flower morphology is very similar to that of *Solenocarpus indicus*: apert calyx, valvate corolla, single narrowly flabellate style, and unicarpellate gynoecium.

We agree that monotypic *Haplospondias brandisiana* is distinct from *Spondias*; it has simple leaves without an intramarginal vein, and a single style with an oblique (and possibly bilobed) stigma.

Kostermans (1991) published *Spondias tonkinensis* Kost. and later considered it to be a taxonomic synonym of *Spondias petelotii* (Tardieu-Blot) Kost. (syn.: *Dracontomelon petelotii* Tardieu-Blot; Kostermans 1992), but our generic assignment of this taxon is as yet uncertain. It has festooned-brochidodromous leaflet venation with raised-marsupiform (pouch-like) domatia in the axils of the secondary veins, calyx lobes open in bud but their bases imbricate, petals imbricate, anther connective glandular (as in *Cyrtocarpa*), styles connivent at anthesis, and stigmas slightly extrorse and slightly ellipsoid. Each of these character states can be found elsewhere in the Spondioideae but by no means in this combination.

Placement of *Spondias dulcis* in *Evia* was based on the endocarp with spinose projections penetrating the mesocarp (vs. a simple fibrous matrix) and the woody pedicel partially embedded in the basicrescent developing fruit. In light of the fact that *Spondias dulcis* shares similar flower morphology as well as the intramarginal vein and other aspects of leaf architecture with the American species of *Spondias*, these fruit characters could be seen as derived within *Spondias*, rather than warranting recognition at generic rank.

Placement of *Spondias bipinnata* presents a challenge. Like *Spondias*, it has an apert calyx, valvate corolla, the endocarp with a fibrous matrix, and a 5-locular stone. Unlike *Spondias*, it has bipinnate leaves, lacks an intramarginal vein, and has pubescent styles; other aspects of its morphology are shared with *Allospondias lakonensis*, including virtually identical leaflet venation and connivent styles.

More recently, the genus *Attilaea* was described from the Yucatán of Mexico by Martínez and Ramos-Álvarez (2007. *Attilaea* is similar to *Spondias* because of its intramarginal vein and red flowers (like in *Spondias purpurea*), but it differs by the climbing habit, the 2-carpellate, unilocular gynoecium (vs. (3–4)5-carpellate, 4-5-locular), and the single seed per fruit (vs. 4–5).

At species rank, Marchand (1869) placed *Spondias mombin* as a synonym of the nomen illegitimum *Spondias lutea* L.; this error was not corrected until 1926 by Fawcett and Rendle. In addition, he recognized the illegitimate *Spondias dulcis* G. Forst. (a synonym of *Spondias dulcis* Parkinson), and described var. amara, including in it several taxonomic entities referred by Kostermans (1991) and the present authors to *Spondias acuminata*, *Spondias pinnata*, and *Spondias malayana*.

In *Flora brasiliensis*, Engler (1876) kept *Spondias mombin* as a synonym of the nomen illegitimum *Spondias lutea*, put *Spondias venulosa* as a variety of *Spondias purpurea*, and described *Spondias macrocarpa*.

In *Monographie phanerogamerum*, Engler (1883) placed *Warmingia pauciflora* Engl. in synonymy with *Spondias purpurea* (which he had placed in Burseraceae in 1876). He also placed *Spondias macrocarpa* as well as the Asian taxon *Spondias acida* as varieties of *Spondias dulcis*. He raised Spondias purpurea var. venulosa to specific rank, and recognized *Spondias mangifera*, a name considered by Kostermans (1991) as illegitimate under *Spondias pinnata*.

In the *Flora of Jamaica*, Fawcett and Rendle (1926) were perhaps the first to critically examine the early literature and place *Spondias lutea* in synonymy with *Spondias monbin* [sic], but they erroneously placed the Eastern Brazilian *Spondias macrocarpa* in synonymy with *Spondias cytherea* (=*Spondias dulcis*). They illustrated both staminate and pistillate flowers of *Spondias purpurea*.

In the *Flora of the Lesser Antilles*, Bornstein (1989) lectotypified *Spondias mombin* and *Spondias purpurea*; he also researched the typification of *Spondias lutea* and *Spondias myrobalanus* (Syst. Nat. 10, 2: 1036. 1759.) and explained why these are both nomina illegitima, being based on the same types as *Spondias mombin*. He also pointed out that *Spondias purpurea* is a nomen novum for the illegitimate homonyn for *Spondias myrobalanus* L. (Fl. jamaic. 16. 1759).

In the *Flora of Panama*, Blackwell and Dodson (1967 [1968]) considered *Spondias radlkoferi* to be synonymous with *Spondias mombin*, but later Croat (1974a, b) revived the species, based on characters of leaflet venation, indumentum, bark and slash, leaf phenology, and flower and fruit phenology and morphology. Blackwell and Dodson also mistakenly placed *Spondias cytherea* (= *Spondias dulcis*), *Spondias macrocarpa*, and Spondias purpurea var. venulosa (= *Spondias venulosa*) in synonymy with *Spondias purpurea*. Inexplicably, in the *Flora of Ecuador*, Barfod (1987) sank *Spondias radlkoferi* in synonymy with *Spondias mombin*. Finally, the present authors described *Spondias testudinis* from Southwestern Amazonia (Mitchell and Daly 1998) and *Spondias tefyi* from Madagascar (Mitchell et al. 2012).

The eighteen species of *Spondias* accepted in the present treatment, their geographic distributions, and their principal synonyms are summarized in Table 1.

**Table 1.** The taxa of *Spondias* accepted in the present treatment (taxa revised herein are shaded).

Taxon	Distribution	Principal synonyms
*Spondias acida* Blume	W. Malesia	Spondias dulcis Parkinson var. acida (Blume) Marchand, *Evia acida* (Blume) Blume
*Spondias acuminata* Roxb.	India, Myanmar (Burma), Thailand (not Malesia)	Treated as synonym of *Spondias pinnata* in *Flora of China* (Min & Barfod, 2008)
*Spondias admirabilis* J. D. Mitch. & Daly	Atlantic Forest of Rio de Janeiro, Brazil	
*Spondias dulcis* Parkinson	Pacific; widely cultivated in tropics	*Evia dulcis* (Parkinson) Blume, *Spondias cytherea* Sonn.
*Spondias expeditionaria* J. D. Mitch. & Daly	Atlantic Forest of Espírito Santo and Minas Gerais, Brazil	
*Spondias globosa* J. D. Mitch. & Daly	W Amazonia; outlier in Zulia, Venezuela	
*Spondias macrocarpa* Engl.	E Brazil	Spondias dulcis Parkinson var. macrocarpa (Engl.) Engl.
*Spondias malayana* Kosterm.	Malesia	
*Spondias mombin* L.	Mexico to Bolivia and E Brazil; widely cultivated in moist tropics	*Spondias lutea* L.
*Spondias novoguineensis* Kosterm.	Malesia E of Sulawesi	
*Spondias pinnata* (Koenig ex L.f.) Kurz	Indian subcontinent, Indochina, S China	*Mangifera pinnata* Koenig ex L.f., *Spondias mangifera* Willd., *Spondias amara* Lam., *Spondias bivenomarginalis* Feng K. M., P. Y. Mao, & P. Y. Mao
*Spondias purpurea* L.	NW Mexico to Panama (possibly N Colombia and SW Ecuador; widely cultivated in tropics	*Spondias cirouella* Tussac, *Spondias jocote-amarillo* Kosterm., *Spondias mexicana* Watson, *Spondias negrosensis* Kosterm.
*Spondias radlkoferi* Donn.-Sm.	Mexico to C America to NW Venezuela and Colombia	*Spondias nigrescens* Pittier
*Spondias tefyi* J. D. Mitch., Daly, & Randrian.	Madagascar	
*Spondias testudinis* J. D. Mitch. & Daly	SW Amazonia	
*Spondias tuberosa* Arruda	NE Brazil; cultivated elsewhere in Brazil	
*Spondias venulosa* (Mart. ex Engl.) Engl.	E Brazil	Spondias purpurea L. var. venulosa Mart. ex Engl.
*Spondias xerophila* Kosterm.	Sri Lanka	[Kostermans (1991): this may be a syn. of *Spondias acuminata*]

### Comments on phylogeny

It is exceedingly difficult to purify and amplify DNA even from fresh leaf samples of *Spondias* (S. Pell and A. Miller, pers. comm.); to date, sequences have been obtained from *Spondias globosa*, *Spondias mombin*, *Spondias purpurea*, *Spondias testudinis*, and *Spondias tuberosa*, but not from any taxa native to the Paleotropics other than *Spondias dulcis*, *Spondias malayana* and *Spondias pinnata*. Current data suggest that the subfamily Spondioideae is divided into two clades; the smaller and more basal of these contains *Spondias* along with *Allospondias*, *Dracontomelon*, *Pegia*, and *Pseudospondias*. Neotropical *Spondias* is sister to Asian *Spondias* (Weeks et al. 2014).

### Wood anatomy

The wood anatomy of several *Spondias* species has been described and compared with other Anacardiaceae genera. The following characterization of *Spondias* wood anatomy is based on the published work of Dadswell and Ingle (1948, *Spondias dulcis* and *Spondias novoguineensis*, the latter cited as *Spondias pinnata* in the text), Kryn (1952, *Spondias acida*, *Spondias dulcis*, *Spondias pinnata*), Deschamps (1979, *Spondias mombin*), Paula and Alves (1980, *Spondias tuberosa*), Barajas-Morales and Gómez (1989, *Spondias purpurea*), Terrazas (1994, *Spondias dulcis*, *Spondias malayana*, *Spondias mombin*, *Spondias pinnata*, *Spondias purpurea*, *Spondias radlkoferi* and allied genera), Ter Welle et al. (1997, *Spondias mombin*), Terrazas 1999, Gómez (2009; *Spondias purpurea*). The differences among species are subtle, and they mostly overlap in both quantitative and qualitative aspects. The only native Neotropical species whose wood anatomy has been studied are *Spondias mombin*, *Spondias purpurea*, *Spondias radlkoferi*, and *Spondias tuberosa*.

*Vessels* diffuse-porous to rarely slightly semi-ring-porous with a vessel density ranging from 2–14/mm^2^ (visible without a hand lens), and varying from 121–357 µm diam, mostly solitary or in groups of 2–8, round to slightly oval in outline. *Parenchyma* paratracheal, vasicentric, sometimes aliform. *Rays* heterogeneous (homogeneous in *Spondias tuberosa*, Paula and Alves 1980), Kribs Type II or III, rhombic crystals of calcium oxalate often present. *Fibers* mostly libriform and septate. Axial and radial gum ducts and resin canals present. Terrazas (1999) observed that the distinguishing characteristic of *Spondias* wood is the large diameter of the vessels (often >150 µm) relative to other Anacardiaceae genera.

The wood of the recently described Anacardiaceae genus *Attilaea* (Martínez and Ramos-Álvarez, 2007), which is endemic to the Yucatán Peninsula, has been compared with that of the sympatric and morphologically similar *Spondias purpurea* (Gómez 2009, Table 2, p. 81; see discussion above), and reportedly there are some significant differences that separate them: higher average vessel density of 22/mm^2^ (vs. 9.7/mm^2^ in *Spondias purpurea*); smaller vessel diameter (mean 115.6 µm diam vs. mean199 µm diam in *Spondias purpurea*); and rays heterogeneous Kribs type II and biseriate (vs. heterogenous Kribs Type II and III and triseriate in *Spondias purpurea*).

### Leaf architecture

**Figure 1.**
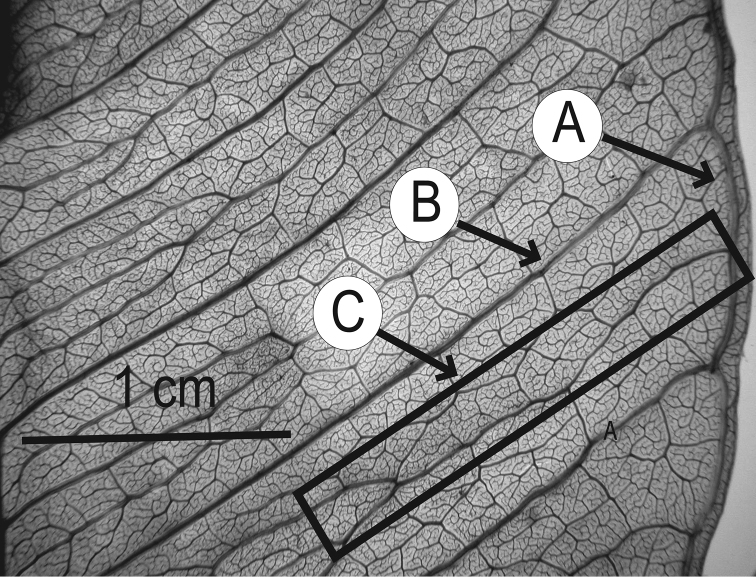
Leaf architecture of *Spondias globosa*. **A** Intramarginal vein **B** Secondary vein course straight **C** Composite admedial tertiary vein branching from intramarginal (defined by box)(from *Vásquez 12866*, NY).

**Figure 2.**
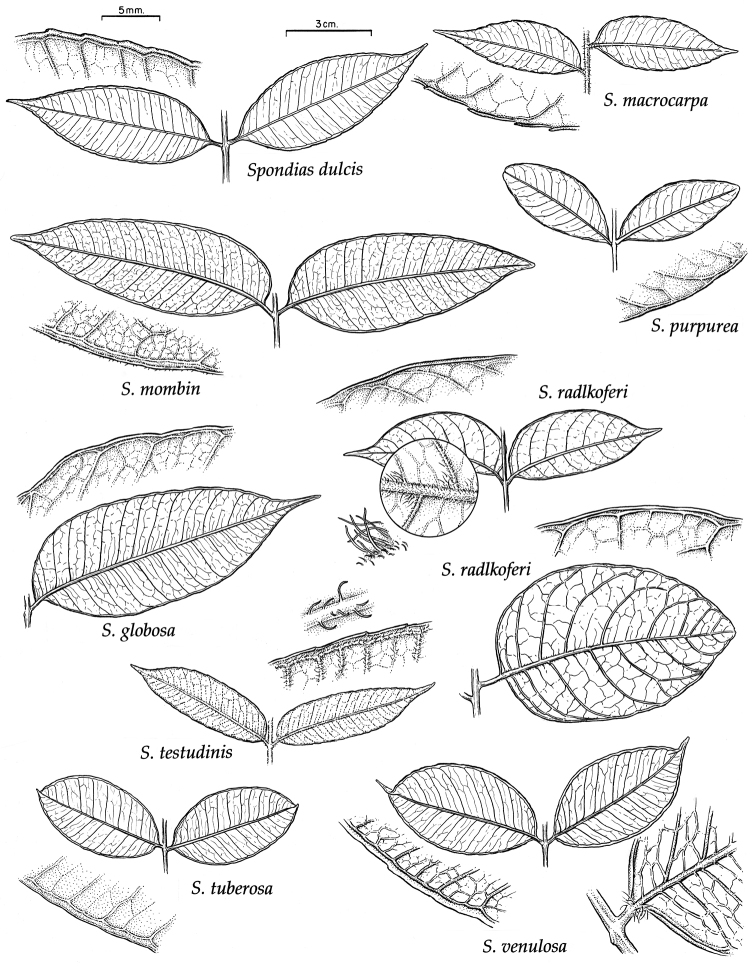
Leaflets of *Spondias* species, showing details of the margin (insets): *Spondias dulcis* (*Ayala & Criollo 3982*, NY); *Spondias macrocarpa* (*Thomas et al. 6823*, NY); *Spondias mombin* (*Acevedo 6037*, NY); *Spondias globosa* (*Neill & Palacios 7079*, NY); *Spondias radlkoferi* narrow-leaflet form (*Crane 458*, LL) and broad-leaflet form (*Contreras 6976*, LL); *Spondias purpurea* (*Grijalva 770*, NY); *Spondias testudinis* (Lao Magín 112, NY); *Spondias tuberosa* (*Carvalho et al. 3767*, NY); *Spondias venulosa* (*Stehmann 20855*, NY).

**Figure 3.**
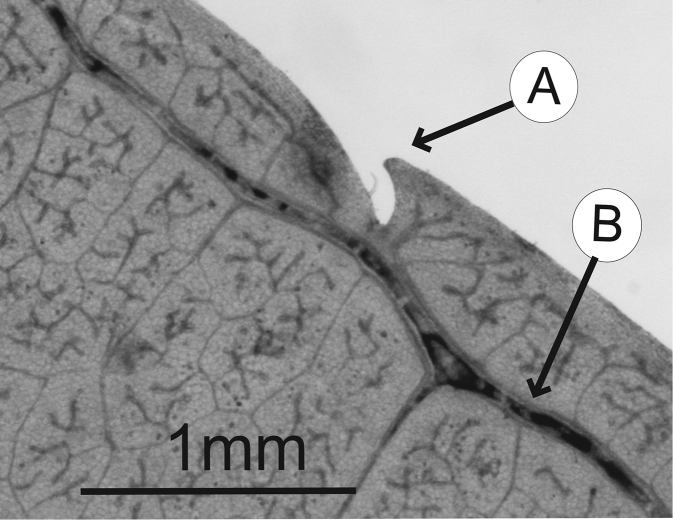
Leaf architecture of *Spondias purpurea*. **A** Concave-convex tooth **B** Intramarginal vein (from *Madsen 63428*, NY).

**Figure 4.**
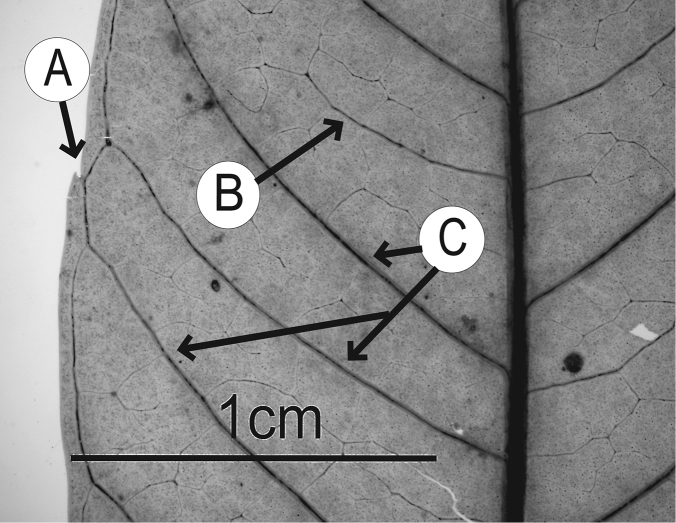
Leaf architecture of *Spondias purpurea* (contd.). **A** Tooth **B** Intersecondary vein **C** Irregularly spaced, arcuate secondaries. (from *Madsen 63428*, NY).

**Figure 5.**
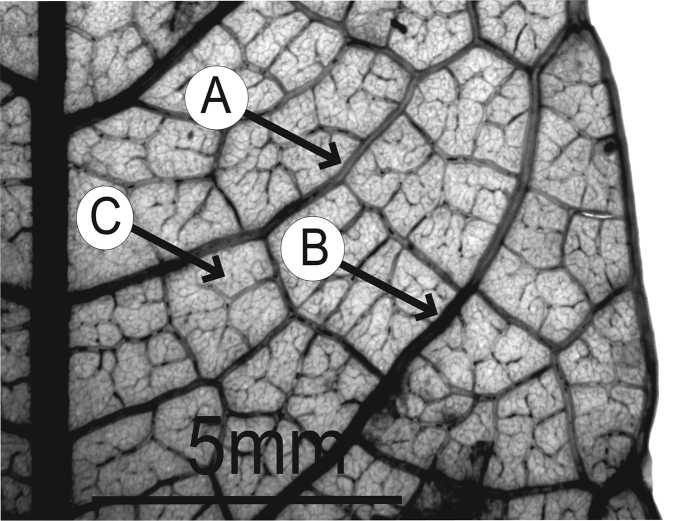
Leaf architecture of *Spondias radlkoferi*. **A** Intersecondary vein **B** Arcuate secondary **C** Area of highly branched sclereids (from *Mitchell 120*, NY).

**Figure 6.**
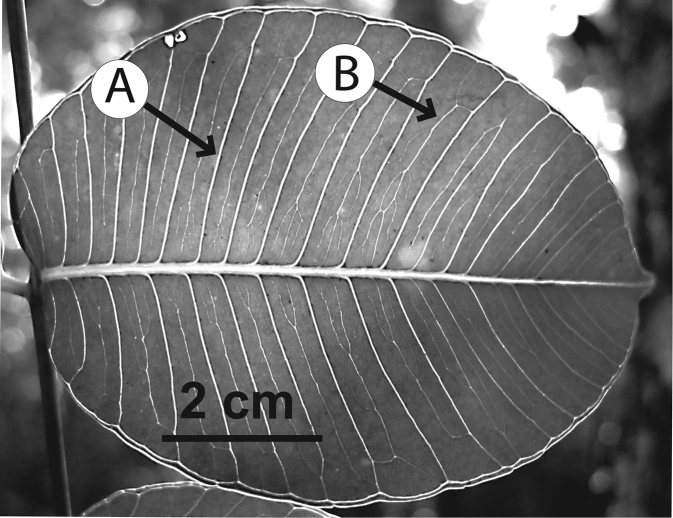
Leaf architecture of *Spondias venulosa*. **A** Straight secondary vein course **B** Admedial tertiary vein (from *Stefano et al. 201*, NY).

**Figure 7.**
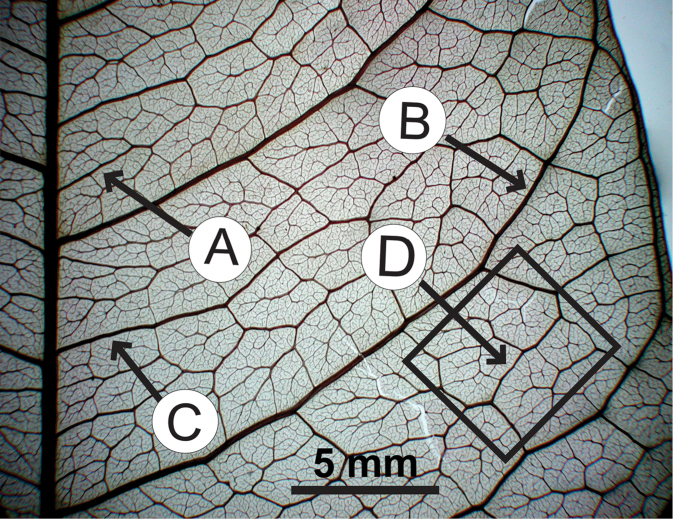
Leaf architecture of *Spondias mombin*. **A** Epimedial tertiaries **B** Somewhat arcuate secondary vein course **C** Intersecondary vein **D** Area of irregular-reticulate tertiaries. (from *Hopkins 1454*, NY).

**Figure 8.**
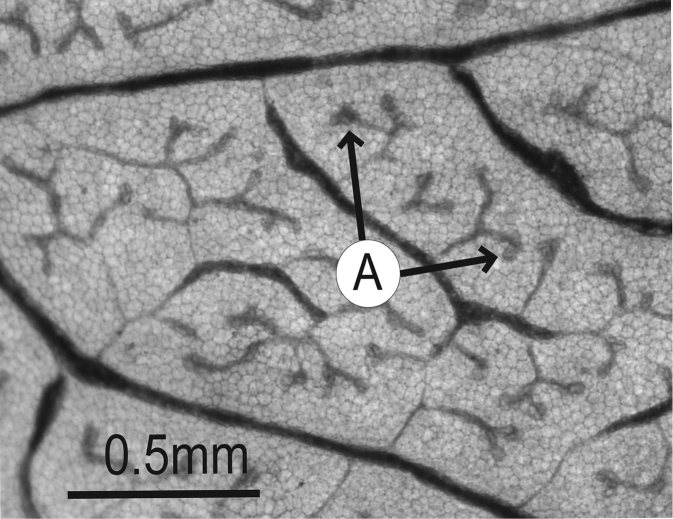
Leaf architecture of *Spondias admirabilis*. **A** FEVs terminating in tracheoid idioblasts (from *Farney et al. 3957*, NY).

**Figure 9.**
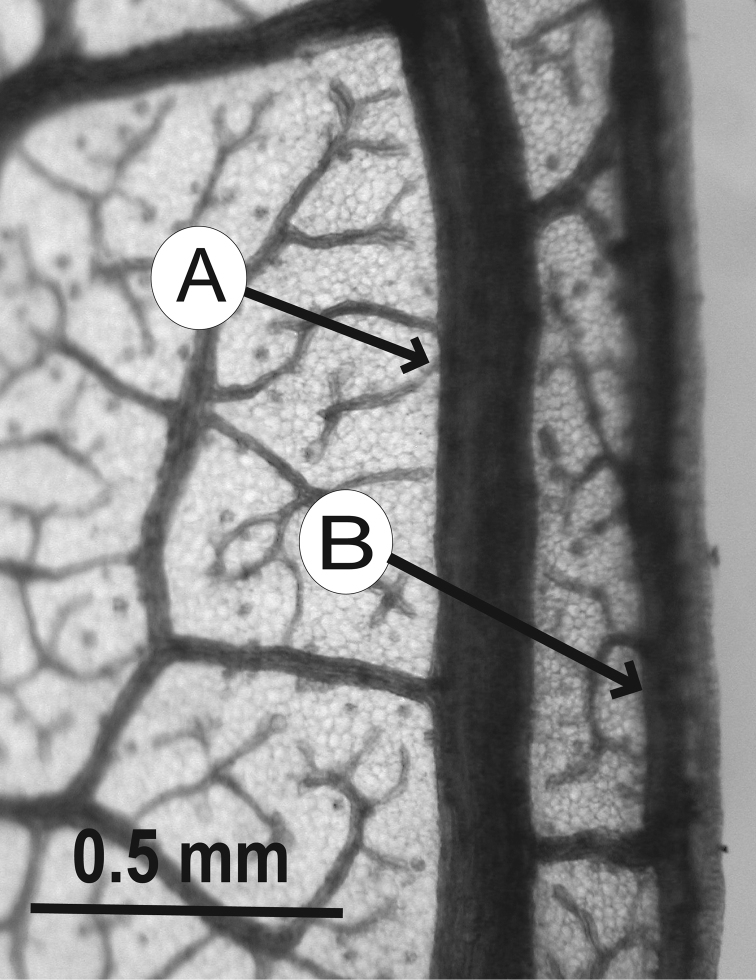
Leaf architecture of *Spondias mombin* (contd.). **A** Intramarginal vein **B** Fimbrial vein. (from *Hopkins 1454*, NY).

**Figure 10.**
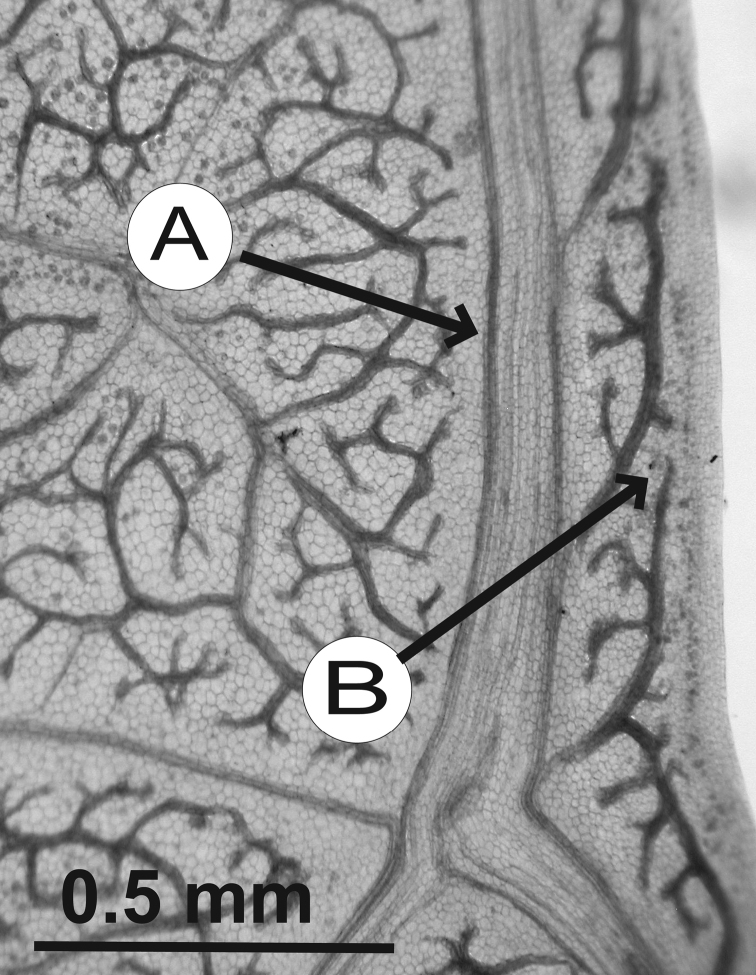
Leaf architecture of *Spondias globosa* (contd.). **A** Intramarginal vein **B** Incompletely looped marginal ultimate venation. (from *Nelson 786*, NY).

**Figure 11.**
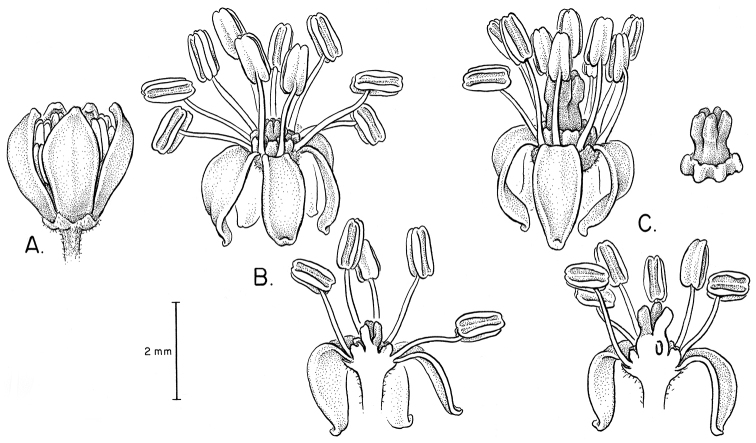
Flowers of *Spondias mombin*. **A** Opening bud **B** Flower at anthesis (top) and longisection at center of same **C** Flower post-anthesis with pistil further developed (top), longisection at center of same (bottom), and disk and pistil. **A–C** from *Rusby & Squires 102* (NY).

**Figure 12.**
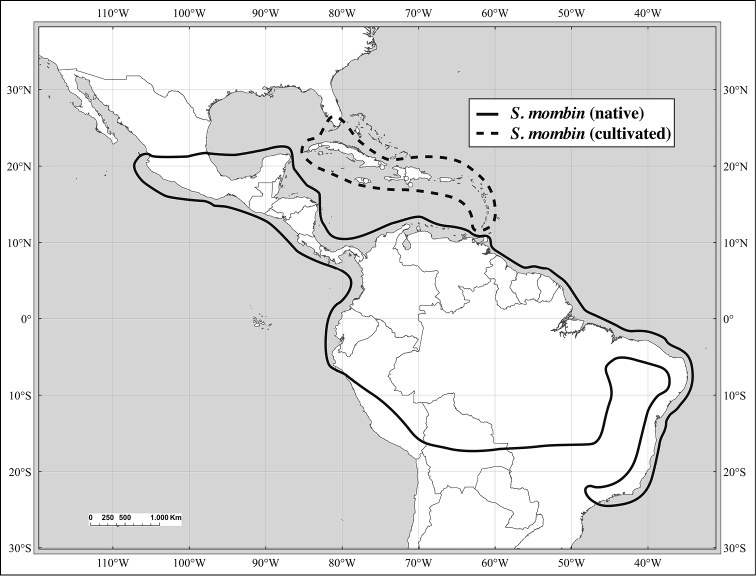
Distribution of *Spondias mombin*.

The present work uses the terminology in Ellis et al. (2009), and the genus and species descriptions contain details on leaf architecture. Although the presence of an intramarginal vein (Fig. 1) on leaflets is characteristic of *Spondias* (except present in *Attilaea abalak* and *Solenocarpus indicus*), the genus presents a wealth of other characters of its leaf architecture that are variable and taxonomically useful (see below the key to the Neotropical species based on leaf(let) characters). Many macromorphological leaf characters and virtually every major character of the leaflet venation and symmetry shows at least some variation among species; the following characters have proven most useful: (1) number of leaflet pairs; (2) leaflet shape; (3) leaflet margin rolling and dentition, when present (Figs 2–4); (4) secondary vein angle and spacing (e.g., irregular spacing in *Spondias purpurea*, Fig. 3); (5) secondary vein course (straight in Figs 1, 6; arcuate in Figs 4, 5, 7); (6) presence of intersecondaries (present in *Spondias purpurea*, Fig. 4 and *Spondias radlkoferi*, Fig. 5); (7) presence of admedial tertiaries (Figs 1, 5) or irregular-reticulate tertiaries (Figs 5, 7); (8) basal symmetry; (9) presence of epimedial tertiaries (present in *Spondias mombin*, Fig. 7); (10) presence of highly branched sclereids (present in *Spondias radlkoferi*, Fig. 5); (11) presence of tracheoid idioblasts (present in *Spondias admirabilis*, Fig. 8); (12) presence of a fimbrial vein (Fig. 9); and (13) looping of the marginal ultimate veins (absent, complete, or incomplete; e.g., incompletely looped in *Spondias globosa*, Fig. 10).

### Breeding systems, floral morphology, flower and fruit anatomy

Flowers of all species except *Spondias purpurea* are morphologically hermaphroditic (Bachelier and Endress 2009); *Spondias purpurea* is dioecious and the flowers display relatively strong sexual dimorphism for the duration of the flower: the pistillate flowers have staminodes where microspore mother cells degenerate during meiosis, and in a tropical dry forest in Morelos, Mexico the staminate flowers develop a month earlier than the pistillate ones (Hernández-Martínez et al. 1999). In the other species of *Spondias* occurring in the Neotropics, we have observed strong protandry, such that on a given inflorescence one finds flowers with dehiscing anthers and an underdeveloped gynoecium at early anthesis, and flowers with fully-developed pistil and passed anthers at late anthesis (see Fig. 11); it is likely that most if not all reports of staminate flowers are in fact simply hermaphrodite flowers in early anthesis. In *Spondias mombin*, Lozano (1986) reported hermaphroditic, staminate, and pistillate flowers on the same plant; she determined that in some flowers the anthers produced sterile pollen, and she interpreted the poorly developed pistil in some flowers as representing staminate flowers with suppressed gynoecia. Nadia et al. (2007) concluded that *Spondias tuberosa* is andromonoecious; Bawa (1974) concluded that *Spondias mombin* is monoecious and strongly self-incompatible while Stacy et al. (1996) found that species to have functionally hermaphroditic flowers, and Croat (1974a) reported *Spondias radlkoferi* to have mostly bisexual or rarely pistillate flowers.

Floral morphology and anatomy of *Spondias* have been studied by Lozano (1986b; *Spondias mombin*), Bachelier and Endress (2009; *Spondias dulcis* and *Spondias purpurea*), and Hernández-Martínez et al. (1999; *Spondias purpurea*). The flowers of *Spondias* are pentamerous and isomerous. The sepals are free although early aestivation is quincuncial. Corolla aestivation is valvate, and at anthesis the petals are free and reflexed or patent ((sub)erect only in *Spondias purpurea*). The flowers are obdiplostemonous (the outer whorl of stamens opposite the carpels); the antepetalous stamens are shorter at all stages. The anthers are dorsifixed on the lower half in *Spondias dulcis* but dorsifixed at the base in *Spondias purpurea*; all species are longitudinally dehiscent most of their length. All species have an intrastaminal, annular, secretory disk. The styles are longer than the ovary at first; they are massive and connivent in *Spondias dulcis*, but in *Spondias purpurea* they are shorter than the ovary, more discrete and arranged around the periphery of the pistil apex. As in most Anacardiaceae examined to date, the pollen tube grows down a furrow, then channels into separate papilla-lined stylar canals that bypass the micropyle by passing a bridge (ponticulus) through a dorsal outgrowth of the funicle. The stigmas are papillate; they are capitate in *Spondias purpurea* but linear and oblique in *Spondias dulcis* and the remaining Neotropical species. The ovules are crassinucellar, bitegmic, and syntropous (= apotropous), with a long funicle.

As in other Spondioideae, the exocarp is very thick. The homology of the fibrous matrix in *Spondias* is unresolved; it was considered by Lozano (1986a) to be derived from the inner mesocarp, and by Wannan and Quinn (1990) to be part of the endocarp. As conspicuous and economically important as the fruits of *Spondias* are, however, fruit anatomy is poorly studied. An exception is the work of Wannan and Quinn (1990), who used anatomical patterns of the endocarp in Anacardiaceae to detect generic affinities in the family and to characterize subfamily Spondioideae and distinguish it from the other tribes recognized by Engler (1883) that are now joined to form the only other subfamily, Anacardioideae (Pell 2004, Mitchell et al. 2006, Wannan 2006, Pell et al. 2011). Wannan and Quinn (1990) observed that in Spondioideae the sclereids in the endocarp are arranged in an irregular way, without the histologically discrete layers characteristic of the remainder of the family. Within Spondioideae, they found considerable differences among genera in the histology of the mesocarp, which ranges from completely parenchymatous in *Dracontomelon* to completely lignified in *Pleiogynium*; in *Spondias pinnata*, sclereids essentially sheath the resin canals, which are scattered throughout the mesocarp.

### Pollen

Pollen descriptions are available for only three of the species found in the Neotropics: *Spondias mombin*, *Spondias purpurea*, and *Spondias radlkoferi* (see Table 2); moreover, all published studies of *Spondias* pollen have utilized only light microscopy. Studies are needed for more of the species, and SEM studies are needed for examining exine characters.

**Table 2.** Comparison of pollen morphology of three species of *Spondias*; data from Olivera et al. (1998) except where indicated.

Character	*Spondias mombin*	*Spondias purpurea*	*Spondias radlkoferi*
Shape	Spheroidal (prolate to subprolate in Martínez-Hernández et al. 1993)	Subprolate (to spheroidal in Martínez-Hernández et al. 1993)	Spheroidal (prolate in Lozano-García and Martínez-Hernández 1990)
Polar axis (µ)	46–51 (36–45.6 in Martínez-Hernández et al. 1993; 46–56 in Roubik and Moreno 1991)	37–40 (37.6–42.8 in Martínez-Hernández et al. 1993)	35–43 (40–48 in Lozano-García and Martínez-Hernández; 41–45 in Roubik and Moreno 1991)
Equatorial axis (µ)	40–45 (27.3–36 in Martínez-Hernández et al. 1993; 33–44 in Roubik and Moreno 1991)	28–32 (35.2–36 in Martínez-Hernández et al. 1993)	31–36 (28–35.2 in Lozano-García and Martínez-Hernández 1990; 22–33 in Roubik and Moreno 1991)
P/E	1.15	1.3	1.1
Polar diameter (µ)	38–46	30–34	33–37
Exine Surface	Semi-tectate, micro-striate at mesocolpium, microreticulate in polar zone	Semi-tectate, micro-reticulate in polar zone	Semi-tectate, micro-striate
Exine thickness (µ)	1–3	2–2.5	2–2.5 (1–1.6 in Lozano-García and Martínez-Hernández 1990)
Sexine (µ)	1–2	2	1–1.5 (2 in Lozano-García and Martínez-Hernández 1990)
Nexine thickness	0.5	0.5	1
Endoaperture length × height (µ)	13–21 × 4–7	10–16 × 1–4	11–15 × 2–4
Distance between colpi (µ)	10–21	5–12	8–14

The shape is spheroidal to (sub)prolate. Like most Anacardiaceae, *Spondias* pollen is tricolporate. The exine is semi-tectate, and microstriate to microreticulate. Quantitative values are apparently variable within species and generally not diagnostic except for nexine thickness (for *Spondias radlkoferi*) and possibly P/E ratio.

### Hybridization and intermediates

There is a great deal of circumstantial evidence of hybridization in neotropical *Spondias*, almost all of it implicating *Spondias mombin* as one of the putative parents and most reported cases occurring near the range edges of one or both putative parents or where one or both may have been introduced.

Only one of the putative hybrids has been formally recognized, *Spondias × robe* Urban from Haiti (Urban 1929). The type collection, *Ekman 12532* (A, GH, NY, US) as well as one collection each from Cuba (*Schafer 1525*, NY), the Dominican Republic (*Zanoni & Jiménez 44521*), and Costa Rica (*Grayum 11809*, NY) combine the small, obovate leaflets and pink corolla of *Spondias purpurea* with the much more lax inflorescence and very different remaining flower morphology of *Spondias mombin*.

Phylogeographic studies of *Spondias purpurea* and both sympatric and allopatric populations of *Spondias mombin* (Miller and Schaal 2005, Miller 2008) suggest ongoing hybridization between these two species in southern Mesoamerica, although Miller (2008) notes that more studies are needed to determine the relative roles of hybridization versus incomplete lineage sorting.

In northeastern Brazil, local people recognize a variant of *Spondias* locally referred to as “umbu cajá,” which some have conjectured might be a hybrid between *Spondias mombin* and *Spondias tuberosa*. Almeida et al. (2007) conducted a genetic study of this putative hybrid but could find no evidence in the chromosome banding patterns. This entity may be simply a cultivated race of the regionally popular *Spondias tuberosa*.

Conversely, in northwestern Costa Rica isozyme studies of individuals of *Spondias* found some that were morphologically indistinguishable from *Spondias mombin* but whose isozyme bands strongly suggested they were hybrids between *Spondias mombin* and *Spondias radlkoferi* (e.g., *Moran et al. 6293*, NY); R. Moran and J. Hamrick, unpublished results).

In southwestern Amazonia, where *Spondias mombin*, *Spondias globosa*, and *Spondias testudinis* are native and *Spondias purpurea* and *Spondias dulcis* are sparsely cultivated, there is a distinct entity recognized by local people, who call it “cajá açu” (‘large cajá’). This may be a hybrid between *Spondias mombin* and *Spondias testudinis*. The fruits are oblong and lenticellate like *Spondias testudinis*, but larger. As in *Spondias mombin*, the lateral leaflets are relatively broadly oblique-elliptic (vs. obliquely oblanceolate to narrowly elliptic in *Spondias testudinis*), with the margin mostly entire but with a few of the laciniate teeth characteristic of *Spondias testudinis*. This entity has multiple admedial tertiaries arising from the secondaries, whereas in *Spondias testudinis* (as in *Spondias globosa*) the admedial tertiaries are long and composite while in *Spondias mombin* they are random-reticulate.

**Figure 13.**
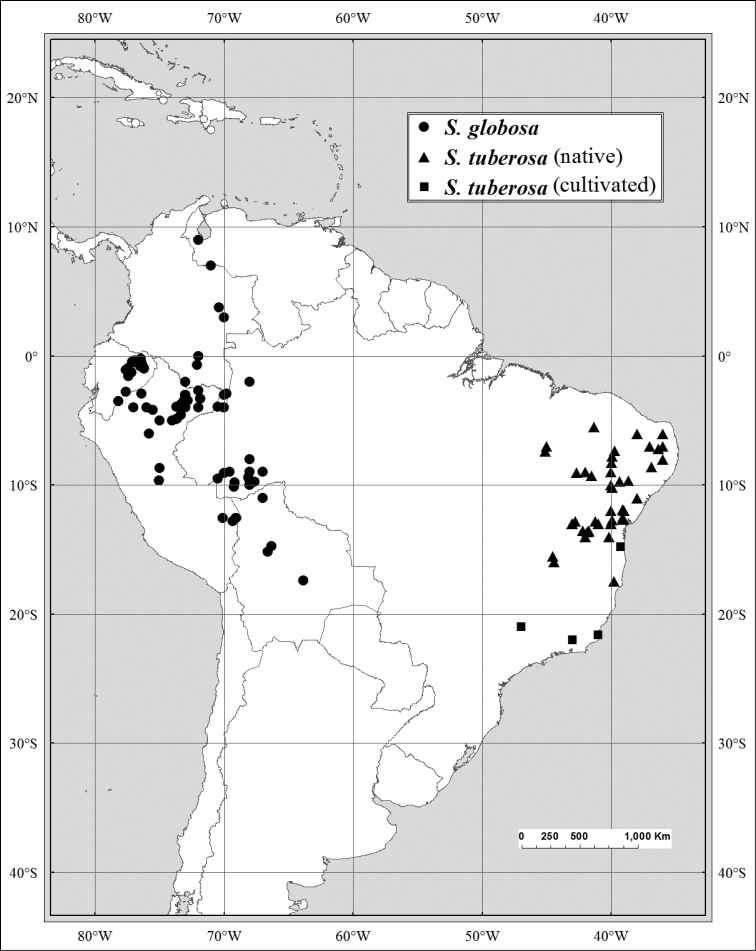
Distributions of *Spondias globosa* and *Spondias tuberosa*.

There is morphological evidence of hybridization between *Spondias mombin* and *Spondias globosa*. The former is widespread and widely cultivated from S Mexico to SE Brazil and eastern central Bolivia, while *Spondias globosa* is more restricted, occurring in W Amazonia and disjunct in Zulia, Venezuela (see Figs 12, 13). In SW Amazonia, the two are usually easy to distinguish, but in Amazonian Ecuador, Colombia, and Peru one frequently finds individuals that appear to be intermediate between the two. Typical *Spondias globosa* has (depressed-)globose fruits, leaflets with composite admedial tertiaries running from the intramarginal vein as well as from the secondaries, and marginal ultimate venation lacking a fimbrial vein and incompletely looped, while *Spondias mombin* has broadly oblong fruits, tertiary venation mostly irregular-reticulate with some admedial branching from the secondaries, and the marginal ultimate venation consisting of a fimbrial vein. We have observed quite a number of specimens (see Index of Specimens Examined) with the fruits of *Spondias mombin* and the leaflet architecture of *Spondias globosa* (although we have not been able to check the marginal ultimate venation of all the specimens).

### Seedlings

There has been some confusion in the literature about terminology related to germination patterns and seedling morphology. Here we use the terminology of Duke (1965) and agree with him that the terms *hypogeal* and *epigeal* have been misapplied to seedlings that are *cryptocotylar* (the cotyledons enclosed in the germinating seed) and *phanerocotylar* (the cotyledons exposed).

The species of *Spondias* occurring in the Neotropics all have hypogeal germination. The seedlings are phanerocotylar (although Macías-Rodríguez and Pérez-Jiménez (1994) report *Spondias purpurea* as cryptocotylar). A tap root and hypocotyl emerge from one end of the fruit; the hypocotyl emerges, curved at first, then carries cotyledons aloft; the cotyledons are opposite, linear, green, sessile, somewhat fleshy; first eophylls opposite (*Spondias purpurea* usually alternate); lateral leaflets of eophylls usually ovate (lanceolate in *Spondias testudinis*, oblanceolate in *Spondias purpurea*), the margin serrate (usually sparsely and regularly so), the teeth sometimes laciniate (*Spondias purpurea*, *Spondias radlkoferi*, *Spondias testudinis*). The patterns are summarized in Table 3.

**Table 3.** Germination patterns and seedling morphology in Neotropical Spondias. [NOTE: table includes only species for which observations/literature are available; unusual character states in bold]

**Taxon**	**Germination pattern**	**First eophylls**	**Lateral leaflets of eophylls**	**Margin**	**Source**
*Spondias dulcis*	Phanerocotylar	**Alternate (photo in reference), 3- or 5-foliolate**	**Lanceolate**	Sparsely and irregularly serrate	Ng 1991
*Spondias globosa*	Phanerocotylar	Opposite, 3-foliolate	Ovate	Sparsely and regularly serrate	*Pennington et al. 17244* (NY)
*Spondias mombin*	Phanerocotylar	Opposite, 3-foliolate	Ovate	Sparsely and regularly serrate	Vogel 1980; Garwood 2009
Spondias purpurea*	**Cryptocotylar**	**Reduced** (the leaflets laminar but nearly scale-like), usually **alternate**, 3-lobed or –foliolate (the lateral lobes or leaflets often alternate)	**Oblanceolate**	Sparsely and regularly serrate, the teeth **often laciniate**	*Magallanes 3887* (NY); Macias-Rodríguez and Péres-Jiménez 1994
*Spondias radlkoferi*	Phanerocotylar	Opposite, 3-foliolate	Ovate	Sparsely and regularly serrate, the teeth **laciniate**	Garwood 2009
*Spondias testudinis*	Phanerocotylar	**5- or 7-foliolate**	**Lanceolate**	Regularly serrate, the teeth **laciniate**	*Daly et al. 7251* (NY)
*Spondias admirabilis*	Phanerocotylar	**5- or 7-foliolate**	**Ovate to lanceolate**	Regularly serrate, the teeth **slightly laciniate**	*Stefano et al. 259* (NY)

[* Our morphology for *Spondias purpurea* disagrees with that of Duke (1965), who reported the first eophylls as opposite and trifoliolate, but as we have a voucher to examine, we suggest that his illustration labeled as *Spondias purpurea* is misidentified.]

### Ecology

A comprehensive review of *Spondias* ecology in tropical America will not be attempted here; some taxon-specific notes are provided under each species. The majority of the ten native species grow primarily in tropical lowland moist forests below 1,000 m elevation, with two exceptions. *Spondias purpurea* naturally occurs in (semi-)deciduous forests, and *Spondias tuberosa* grows in semi-arid deciduous forests called *caatinga arbórea* in NE Brazil. The latter has water-storing tuberous roots, while *Spondias purpurea* stores water in its trunk (Borchert 1994); both remain leafless for long periods of time in the dry season.

Based on the limited amount of research on their floral biology, *Spondias* species are pollinated primarily by Hymenoptera (mostly bees, some wasp species) (Roubik et al. 1986, Nadia et al. 2007); Carneiro and Martins (2012) found that flowers of *Spondias mombin* were pollinated primarily by Scaptotrigona aff. tubiba and Africanized honey bees due to their abundance, behavior, and ability to visit a large number of flowers. The fleshy drupes of *Spondias* are ingested or expectorated, and the unit of dispersal is the endocarp or stone. The mesocarp has a high sugar content (Riba-Hernández et al. 2003), and the fruits are dispersed by a wide diversity of vertebrates, such as medium-sized to large birds (e.g., orioles to chachalacas); various mammals, such as bats, primates, rodents (e.g., agoutis), deer, peccaries, tapirs, coatis, kinkajous, coyotes, and foxes; reptiles (such as ctenosaurs and tortoises) (van der Pijl 1957, Goodwin and Greenhall 1961, Hernández-Camacho and Cooper 1976, Gardner 1977, Husson 1978, Freese and Oppenheimer 1981, Janzen 1985, Fleming 1987, Moskovits and Bjorndal 1990, Handley et al. 1991, Mandujano et al. 1994, Griz and Machado 2001, Vinke et al. 2008, Lobova et al. 2009).

Janzen (Janzen and Martin 1982, Janzen 1985) hypothesized that *Spondias* fruits are among those whose dispersal has been adversely impacted by the extinction of the Pleistocene megafauna (e.g,. gomphotheres, tree sloths, horses, camels), because they are less effectively dispersed (i.e., distance and germination rates diminished).; *Spondias* fruits can be important in the diet of certain vertebrate species (especially during periods of fruit scarcity) and in forest succession or recovery. Some *Spondias* species, such as *Spondias mombin* and *Spondias radlkoferi*, are dispersed into pastures and large clearings and become common in second-growth forests (Janzen 1985).

The gum exudates of some *Spondias* species can be an important constituent of the diet of monkeys such as tamarins (Garber 1985). Bruchid beetles are the primary seed predators of at least two species of *Spondias* (*Spondias radlkoferi* and *Spondias mombin* (Janzen 1980).

Several species of fungal endophytes have been identified in the leaflets of *Spondias mombin*, and these appear to play a role in the production of certain secondary metabolites (Rodrigues and Samuels 1999).

### Economic botany

*Spondias* has a history of use going back at least as far as 6500 B.C., in the Tehuacán Valley of Mexico (Smith 1967). Four species of *Spondias* are economically important in tropical America: *Spondias dulcis*, *Spondias mombin*, *Spondias purpurea* and *Spondias tuberosa*. The economic botany of the commonly cultivated species of *Spondias* has been reviewed extensively in several publications, therefore we present a brief overview after each species with reference to important publications. The vernacular names of *Spondias* species cited in the economic botany literature are often misleading because of incorrect synonymies, erroneous species identifications, uncritical use of previous literature, and faulty equation of common names and scientific names. Approximately 180 common names have been linked to *Spondias purpurea* (Miller and Schaal 2005), while Morton (1981) listed 96 different common names for *Spondias mombin*. In Morton (1981) and Lim (2012), for example, *Spondias purpurea* and *Spondias tuberosa* are confused. For this reason, it is essential to both use and cite vouchers in economic botany and in ethnobotany.

### Conservation

Following the categories and criteria established by the IUCN (http://www.iucnredlist.org/technical-documents/categories-and-criteria/2001-categories-criteria), based on observations in the field and in herbaria, we consider three species of *Spondias* to be at risk of extinction in the short- and medium-term: *Spondias admirabilis* (Endangered), *Spondias expeditionaria* (Critically Endangered), and *Spondias macrocarpa* (Vulnerable). Four species are rather broadly cultivated (*Spondias dulcis*, *Spondias mombin*, *Spondias purpurea* and *Spondias tuberosa*) and so run no risk of extinction, although their genetic diversity maybe becoming compromised due to habitat destruction. *Spondias radlkoferi* and *Spondias venulosa* are cultivated on a limited scale and range.

## Systematic treatment

### Spondias

Taxon classificationPlantaeSapindalesAnacardiaceae

L., Sp. pl. 371. 1753.

Cytheraea Wight & Arn., Prodr. Fl. Ind. Orient. 1: 173. 1834, nom. prov. = *Spondias dulcis* Parkinson.
Wirtgenia Jung. ex Hassk., Flora 27: 624-625. 1844, (non Sch. Bip.) *pro parte quoad Wirtgenia decandra* = *Spondias pinnata* (Koenig ex L.f.) Kurz.
Evia Comm. ex Blume, Mus. Bot. 1(15): 233. 1850. Type: *Evia dulcis* (Parkinson) Comm. ex Blume = *Spondias dulcis* Parkinson.
Warmingia Engl. in Mart., Fl. Bras. 12(2): 281. 1874 (non Rchb. f.). Type: *Warmingia pauciflora* = *Spondias purpurea* L.

#### Type.

*Spondias mombin* L.

#### Description.

**Figure 14.**
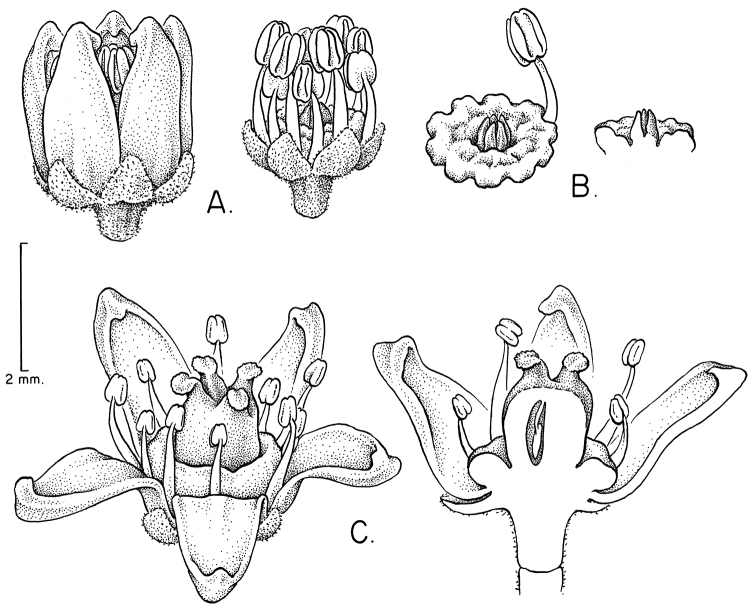
Flowers of *Spondias purpurea*. **A** Staminate flower at anthesis (left), and same with corolla removed **B** Disk, staminode, and stamen **C** Pistillate flower at anthesis (left), and same in longisection through center plus articulated pedicel. **A–B** from *Sidwell et al. 589* (NY) **C** from *Daly 137* (NY).

**Figure 15.**
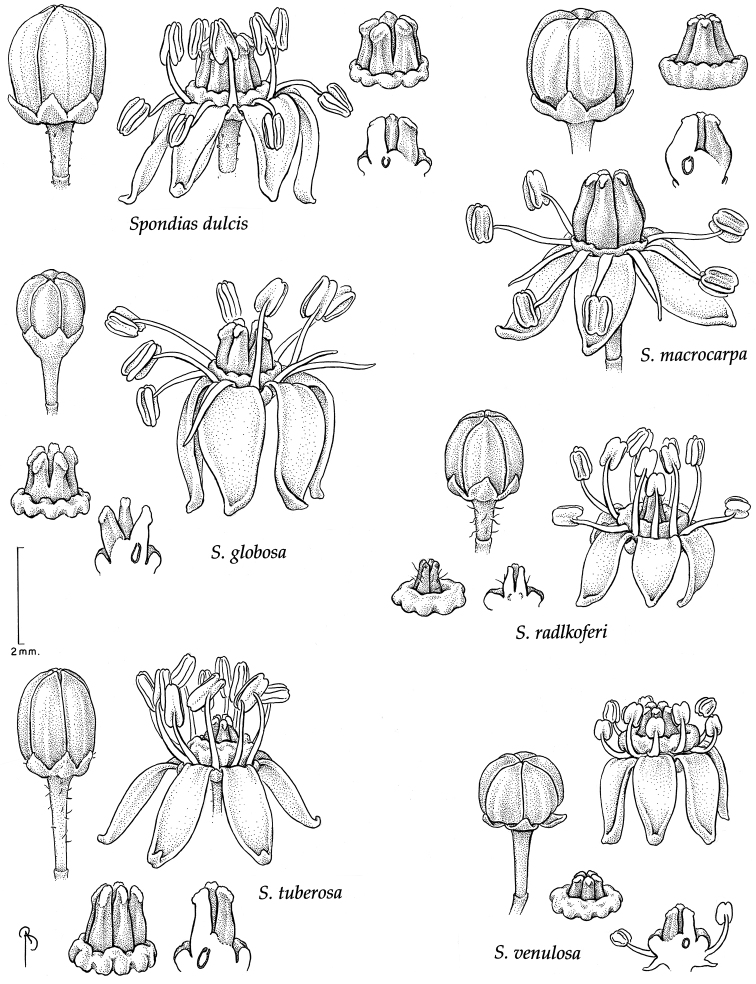
Flowers of *Spondias* species: *Spondias dulcis* (*Wurdack 315*, NY); *Spondias macrocarpa* (*Spada 013/77*, NY); *Spondias globosa* (*Daly et al. 7836*, NY); *Spondias radlkoferi* (*Heithaus 119*, NY); *Spondias testudinis* (*Lao Magín 112*, NY); *Spondias tuberosa* (*Carauta 552*, NY); *Spondias venulosa* (*Queiroz 2604*, NY).

**Figure 16.**
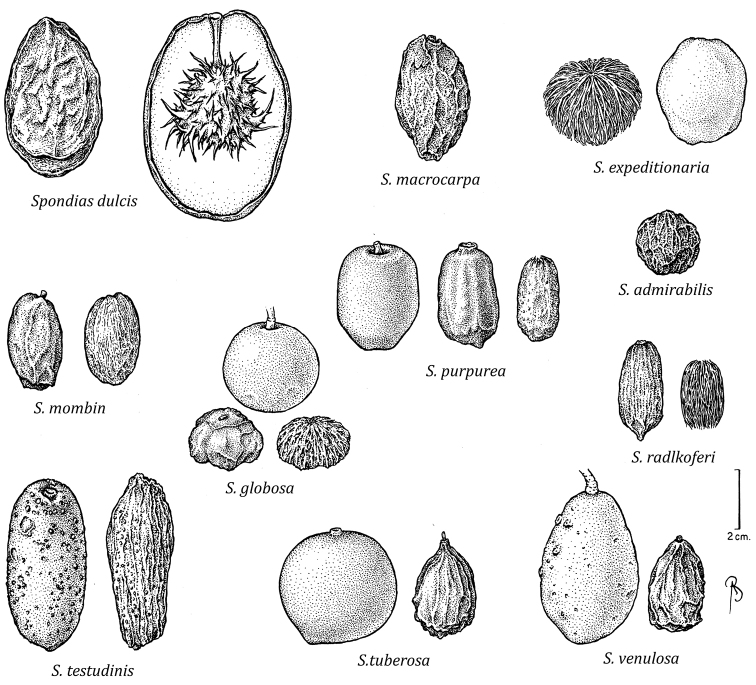
Fruits of Neotropical *Spondias. Spondias dulcis* (dry fruit: *Berlin 870*, NY; and longisection: unvouchered photo), *Spondias macrocarpa* (dry fruit: *Thomas et al. 6823*, NY), *Spondias expeditionaria* (endocarp and fresh fruit: *Lorenzi s.n.*, NY), *Spondias mombin* (dry fruit: *Mostacedo 2941*, NY; endocarp: *Moraes 1046*, NY), *Spondias globosa* (fresh fruit [top], dry fruit, and endocarp: *Daly et al. 8472*, NY), *Spondias purpurea* (fresh fruit: unvouchered photo; dry fruit: *Moreno 2827*, NY; endocarp: *Magallanes 3887*), *Spondias admirabilis* (dry fruit: *Farney et al. 4046*, NY), *Spondias radlkoferi* (dry fruit: *Nee 6729*, NY; endocarp: *Moran 6291*, NY), *Spondias testudinis* (fresh and dry fruit; *Daly et al. 7559*, NY), *Spondias tuberosa* (fresh fruit: from Morton, 1987; dry fruit: *Mattos Silva 2299*, NY), and *Spondias venulosa* (fresh fruit: from photo in Lorenzi, 1998; dry fruit: *Araújo 7828*, NY).

**Figure 17.**
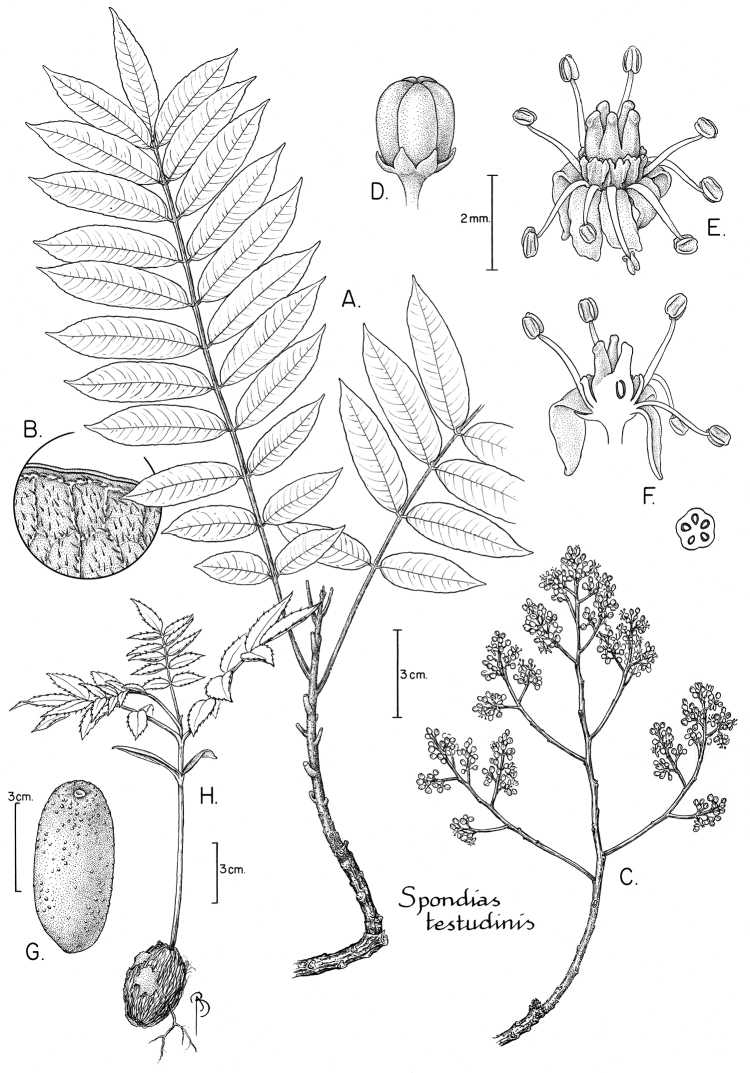
*Spondias testudinis*. **A** Branchlet **B** Detail of leaflet blade and margin **C** Inflorescence **D** Bud **E** Flower at anthesis **F** Longisection through same at center **G** Fresh fruit **H** Seedling. **A, B** from *Lao Magín 112* (NY) **C–F** from *Lao Magín 83* (NY) **G** from specimen and field photo of *Daly et al. 7559* (NY) **H** from specimen and field photo of *Daly et al. 7251* (NY).

Small to large *trees* (rarely shrubby and broadly branching), usually hermaphroditic (except *Spondias purpurea*). Simple, thick plank buttresses to 100 cm high sometimes present. *Outer bark* brown or usually gray, densely to broadly fissured, sometimes thick, usually rough, often with raised lenticels, rarely (some *Spondias mombin* and *Spondias purpurea*) with large, corky, tooth-like projections. *Inner bark* usually broadly striate (white and rose, red, orange, or brown). *Resin* viscous and usually clear or less often cloudy (*Spondias globosa*). *Trichomes* of various simple hairs and capitate glandular hairs. *Leaves* alternate, aggregated toward branch tips, sometimes deciduous (sometimes facultatively so), usually imparipinnate, petiolulate; leaflets (sub)opposite, the apex often apiculate; margin entire/cren(ul)ate/serr(ul)ate (Figs 2–4), the sinuses often glandular; intramarginal vein present (see Figs 1–10 for leaflet venation); intercostal tertiaries usually irregular-reticulate and often branching admedially, areoles often poorly developed; freely ending veinlets dendritic, often highly branched, leaflets often glandular-punctate. *Inflorescences* produced either before leaf flush (e.g., *Spondias purpurea*) or during leaf flush, terminal or axillary, paniculate (reduced to a pseudoracemose botryoid in *Spondias purpurea*, Barfod 1988); inflorescence bracts subtending secondary axes early-caducous; pedicel articulated. *Flowers* usually hermaphroditic but strongly protandrous (dioecious in *Spondias purpurea*, Fig. 14), pentamerous, calyx green (often red in *Spondias purpurea*), shorter than the disk, lobes usually apert (quincuncial at the base and valvate at the apex where they meet in *Spondias purpurea*, Bachelier and Endress 2009); corolla valvate, petals yellowishwhite (usually red in *Spondias purpurea*); patent or strongly reflexed at anthesis (spreading in *Spondias purpurea*), often terminating in a swollen and strongly inflexed apiculum, the margin papillate; stamens (8)10, in two unequal series, outer series opposite the carpels, filaments linear to subulate, glabrous, anthers dorsifixed, yellow; disk intrastaminal, annular, summit and outer margin variously shaped (Fig. 15), usually yellow or less often pink (some *Spondias purpurea*) or purple (some *Spondias radlkoferi*); pistillode (*Spondias purpurea* only) reduced to 3–5 subulate styles; at anthesis the ovary usually rudimentary, developing after the anthers dehisce (Fig. 11), pistil (3–) 5locular, surmounted by (3–)5 short, free styles (*Spondias purpurea*) or usually the styles (broadly) subulate and separate at least half the length of the pistil, connivent at least basally, stigmas usually extrorse (introrse in *Spondias purpurea* and *Spondias radlkoferi*), discoid to linear; each locule with one apical ovule, the ovules crassinucellar and bitegmic, styles separating as fruit develops. *Fruit* (Fig. 16) a (1) 3–5-seeded, green, yellow, orange, or red drupe, oblong, (depressed-)globose, obovoid, or ellipsoid, remnants of styles widely separated and often visible in developing fruits; exocarp usually smooth (densely lenticellate in *Spondias testudinis*); mesocarp fleshy, edible, sweet and sour, endocarp bony and enveloped by a fibrous matrix (with spiny projections and growing over apex of pedicel in *Spondias dulcis*), at maturity the fruit often breaking away at articulation of pedicel. *Pollen* (based on Roubik and Moreno P. 1991): 41-56 × 22-44 µ, prolate or subprolate, pores 6–15 µ diam. *Seedlings*: germination hypogeal, tap root and hypocotyl emerge from blunt end of fruit; hypocotyl emerges, curved at first, then carries cotyledons aloft; phanerocotylar; cotyledons opposite, linear, green, sessile, somewhat fleshy; first eophylls opposite and imparipinnate (*Spondias testudinis*, Fig. 17H) or trifoliolate (pers. obs., Vogel 1980, Garwood 2009). *Chromosomes*: n = 16, 2n = 32 (Simmonds 1954, Bawa 1973, Mehra 1976, Guerra 1986, Pedrosa et al. 1999, Almeida et al. 2007).

Of the ca. 18 species in *Spondias*, ten are native to the New World, distributed from Mexico to southern Brazil, one is native to Madagascar, and seven are native to Asia and the South Pacific, from Malesia (sensu *Flora Malesiana*) to tropical China, Sri Lanka, Indochina, Thailand, India (except extreme north), Myanmar (Burma), Solomon Islands east to Polynesia. *Spondias dulcis* is cultivated in tropical America and the Antilles; *Spondias mombin* and *Spondias purpurea* are both introduced throughout Tropical West Africa and Asia (and in the West Indies, where they are not found in primary vegetation and may not be native); *Spondias mombin* is often adventive in Tropical West Africa.

Some of the species native to the Neotropics have restricted distributions. *Spondias testudinis* is restricted to southwestern Amazonia, and *Spondias admirabilis* and *Spondias expeditionaria* both are known from very few localities in Brazil’s Atlantic Coastal Forest, while the other two Atlantic Forest species, *Spondias macrocarpa* and especially *Spondias venulosa*, are somewhat more broadly distributed in that region; both *Spondias expeditionaria* and *Spondias macrocarpa* are rare as well. The natural distribution of *Spondias tuberosa* is the arid *caatinga* vegetation of Northeastern Brazil, and *Spondias globosa* is a Western Amazon species. *Spondias radlkoferi* ranges from Mexico through Central America to Colombia and NW Venezuela; there is an unconfirmed report from Los Ríos in W Ecuador (Dodson 8837, MO). *Spondias purpurea* is native to N Mexico through Central America and may be native to SW Ecuador. *Spondias mombin* is native to moist forests through much of northern South America, although it is uncertain whether the populations in Brazil’s Atlantic Coastal Forest are native.

### Key to *Spondias* in the Neotropics based on flowering and/or fruiting material

**Table:** 

1	Leaflet apex usually obtuse to retuse, occasionally acute; usually flowering before leaf flush; often ramiflorous, inflorescence pseudoracemose (botryoid); flowers unisexual; sepals rotund to ovate; petals red to purple (yellow in one cultivar), spreading to suberect at anthesis; stigmas slightly introrse as ovary develops; fruit maturing red to purple (yellow in one cultivar); native to tropical dry forests from N Mexico to SW Ecuador, also widely cultivated and adventive in the tropics	***Spondias purpurea***
–	Leaflet apex acute to acuminate, rarely obtuse (some individuals of *Spondias radlkoferi*); flowering with or after leaf flush; inflorescence a much-branched panicle; flowers strongly protandrous but hermaphrodite; sepals deltoid, less often triangular or ovate; petals white to cream to greenish-yellow, patent to reflexed at anthesis; stigmas extrorse as ovary develops; fruit maturing yellow to orange(-brown) to green; central Mexico to Paraguay	**2**
2	Leaves always glabrous; lateral leaflets usually medially (sub)symmetrical (base usually (sub)symmetrical), anthers not entirely exceeding pistil at anthesis; stone continuous with pedicel, the endocarp lacking a fibrous matrix, provided with spiny projections; widely cultivated introduction from Oceania	***Spondias dulcis***
–	Leaves usually with at least scattered trichomes on the petiolules, basal part of leaflet margin, and/or basal part of abaxial surface; lateral leaflets usually medially asymmetrical (base usually oblique)(subsymmetrical in *Spondias tuberosa*); anthers entirely exceeding pistil at anthesis; stone free from pedicel at maturity, endocarp with a fibrous matrix, lacking spiny projections; Neotropical species but some widely cultivated	**3**
3	Shrubby trees, often broader than tall, with tortuous branching, short shoots often present; bark sparsely and shallowly fissured; roots tuberous; leaves 1–3 (4)-jugate; midvein of leaflet flat to prominulous abaxially; tertiary veins taper (lose gauge) at both ends; endocarp laterally compressed and very slightly 1-carinate, smooth; native to *caatinga* vegetation of NE Brazil (but cultivated in SE Brazil)	***Spondias tuberosa***
–	Trees without tortuous branching, short shoots absent; bark more densely and deeply fissured; roots not tuberous; leaves 3–14-jugate, midvein of leaflet prominent abaxially (sometimes prominulous in *Spondias radlkoferi* and *Spondias admirabilis*); tertiary veins not tapered at both ends; endocarp not laterally compressed nor carinate; central Mexico to Paraguay and E Brazil	**4**
4	Intramarginal secondary vein sometimes (sub)marginal; costal secondary veins usually distinctly arcuate, sometimes with hairy tuft domatia in the axils abaxially; tertiary veins alternate-percurrent and random-reticulate; calyx divided nearly to base and sepals slightly imbricate at base, disk markedly papillate, pistil sometimes pubescent; fruit maturing green (rarely orange), obovoid with apex abruptly short-acuminate (dry); tropical dry and moist forests, S Mexico and Central America to NW Venezuela and W Ecuador	***Spondias radlkoferi***
–	Intramarginal secondary vein always removed from margin; costal secondary veins essentially straight to very slightly arcuate, without hairy tuft domatia; tertiary veins random-reticulate and/or admedially ramified; calyx not divided to base and not imbricate, disk not markedly papillate, pistil always glabrous; fruit maturing yellow or orange(-brown), oblong to ellipsoid to globose, apex rounded to truncate; S Mexico to Paraguay and E Brazil	**5**
5	Lateral petiolules (2) 3–10 mm long; margin of adult leaflets (sub-)entire (very rarely crenulate or serrulate) and usually flat; trichomes on leaflets all straight and short (not exceeding 0.3 mm), glandular trichomes absent	**6**
–	Lateral petiolules 0–3 mm long; margin of adult leaflets sparsely serr(ul)ate or crenate, usually slightly revolute; on leaflets the hairs flexuous or uncinate, 0.3–0.6 mm long, glandular trichomes often present	**7**
6	Outer bark often with corky tubercular or spinose projections in sunny growing conditions; inner bark pale red to pink to orange; leaves 3–7 (–10)-jugate; leaflets with tertiary veins primarily irregular-reticulate, some admedial branching, fimbrial vein present; fruits oblong or less often ellipsoid or slightly oblong-ovoid; native to S Mexico S to Paraguay & E Brazil, widely cultivated in the moist tropics	***Spondias mombin***
–	Outer bark without spinose projections; inner bark usually (pale) red-and-white striate; leaves 3–5 (–7)-jugate; leaflets with composite admedial tertiaries branching from at or near the intramarginal vein, fimbrial vein absent; fruits globose to perdepressed-ovoid; W Amazonia plus Zulia and Barinas in Venezuela	***Spondias globosa***
7	Leaves (5) 7–16-jugate, leaf rachis densely pubescent; composite admedial tertiary veins absent; anthers 0.7–0.9 mm long, annular disk much taller than thick	**8**
–	Leaves 3–5 (7)-jugate, leaf rachis glabrous or sparsely pubescent; composite admedial tertiary veins present; anthers 0.4–0.65 (0.75) mm long, annular disk nearly as thick as tall	**10**
8	Lateral leaflets obliquely oblanceolate to elliptic, leaflet base insertion decurrent; fruit (3.8) 4.9–6.3 cm long (dry), exocarp surface with sparse but conspicuous raised lenticels; SW Amazonia	***Spondias testudinis***
–	Lateral leaflets (oblong-)lanceolate, sometimes falcate, leaflet base insertion excurrent; fruit 3.1–4 cm long, the exocarp surface smooth or rarely with sparse flat lenticels; SE Brazil	**9**
9	Leaflet base asymmetric, acute to obtuse; secondary vein insertion decurrent; intersecondary veins (when present) parallel to secondaries; inflorescence pubescence consisting of scattered erect hairs; disk not markedly dissected nor sulcate; fruits (ob)ovoid to ellipsoid, not markedly costate	***Spondias macrocarpa***
–	Leaflet base subsymmetric and cordate to truncate; secondary vein insertion excurrent or abruptly decurrent; intersecondary veins (when present) perpendicular to midvein; inflorescence pubescence consisting of scattered glandular hairs; disk markedly dissected and sulcate; fruits (depressed-) globose, usually slightly 5-costate	***Spondias expeditionaria***
10	Leaflets usually glossy and chartaceous to coriaceous, secondary vein pairs 11–18, on abaxial surface the midvein usually narrowly prominent, on adaxial surface the secondaries narrowly prominulous; margin subentire, less often broadly & irregularly crenulate, revolute usually only at base; flower pedicel 2.5–2.7 mm long; calyx lobes spreading in bud; stamens inflexed, the filaments 0.5–0.8 mm long; fruits 3.6–6 × 1.9–3.6 cm, slightly oblong or less often (ob)ovoid, when fresh the surface often pitted	***Spondias venulosa***
–	Leaflets dull and chartaceous to membranaceous, secondary vein pairs 7–13, on abaxial surface the midvein usually sunk in a groove, on adaxial surface the secondaries obscure; margin sparsely serrulate, slightly revolute; flower pedicel 0.7–1.4 mm long; calyx lobes appressed in bud; stamens spreading, the filaments 1–1.7 mm long; fruits 1.8–2.8 (3.2) × 1.8–2.2 cm, (depressed-)globose to oblong or slightly obovoid	***Spondias admirabilis***

### Key to *Spondias* in the Neotropics based only on adult leaflet characters

**Table:** 

1	Intramarginal vein sometimes absent (marginal secondary present instead); leaflet apex sometimes obtuse, rounded, or retuse; hairy tuft domatia often present in the axils of secondary veins abaxially; veinlets terminating in highly branched sclereids	***Spondias radlkoferi*** (*pro parte*)
–	Intramarginal vein always present (sometimes submarginal); leaflet apex acuminate (*Spondias purpurea* sometimes acute, obtuse, or retuse); hairy tuft domatia absent; veinlets not terminating in highly branched sclereids	**2**
2	At leaflet base, margin revolute and abaxial surface densely provided with erect to flexuous hairs 0.4–0.6 (0.8) mm long	***Spondias venulosa***
–	Leaflet base not notably revolute relative to rest of margin, abaxial leaflet surface glabrous or with shorter hairs	**3**
3	Lateral leaflets broadly elliptic; leaflets with neither inter-secondary veins nor epimedial tertiary veins	***Spondias tuberosa***
–	Lateral leaflets narrowly elliptic, oblong-elliptic, or lanceolate; leaflets with inter-secondary veins and/or epimedial tertiary veins	**4**
4	Intersecondary and/or epimedial tertiary veins perpendicular to midvein; secondary vein course spreading to arcuate (*Spondias globosa* and *Spondias macrocarpa* with secondary vein course straight to slightly arcuate)	**5**
–	Intersecondary and/or epimedial tertiary veins not perpendicular to midvein	**7**
5	Leaflets medially symmetric; base (sub)symmetric; basal insertion excurrent; leaflet margin consistently with at least some teeth, the teeth convex-convex	***Spondias expeditionaria***
–	Leaflets medially asymmetric; base asymmetric; basal insertion decurrent; leaflet margin sometimes or usually entire; teeth (when present) concave-convex	**6**
6	Leaflet acumen 0–5 mm long; leaflet base insertion symmetric; freely ending veinlet (FEVs) highly branched; highly branched sclereids absent	***Spondias purpurea***
–	Leaflet acumen 3–14 mm long; leaflet base insertion asymmetric; FEVs 2–3-branched, terminating in highly branched sclereids	***Spondias radlkoferi*** (*pro parte*)
7	Lateral leaflets medially symmetric, the base (sub)symmetric; secondary veins nearly perpendicular to midvein	***Spondias dulcis***
–	Lateral leaflets medially asymmetric, the base asymmetric; secondary vein angle acute to midvein (sometimes nearly perpendicular in *Spondias macrocarpa*)	**8**
8	Leaves (5) 7–13-jugate; petiole and rachis densely pubescent; lateral leaflets lanceolate; teeth (when present) concave-convex	**9**
–	Leaves (1) 3–6 (7)-jugate (up to 10 (12) juga in *Spondias mombin*); petiole and rachis glabous or sparsely pubescent; lateral leaflets ovate; margin entire (*Spondias globosa*, *Spondias mombin*) or teeth (when present) convex-convex (*Spondias admirabilis*);	**10**
9	Secondary veins in 10–15 pairs, angle usually irregular; leaflet base insertion (sub)symmetric; inter-secondary veins and/or epimedial tertiaries sporadically present, on average less than one per pair of successive intercostal secondaries	***Spondias macrocarpa***
–	Secondary veins in 15–17 pairs, angle uniform; leaflet base insertion often asymmetric; inter-secondary veins consistently present, ca. one per pair of successive intercostal secondaries	***Spondias testudinis***
10	Leaflet base insertion symmetric; on mature leaflets the margin sparsely serrulate; secondary vein spacing irregular; tracheoid idioblasts present	***Spondias admirabilis***
–	Leaflet base insertion usually asymmetric; on mature leaflets the margin consistently entire; secondary vein spacing decreasing toward base and (*Spondias globosa*) apex; tracheoid idioblasts absent	**11**
11	Leaflet basal insertion decurrent; secondary veins straight to slightly arcuate, composite admedial tertiaries branching from at or near the intramarginal vein, marginal ultimate veins usually incompletely looped	***Spondias globosa***
–	Leaflet basal insertion excurrent; secondary veins straight, tertiary venation primarily irregular-reticulate, some admedial branching, fimbrial vein present	***Spondias mombin***

### Spondias admirabilis

Taxon classificationPlantaeSapindalesAnacardiaceae

J. D. Mitch. & Daly sp. nov.

urn:lsid:ipni.org:names:77148927-1

**Figure 18.**
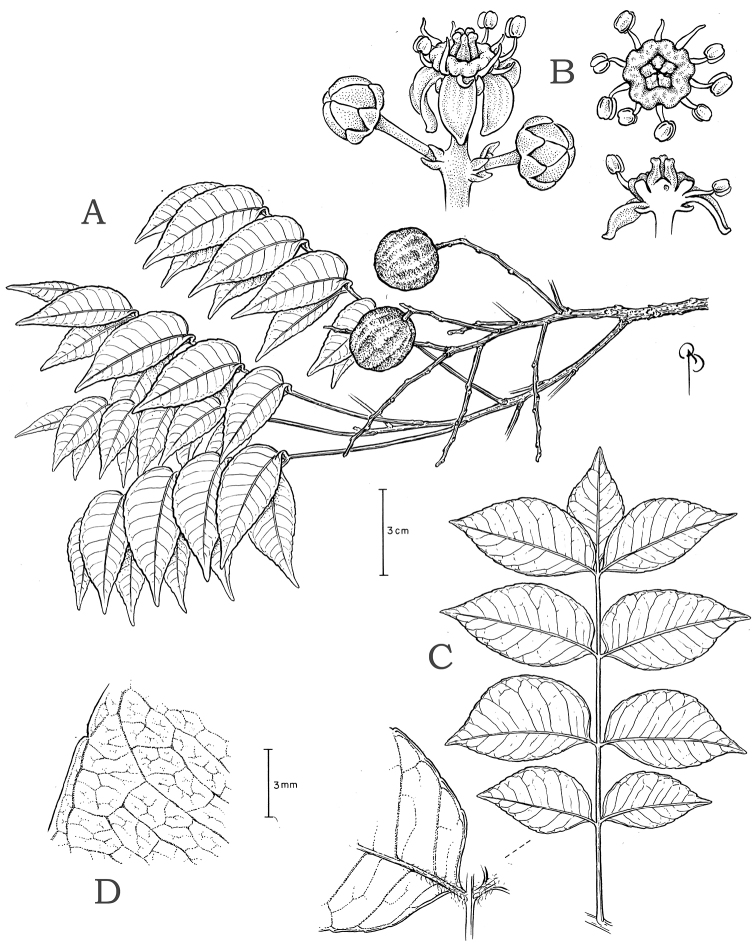
*Spondias admirabilis*. **A** Fruiting branchlet **B** Cymule with flower at anthesis (left); top view of stamens, disk, and immature pistil (right top); and longisection at center of flower with more of stamens removed **C** Whole leaf (right) and rachis plus leaflet bases (inset) **D** Leaf venation detail. A from *Farney et al. 3957* (NY). B from *Lanna Sobrinho 1587* (NY). C–D from *Farney et al. 4172* (NY).

**Figure 19.**
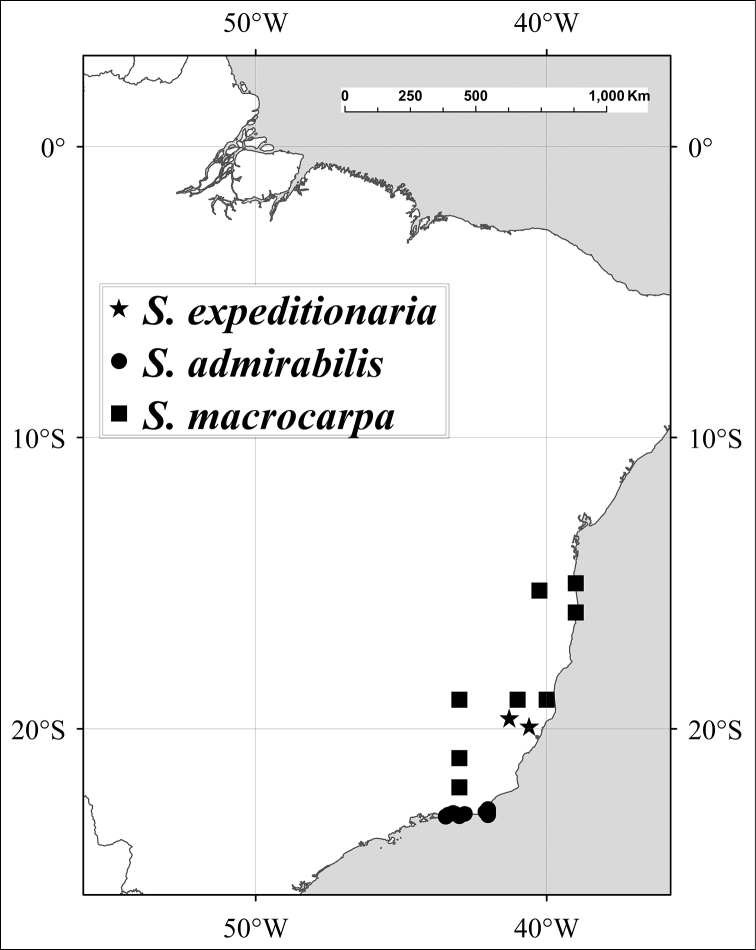
Distributions of *Spondias admirabilis*, *Spondias expeditionaria*, and *Spondias macrocarpa*.

Figs 8 , 13 , 16 , 18 , 19

#### Diagnosis.

Tree to 25 m tall in the Mata Atlântica Complex of Brazil, similar to *Spondias venulosa* (Mart. ex Engl.) Engl. because the leaves mostly 3–5-jugate, some parallel intersecondary veins present, and composite admedial tertiary veins, and flower annular disk short and thick; S. admirabilis differs by the leaflets chartaceous to membranaceous and dull (vs. coriaceous and glossy), the margin slightly revolute and serrulate (vs. revolute only at base and entire or less often broadly crenulate), 7–13 (vs. 10–20) secondary vein pairs, stamens 1.5–2 and 1.4–1.95 (vs. 1.2–1.3 and 0.9–1.1) mm long, and the fruit 1.8–3.2 (vs. 3.6–6) cm long, depressed-globose (vs. oblong or less often obovoid).

#### Type.

BRAZIL. Rio de Janeiro: Ponta da Jararaca, 13 Apr 2000, C. Farney & J. C. Gomes 4046 (holotype: RB!; isotype: NY!).

#### Description.

*Hermaphroditic trees*, reproductive height 4–15 (25) m. Trunk up to 48 (70) cm diam.; *outer bark* rugose. *Trichomes* of three types: fine straight to flexuous (sometimes crispate) white hairs to 0.8 mm long; thick orange glandular hairs to 0.05 mm long; and erect white hairs to 0.1 mm long. *Leaves* 3–5 (7)-jugate, 7.5–18 cm long; petiole (0.6) 2.6–4.4 cm long, petiole and rachis usually glabrous or with sparse flexuous hairs, denser near petiolules; petiolules densely pubescent, lateral petiolules 1–2 mm long, terminal petiolule 6–8 (1.2) mm long; basal leaflets 1.7–3.8 × 1.1–2.3 cm, ovate, other laterals 2.4–5.7 × 1.2–2.4 cm, ovate to lanceolate or less often elliptic, terminal leaflet 2.7–3.6 × 1.5–2 cm, (broadly) ovate or less often lanceolate; leaflet apex abruptly to gradually acuminate, the acumen 3–10 mm long, broad or narrow, mucronate; lateral lamina medially and basally asymmetrical, the acroscopic side ovate or less often elliptic with truncate to cordate base, the basiscopic side elliptic or less often lanceolate with acute to truncate base, basal insertion symmetrical and abruptly decurrent; leaflet margin usually slightly revolute, notably at base, sparsely serrulate, with a few blunt convex-convex teeth, the sinus sometimes vascularized; leaflets chartaceous to membranaceous, both surfaces dull. *Inflorescences* (sub-)terminal on leafy branches, 4.6–18.5 cm long, 2.1–2.5 mm diam at base, broadly branched, the secondary axes to 14.5 cm long, the axes glabrous or sometimes with sparse to dense erect white hairs to 0.1 mm long and scattered glandular hairs; bracts often semi-clasping, those subtending primary axes 1.5–2 mm long, those subtending secondary axes 1–1.5 mm long, all narrowly subulate to lorate, apex caudate, bracteoles 0.4–0.7 mm long, ovate to lanceolate, apex acute, margin of bract(eole)s sometimes ciliate, with or without scattered glandular hairs; pedicel 0.8–2.2 mm long overall, portion distal to the articulation 0.6–1.3 mm long, glabrous. *Calyx* 0.5–0.75 mm long overall, aestivation apert, often divided nearly to base, the lobes 0.35–0.65 mm long, narrowly ovate to deltate, usually acuminate, glabrous, the margin sometimes with a few scattered capitate glandular hairs; petals 1.75–2.5 × 0.9–1.2 mm, narrowly ovate, the apex acute to slightly acuminate, white, glabrous, reflexed at anthesis; stamens spreading, the antesepalous and antepetalous ones 1.5–2 mm and 1.4–1.95 mm long, respectively, the anthers 0.5–0.75 mm long, yellow, in dorsiventral view broadly ovate, in lateral view broadly elliptic or oblong-elliptic; disk 0.35–0.5 mm tall, (0.1) 0.25–0.4 mm thick, yellow, the summit undulate and outer margin deeply sulcate; pistil 0.7–1.5 mm long, slightly ovoid, divided nearly to the base into slightly subulate, connivent styles, the stigmas extrorse, discoid. *Fruits* 1.8–2.8 (3.2) × 1.8–2.2 cm diam when dry, (depressed-) globose to oblong or obovoid, maturing yellow, the surface glabrous, smooth, and dull. *Germination*: Phanerocotylar, hypogeal (*Stefano et al. 259*, NY). *Seedlings*: First eophylls opposite, trifoliolate, margin serrate (*Stefano et al. 259*, NY).

*Leaflet venation*: Fimbrial vein absent; secondary veins 7–13 pairs, rather straight, spacing irregular, angle decreasing toward apex and often increasing toward base, insertion decurrent; some intersecondaries present and parallel to secondaries; intercostal tertiaries admedially ramified, with frequent composite admedials and very little reticulation; areolation barely developed (mostly at tertiary rank); FEVs 4-5+-branched, somewhat dichotomous, terminating in tracheoid idioblasts; marginal ultimate venation incompletely looped; on abaxial side the midvein often very narrowly prominulous and usually sunk in a groove, secondary veins usually flat (sometimes prominulous) but drying discolorous, densely pubescent at base; on adaxial side the midvein narrowly prominulous to prominent, remainder of the veins flat, those above secondary rank obscure, sparsely pubescent on the midvein at base, glabrescent distally.

#### Distribution.

Restricted to Mata Atlântica (Atlantic coastal forests) complex in the state of Rio de Janeiro, Brazil.

#### Ecology.

*Spondias admirabilis* is a small to relatively large tree of semi-deciduous slope forests, moist forests, secondary forests, closed shrubby vegetation, and open rocky areas, between 100–300 m. It is known to flower Apr–Nov and fruit Oct–June.

#### Common names.

Brazil, Rio de Janeiro: cajá-mirim (Lanna Silva 1587, RB); cajazeira(o) (Glaziou 17584, RB), cajá miúdo (Rohan 95, R).

#### Etymology.

The specific epithet (Latin for “remarkable” or “astonishing”) expresses our surprise at discovering a new species of *Spondias* dispersed among collections of two other species from a region that is relatively well-known.

#### Specimens examined.

**BRAZIL**. **Rio de Janeiro**: Niterói, Itaipuaçu, Pico Alto Moirão, 14 Jan 1982, Andreata et al. 357 (NY, RB), Maricá, Pico Alto Moirão, 9 Sep 1982, Andreata et al. 504 (NY, PEUFR, RB); between Mun. Rio de Janeiro and Niterói, Maricá, Itaipuaçu, Alto Moirão, elev. 160 m, 20 Sep 1989, Andreata et al. 915 (NY, RB); Gávea, Parque da Cidade, 8 Oct 1986, Angeli 703 (NY); Mun. Arraial do Cabo, Morro Miranda, 23 Sep 1987, Araújo & Souza 8229 (NY); Mangaratiba, RPPN Rio das Pedras, trail to Pico do Corisco, 13 Jun 2000, Bovini et al. 1878 (NY, RB); Mun. Arraial do Cabo, Morro do Miranda, 12 Jan 2000, Farney et al. 3957 (NY, RB), Mun. São Pedro da Aldeia, Serra de Sapiatiba, road to the tower, elev. 100–300 m, 11 Sep 2000, Farney & Gomes 4172 (NY, RB); Alto do Rio Comprido, Barão de Mesquita, 14 Sep 1879, Franklin s.n. (R-73729, R s.n.; LISU-5368); Morro do Inglez [sic], Corcovado (cultivated), 22 Oct 1866, Glaziou 17584 (P, R); Estrada de Ferro Central do Brasil, 24 Jul 1932, Inspectoria Florestal 35 (R-27641-2 sheets); Jacarepaguá, road to Barra, 12 Oct 1967, J. Lanna Sobrinho 1587 (GUA, NY, R); Mun. Rio de Janeiro, Restinga do Recreio dos Bandeirantes, Morro da Prainha, elev. 0–90 m, 4 Dec 1978, Martinelli 5552 (NY, RB); Rio de Janeiro, Horto Botânico [behind Jardim Botânico], 29 Sep 1915, Piery s.n. (R-73773, R s.n.-2 sheets; LISU-5419); Mun. Cabo Frio, road to Armação dos Búzios, 9 Jan 1985, Pirani & Zappi 1019 (NY, SPF); Armação de Búzios, Serra das Emerências, 17 Oct 2004, Ribeiro & Dantas 350 (NY, RB); Jardim Botânico do Rio de Janeiro, Seção XI, Canteiro F, 5 Nov 1984, Ricardo 600 (NY, RB); Sete Pontes, 12 Apr 1878, Rohan 95 (R-73731, R-73770); Horto Florestal do Jardim Botânico do Rio de Janeiro, 22°58'07"S, 43°13'48"W, 8 Mar 2011, Stefano et al. 250 (NY, RB), Barra de Guaratiba, trail to Praia do Meio, 23°03'48"S, 43°33'42"W, 8 Mar 2011, Stefano et al. 259 (NY, RB); Niterói, Grota Funda road, near Parque Darcy Ribeiro, 22°54'44"S, 43°02'08"W, 9 Mar 2011, Stefano et al. 260 (NY, RB); SE slope of Serra da Piaba, 5 Apr 1972, Sucre 8770 (GUA, NY, RB).

#### Conservation status.

We propose to classify this species as Endangered, with the following justification: It is restricted to Rio de Janeiro state, it is a lowland species in a region where the lowland forests are highly fragmented, and it is not common, considering that it is represented by relatively few collections in a region that has been rather well sampled botanically.

#### Discussion.

*Spondias admirabilis* resembles another Atlantic Forest species, *Spondias venulosa*, because both have few pairs of leaflets (mostly 3–5), decurrent leaflet base insertion, some parallel inter-secondary veins, composite admedial tertiary veins, the flower disk short and thick, and the carpels divided nearly to base. The former differs by having the leaflets chartaceous to membranaceous and dull (vs. chartaceous to coriaceous and glossy), the margin slightly revolute and serrulate (vs. revolute only at base and entire or less often broadly crenulate), 7–13 secondary vein pairs (vs. 10–20), the FEV branching somewhat dichotomous (vs. dendritic), the marginal ultimate venation incompletely looped (vs. mostly looped), bracts on the primary inflorescence axes shorter (1.5–2 vs. 3.5–6.5 mm), flower pedicel shorter (0.8–2.2 vs. 1.8–3.5 mm), stamens longer (1.5–2 and 1.4–1.95 mm vs. 1.2–1.3 and 0.9–1.1 mm), and fruit much smaller (1.8–2.4 × 1.8–2.2 cm vs. 3.6–6 × 1.9–3.6 cm) with smooth surface (vs. often shallowly pitted and very sparsely lenticellate). Based on the exsiccatae, the two species may be sympatric in the state of Rio de Janeiro at Serra de Sapiatiba, Alto Moirão, Sete Pontes, and possibly Búzios.

### Spondias dulcis

Taxon classificationPlantaeSapindalesAnacardiaceae

Parkinson, J. voy. South Seas 39. 1773.

**Figure 20.**
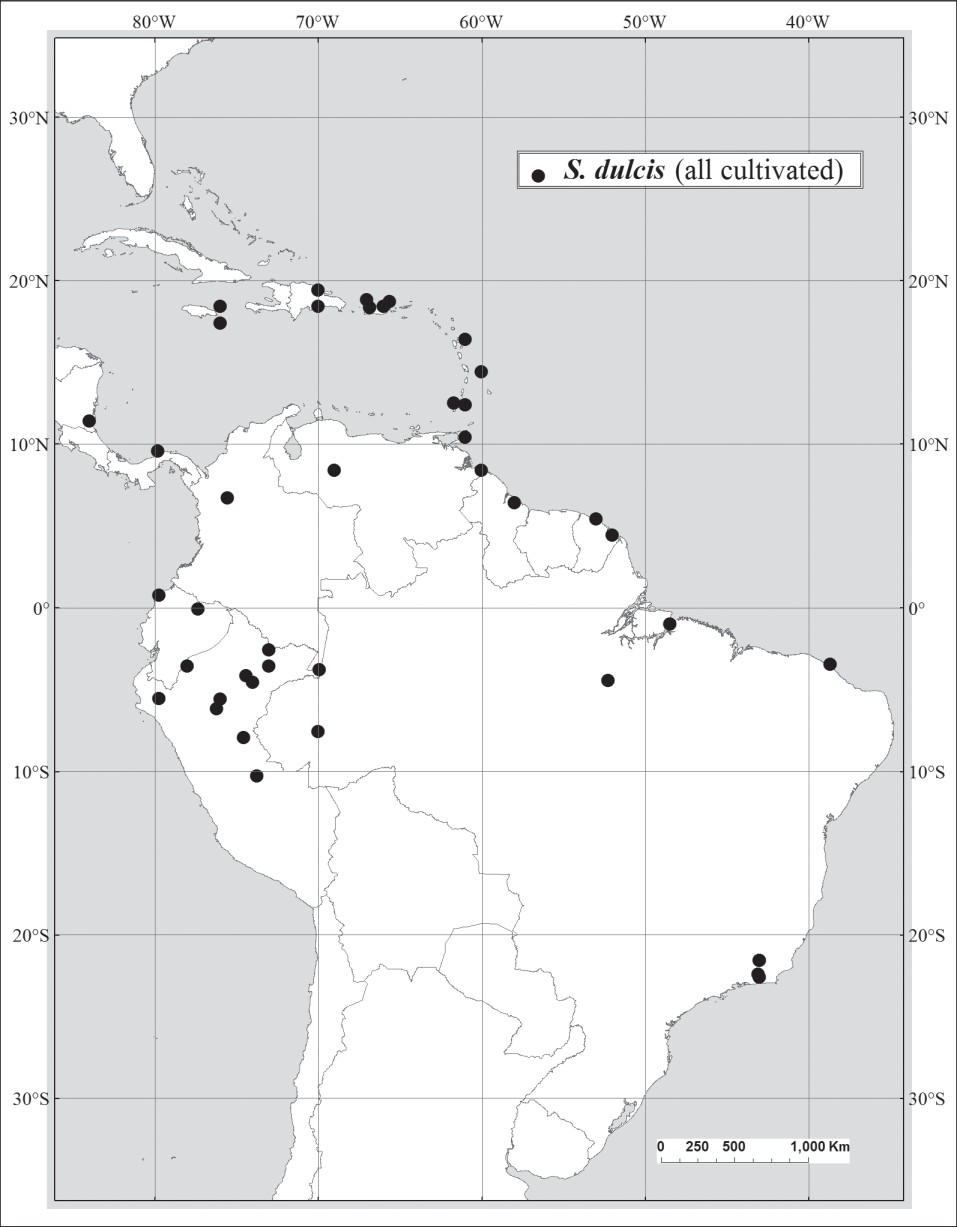
Distribution of *Spondias dulcis*.

Figs 2 , 15 , 16 , 20

Poupartia dulcis (Parkinson) Blume, Bijdr. fl. Ned. Ind. 1161. 1826–27. *Evia dulcis* (Parkinson) Blume, Mus. Bot. 1(15): 233. 1850. Type. based on *Spondias dulcis* Parkinson.
Spondias cytherea Sonn., Voy. Indes orient. 3: 242, t. 123. 1782.
Spondias dulcis Type. Mauritius (cultivated), *Commerson s.n.* (P!).
Spondias dulcis Parkinson var. commersonii Engl. in A. DC & C. DC., Monogr. phan. 4: 247. 1883.
Spondias dulcis Type. Several syntypes cited.
Spondias dulcis Parkinson var. mucroserrata Engl. in A. DC. & C. DC., Monogr. phan. 4: 247. 1883.
Spondias dulcis Type. Mexico, w/o date, Pavón 744 (G n.v.; GH-photo!, NY-photo!).
Spondias dulcis Parkinson var. integra Engl. in A. DC. & C. DC., Monogr. phan. 4: 248. 1883.
Spondias dulcis Type. Indonesia. Amboin, w/o date, Reinwardt s.n. (W!).

#### Type.

TAHITI. (without date), Capt. Cook [Banks & Solander] s.n. (lectotype, BM-793299 n.v., designated by A. C. Smith 1985: 453).

#### Description.

*Hermaphroditic, trees*, reproductive height 8–25 m. Trunk 20–40 cm diam.; *outer bark* light gray or light brown, thin, smooth to moderately rough, lenticellate, shed in small thin plates. Plant entirely glabrous except for some capitate glandular hairs. *Leaves* sometimes partially deciduous, 4–12-jugate, 11–60 cm long; petiole 9–15 cm long; lateral petiolules 2–8 mm long, the terminal one 10–30 mm long; basal leaflets 4.3–7.5 × 1.3–3.5 cm, other laterals 5–15 × 1.7–5 cm, all laterals oblong or lanceolate to ovate, terminal leaflet 5–9 × 1.9–3.5 cm, (narrowly) elliptic with acute base; leaflet apex acuminate or occasionally acute, the acumen 4–13 mm long, apex tip acute and glandular-mucronate; lateral leaflets medially subsymmetrical, basal width subsymmetrical, base insertion symmetrical and cuneate or obtuse, decurrent; margin slightly revolute and usually serrulate or crenulate, when present teeth concave-convex, sinus spacing regular, sinus glandular; leaflets chartaceous, adaxial surface sometimes glossy. *Inflorescences* usually developing with new leaf flush, terminal and axillary, congested at branchlet apex, 9–32.5 cm long, 3–7 mm diam at base; secondary axes to 11.5 cm long; bracts 0.4–5 mm long, linear to lanceolate, bracteoles 0.3–0.9 mm, linear to ovate; pedicel 1–3 mm long, portion distal to articulation 1–2 mm, sometimes the upper bracts and bracteoles and pedicel with scattered capitate glandular hairs. *Calyx* 0.7–1.2 mm long, aestivation apert, divided nearly to base, the lobes 0.5–1 mm long, deltate; petals 2–3 × 0.5–1.1 (1.3) mm, oblong to ovate or deltate, apex acute to slightly acuminate, cream-colored or white or whitish green, glabrous, reflexed at anthesis; stamens spreading, antesepalous and antepetalous ones 1.7–2.1 and 1.3–1.5(1.9) mm long, respectively, the anthers 0.7–0.8 mm long, in dorsiventral view elliptic to ovate, in lateral view oblong; disk 0.3–0.5(0.7) mm tall, 0.2–0.4 mm thick, summit undulate and outer margin sulcate, yellow; pistil ca. 1.3 mm long, depressed-ovoid to subcylindrical overall, divided most of its length into very thickly subulate, apically connivent styles ca. 0.8 mm long, the stigmas obovate, slightly extrorse. *Fruit* 4–10 × 3–8 cm when dry, ellipsoid, obovoid or oblong, maturing yellow or orange, base of fruit basicrescent over distal portion of pedicel, the endocarp lacking a fibrous matrix but provided with spiny projections extending into the mesocarp.

*Leaflet venation*: Fimbrial vein absent; secondary veins 12–20 pairs, usually darker than blade abaxially, usually straight and nearly perpendicular to midvein, spacing regular or sometimes decreasing toward base, angle increasing toward apex and base, insertion on midvein decurrent; intersecondaries ca. 1 per pair of secondaries and parallel to them, long and straight; intercostal tertiaries few, principally admedially branching parallel to secondaries but some irregular-reticulate, also some admedial tertiaries branching from intramarginal vein; quaternaries irregular-reticulate, FEVs highly branched, dendritic, tracheoid idioblasts absent; marginal secondary present; on abaxial side the midvein prominulous to prominent, secondaries flat; on adaxial side the midvein prominulous, secondary veins impressed to prominulous.

#### Distribution.

Broadly cultivated in lowland moist forest regions throughout the Neotropics.

#### Ecology.

Given this species’ broad distribution, its known phenology is broken down by region. Central America: fruiting Aug-Sep; West Indies: flowering Mar-May, fruiting Nov-Jul; NW South America W of the Andes: flowering and fruiting Dec; Amazonia: flowering Aug-Oct, fruiting Aug-Mar; extra-Amazonian Brazil: flowering Oct-Apr, fruiting Nov; Venezuela: flowering Apr; Guianas: flowering May.

There are reports in the literature that the fruits are dispersed by two species of large fruit-eating bats in the genus *Artibeus* (Lobova et al. 2009).

#### Common names.

Brazil, Rio de Janeiro: cajá manga (*Angeli 704*, NY); Dominican Republic: manzana de oro (*Zanoni & Mejía 16387*, NY); Ecuador, Napo: mauca (Yacu Indians, *Irvine 653*, F); Guadeloupe: pomme cythère (*Père Duss 3760*, NY, pro parte); Guyana: golden apple (*Omawale & Persaud 94*, NY); Jamaica: Jew plum (*Howard & Proctor 13531*, A)

Nicaragua, Río San Juan: jocote yuplón (*Sandino 3599*, NY); Panama, Panamá: mangoteen (*Miller & Merello 230*, NY); Peru, Loreto: tapiriba (*Martin & Plowman 1781*, ECON), San Martín: taperibá (*Scolnik 1193*, NY), kapiníwa (*Berlin 870*, NY); Puerto Rico: ambarella, jobo de la Índia (Little 14914, NY); Venezuela, Delta Amacuro: jobo de los indios (Wurdack 315, NY).

#### Economic botany.

*Spondias dulcis* (often referred to as *Spondias cytherea* in the literature and on herbarium specimens) has been in cultivation for so long that its native range in Asia is difficult to determine. It was introduced to Jamaica from the South Pacific in the 18^th^ Century (Popenoe 1948), and it is planted in home gardens throughout the humid neotropics. In the American tropics, the only significant use of the species is for its juice and as a flavoring for ice creams and sorbets, although it is used to flavor yogurts in the Caribbean; it reportedly has a high Vitamin C content (Lim 2012).

#### Selected specimens examined.

**BELIZE.** Toledo District, Temash River, ca. 11 km W of Caribbean Sea and ca. 3.5 km N of Belize/Guatemala border, ca. 15.949536°N, 89.033408°W, elev. 1 m, 8 Jun 1996, Atha & Romero 1372 (NY). **BRAZIL. Acre**: Mun. Tarauacá, Tarauacá town, 8.2°S, 70.8°W, 25 Sep 1994, Daly et al. 8361 (NY, HUFAC); **Rio de Janeiro**: Parque da Cidade de Gâvea, 10 Aug 1986, C. Angeli 704 (GUA, NY). **COLOMBIA**. **Amazonas**: Leticia, 19 Sep. 1966, ForeroGonzález 582 (NY). **DOMINICAN REPUBLIC**. **Prov. Cristóbal**: at CESDA property, just outside of city of San Cristóbal, 27 Jul 1981, Zanoni 15549 (NY). **ECUADOR**. **Esmeraldas**: Quinindé, Bilsa Biological Station, Mache Mountains, 35 km W of Quinindé, 5 km W of Santa Isabel, 2 lotes north of reserve, 400–600 m, 0°21'N, 79°44'W, 7 Dec. 1994, J. Clark 372 (NY); **Napo**: San José de Payamino, 40 km W of Coca, 0°30'S, 77°20'W, elev. 300-600 m, 20 Jan 1984, Irvine 653 (F). **FRENCH GUIANA**: Commune de Rémire, Île de Cayenne, 4°52S, 52°16'W, 25 Jul 1992 Wittingthon 44 (NY). **GRENADA**: St. George, Annandale Falls, 12°05'N, 61°43'W, 11 June 2001, Hawthorne et al. 459 (FHO, NY). **GUADELOUPE**: Grande-Terre, Grands-Fonds, Sainte-Anne, 12 Jul 1982, Barrier 3712 (NY). **GUYANA**: Diamond, east bank of Demerara River, 28 May 1970, Omawale & Persaud 94 (NY). **JAMAICA**. **St. Anne Parish**: grounds of Windsor Hotel (cult.), near St. Anne’s Bay, 20–31 Dec 1953, Howard & Proctor 13531 (A). **NICARAGUA**. Río San Juan, San Carlos, house S of cemetery (cult.), 16 Sep 1982, Sandino 3599 (NY). **PANAMA**. **Panamá**: Barro Colorado Monument, Frijoles train stop, 9°10'28"N, 79°47'48"W, elev. 37 m, 25 Aug 2001, Miller & Merello 230 (NY). **PERU**. **Amazonas**: Huampami, Rio Cenepa, village, elev. 800 ft., 10 Feb. 1973, B. Berlin 870 (NY). **PUERTO RICO**: Mun. Isabela, Arenales Altos, along Hwy. 112, 3.4 miles NE of junction with Hwy. 444, 18°25'30"N, 67°02'W, 8 Nov 1993, Nee 44157 (NY). **TRINIDAD**: Campus, University of the West Indies, 28 Jun 1916, Nevling 289 (A). **VENEZUELA**. **Delta Amacuro**: Río Grande between Curiapo and Pta. Cangrejo, 10 Apr 1955, Wurdack 315 (NY).

#### Conservation status.

Considering that this taxon is native to Asia/Oceania and rather widely cultivated in tropical America, it can be considered of Least Concern, at least for the Neotropics.

#### Discussion.

According to Smith (1985), the type specimen was made from a plant cultivated in Mauritius but grown from seed brought by Commerson from Tahiti in 1768. The earliest effective publication of *Spondias dulcis* is by Parkinson (J.voy. South Seas, 1773), a botanical artist who accompanied Banks and Solander in Captain Cooks’s first expedition to the Southern oceans. A later publication of this name by G. Forster (Pl. esc. 33. 1786) is considered an isonym as it is based on the same type as Parkinson’s name.

*Spondias cytherea* Sonn. was once considered the earliest valid name of this species, on the basis that the names in Parkinson’s publication were considered invalidly published (Airy Shaw and Forman 1967). A more recent examination of Parkinson’s work has shown *Spondias dulcis* to be a valid name and therefore to have priority over *Spondias cytherea* (Fosberg 1960). Following this argument, Smith (1985) lectotypified *Spondias dulcis* with a collection considered the voucher of Parkinson’s illustration of this species (and therefore a typotype).

In 1869, Marchand subsumed *Spondias acida*, *Spondias amara*, and *Spondias pinnata* under *Spondias dulcis* as varieties. Here *Spondias acida* and *Spondias pinnata* are maintained as species, and *Spondias amara* is considered a synonym of *S. pinnata. Spondias dulcis sensu* Blanco is attributable to *Spondias purpurea*.

### Spondias expeditionaria

Taxon classificationPlantaeSapindalesAnacardiaceae

J. D. Mitch. & Daly sp. nov.

urn:lsid:ipni.org:names:77148928-1

Figs 16 , 19 , 21

#### Diagnosis.

Rare, small to medium-sized moist forest tree 8–12 m tall, with densely and shallowly fissured bark; similar to *Spondias macrocarpa* Engl. because of the 7–12-jugate leaves (to 16 juga in *Spondias expeditionaria*), lanceolate leaflets, and long anthers (0.7–0.9 mm long), but S. expeditionaria has shorter pedicels (1.6–2.2 vs. 2.5–3.5 mm long), a taller disk (0.65–0.75 vs. (0.1) 0.3–0.6 mm tall), and the fruit (depressed-)globose vs. oblong to slightly (ob)ovoid.

#### Type.

BRAZIL. Espírito Santo: Mun. Aracruz, Barra do Riacho, on levee of Rio Guandú, km 22 of Baixo Guandú-Ibituba road, right side, 15 Dec 1991, D. A. Folli 1534 (holotype: CVRD!; isotypes: MO!, NY!).

#### Description.

*Hermaphroditic trees*, reproductive height 8–12 m. Trunk 36–77 cm diam.; *outer bark* brown, densely and shallowly fissured. *Resin* clear. *Trichomes* of three types: curved or flexuous or less often (sub)erect hairs to 0.4 (0.7) mm long; yellow to orange glandular hairs to 0.05 mm long; and (petiole and rachis only) fine bristles to 0.05 mm long. *Leaves* 8–16-jugate, 30–46 cm long; petiole 2.5–6.3 cm long; petiole, rachis and petiolules with dense erect to flexuous hairs, sometimes also with sparse to dense glandular hairs; lateral petiolules 0–2 mm long, the terminal one 3–27 mm long; basal leaflets 1.4–4.2 × 0.9–2.9 cm, ovate, other laterals 2.2–11 × 1.1–3.2 cm, (oblong-)(ob-)lanceolate, terminal leaflet 2.4–5 × 0.8–2.8 cm, (narrowly) elliptic; leaflet apex gradually and narrowly acuminate, the acumen 3–17 mm long; lateral lamina medially symmetrical, the base subsymmetrical or sometimes asymmetrical, slightly cordate or rarely truncate, base insertion excurrent; leaflet margin slightly revolute and sparsely and bluntly serrulate to crenate, the teeth convex-convex and the sinus shallow, the margin sometimes ciliate; leaflets membranaceous to chartaceous, both surfaces dull. *Inflorescences* terminal on leafy branches, 9.8–19 cm long, ca. 1.3 mm diam at base, broadly branched, the secondary axes 4.5–6.5 cm long, the axes glabrous or sometimes with sparse to scattered glandular hairs; bracts subtending inflorescences 1.5–1.6 mm long, subulate with acute apex, those subtending secondary axes 0.6–1.5 mm long, lanceolate and acuminate, some sparsely ciliate, bracteoles 0.4–0.7 mm long, ovate to lanceolate, acute to acuminate; pedicel 1.6–2.2 mm long, portion distal to articulation 1.2–1.6 mm long. *Calyx* 0.6 × 1.4–1.5 mm overall, aestivation apert, the lobes 0.35–0.4 mm long, triangular to lanceolate, somewhat fleshy; petals 1.8–2 × 1.1–1.2 mm, slightly obovate, apex acute to slightly acuminate, glabrous, the margin thickened, white or cream, sometimes with scattered glandular hairs, reflexed at anthesis; stamens spreading, the antesepalous and antepetalous ones 2.1–2.8 and 1.9–2.7 mm long, respectively, the anthers 0.7–0.9 mm long, in dorsiventral view broadly ovate, in lateral view oblong; disk 0.65–0.75 mm tall, 0.1–0.25 mm thick, summit craggy and outer margin crenellate; pistil ca. 1.5 mm long, subcylindrical overall, divided ca. 2/3 its length into subulate, apically slightly divergent styles 0.8–0.9 mm long, the stigmas extrorse, linear to lanceolate. *Fruits* 3–3.3 × 3–3.8 cm (fresh), (depressed-)globose, sometimes slightly rounded 5-costate, maturing yellowish, the surface glabrous, dull, with sparse, small, flat lenticels, the endocarp 2.9–3.2 mm diam, often 5-costate.

*Leaflet venation*: Fimbrial vein absent; secondary veins 7–14 pairs, spreading, the spacing and angle sometimes irregular, the angle increasing toward the base, insertion excurrent or abruptly decurrent; some perpendicular inter-secondaries present, also sometimes some perpendicular epimedial tertiaries present; intercostal tertiary veins irregular-reticulate with some admedial branching; areoles poorly developed, mostly at tertiary rank, FEVs 4+-branched, dendritic, terminating in tracheoid idioblasts; marginal ultimate venation incompletely looped; on abaxial side the midvein and secondary veins narrowly prominent, tertiaries prominulous and slightly darker than the lamina, the midvein with sparse trichomes, rest of blade with scattered trichomes of all three types; on adaxial side the midvein narrowly prominent, the secondary and tertiary veins prominulous to flat, the midvein and secondary veins with dense to sparse trichomes and sometimes scattered glandular hairs.

#### Distribution.

Mata Atlântica (Atlantic coastal forests) complex in the states of Minas Gerais and especially Espírito Santo, Brazil.

#### Ecology.

Three of the four known collections were made in pasture, a coffee plantation, and a secondary forest, so although it appears to be rare, the species is likely adapted to disturbed conditions. It has been collected in flower in Oct-Dec and in fruit in March.

#### Common name.

Cajá mirim (V. de Souza et al. 390, CVRD).

#### Etymology.

The specific epithet derives from the collecting locality of Expedicionário Alício in Minas Gerais, an appropriate name considering the obvious need for intensified botanical inventory in the region.

#### Specimens examined.

**BRAZIL. Espírito Santo**: Santa Teresa, Pedra Alegre, property of Domingos Demuner, 2 Mar 2003, Demuner 1583 (NY); Baixo Guandú, right side of Baixo Guandú-Mutum Preto road, 9 Nov 1992, Souza et al. 390 (CVRD, MO, NY). **Minas Gerais**: Mun. Expedicionário Alício, 16 Oct 1997, Lorenzi s.n. (NY, XLORNZI).

#### Conservation status.

We propose to classify this species as Critically Endangered, with the following justifications: (1) we are aware of only four herbarium collections from only two distinct localities; (2) it is evidently rare considering how few collections have been made; and (3) there is very little forest remaining in these collecting localities.

#### Discussion.

*Spondias expeditionaria* resembles *Spondias macrocarpa* because the leaves have numerous, usually lanceolate leaflets, excurrent leaflet base insertion, sometimes irregular spacing of secondary veins, lack of dense tufts of hairs at the leaflet bases abaxially, relatively long stamens and large anthers, the disk much taller than thick, and relatively large fruits, but the new species differs by having subsymmetric (vs. asymmetric) leaf base, the teeth convex-convex (vs. markedly concave-convex), intersecondaries (when present) perpendicular (vs. parallel); costal tertiaries irregular-reticulate with some admedial branching (vs. very little reticulation and admedially freely ramified), flower pedicel 1.6–2.2 (vs. 2.5–3.5) mm long, calyx lobes triangular (vs. ovate), petals obovate (vs. essentially elliptic), and fruit (depressed-)globose (vs. slightly ovoid to ellipsoid).

*Spondias expeditionaria* appears as *Spondias macrocarpa* in *Árvores Brasileiras* (Lorenzi 1998), *Brazilian Trees* (Lorenzi 2002), and *Brazilian Fruits and Cultivated Exotics* (Lorenzi et al. 2006), all published before discovery of the new species. The plates show habit, bark, wood, a flowering branchlet, and mature fruits and endocarps.

### Spondias globosa

Taxon classificationPlantaeSapindalesAnacardiaceae

J. D. Mitch. & Daly sp. nov.

urn:lsid:ipni.org:names:77148929-1

Figs 1 , 2 , 10 , 13 , 15 , 16

#### Diagnosis.

Canopy or emergent tree to 40 m tall, inner bark red with narrow white striations; similar to *Spondias mombin* because of the similar indumentum, the inflorescences highly branched, disk short and thick, and fruits of similar size; *Spondias globosa* differs by the outer bark lacking spinose projections (vs. corky, tubercular, or spinose projections), intersecondary veins parallel to secondaries and strong, often reaching intramarginal vein (vs. intersecondaries reticulating and weak, not usually reaching intramarginal vein), intercostal tertiaries arising at or near intramarginal vein (vs. intercostal tertiaries primarily irregular-reticulate), fruits globose to perdepressed-ovoid, rarely very slightly oblong of obovoid (vs. oblong or less often ellipsoid or slightly oblong-ovoid).

#### Type.

BRAZIL. Acre: Mun. Santa Rosa, Alto Rio Purus, left bank, Seringal Mamuriá, ca. 9°05'05"S, 69°59'07"W, 25 Mar 1999, D. C. Daly, H. Kuchmeister, D. Gomes da Silva, L. Lima & E. Consuelo 10039 (holotype: HUFAC!; isotypes: AAU!, MO!, NY!).

#### Description.

*Hermaphroditic trees*, reproductive height 8–40 m. Trunk 10–105 cm diam; *outer bark* (light) gray to brown, usually thin, usually with many long, broad, shallow, wavy fissures, sometimes rough but lacking spinose projections, also with small white lenticels, shed in flat, narrow, regular plates; *inner bark* red (less often orange) with narrow white (less often beige) striations, or red-and-white striate, thick. *Trichomes* of two types: straight or slightly curved erect white hairs to 0.15 mm long; and short, usually straight, erect, whitish bristles to 0.05 mm long. *Leaves* (1) 3–6 (7)-jugate,13–40 cm long; petiole 3.8–8 cm long, petiole and rachis glabrous or with sparse bristles, flanks of petiolules with dense longer hairs and sparse bristles; lateral petiolules 3–10 mm long, the terminal ones 12–32 mm long, petiolules with slightly curved white hairs; the basal leaflets 3.2–8.5 × 1.8–4.3 cm, (broadly) elliptic to (broadly) ovate, sometimes broadly obovate or almost rotund, other laterals (3.2) 6.2–11.5 (14.5) × 2–4.9 (5.3) cm, sometimes slightly obliquely ovate or lanceolate but more often strongly asymmetrical and the acroscopic side semi-ovate to semi-lanceolate and the basiscopic side semi-(oblong-)elliptic, sometimes broadly so; terminal leaflet 3.7–9.5 × 1.8–5 cm, (broadly) elliptic, obovate, or oblanceolate; leaflet apex usually abruptly and narrowly long-acuminate or sometimes broadly short-acuminate, the acumen (3) 6–18 cm long, often the apex tip mucronate; lateral lamina medially and basally slightly to strongly asymmetrical, the acroscopic side truncate to rounded or rarely obtuse, the basiscopic side cuneate to attenuate; basal insertion often asymmetrical, both sides abruptly decurrent; margin entire, sometimes slightly revolute, sparsely ciliate with longer hairs; leaflets chartaceous to subcoriaceous, sometimes glossy adaxially. *Inflorescences* (sub)terminal, produced with leaf flush, 15–26 cm long, 3–5 mm diam near base, broadly branched, secondary axes 1.8–14.5 cm long, these axes with dense to sparse bristles, higher-order axes with sparse bristles, also with sparse longer hairs; bracts on primary axes ca. 4 mm long, lanceolate, those on secondary axes 0.6–1.6 mm long, lanceolate to deltate, with dense bristles on both surfaces, bracteoles 0.25–0.5 mm long; pedicel 1.2–3 mm long overall, portion distal to articulation 0.5–1.3 mm long, pedicel and both sides of calyx with sparse bristles (denser toward base). *Calyx* 0.8–1 mm long overall, aestivation apert, the lobes 0.4–0.7 mm long, deltate to narrowly ovate, calyx with pubescence as on pedicel, the margin often ciliate; petals 2.4–3.3 × 1.2–1.5 mm, lanceolate, apex slightly acuminate, whitish to yellowish or cream, abaxial surface glabrous, reflexed at anthesis; stamens spreading, the antesepalous and antepetalous ones 2–2.3 and 1.4–1.6 mm long, respectively, the anthers 0.8–1.1 mm long, in dorsiventral view oblong, in lateral view oblong to elliptic; disk 0.3–0.5 mm tall, 0.2–0.3 mm thick, summit markedly undulate and outer margin deeply sulcate; pistil (1) 1.5–1.8 mm long, depressed-ovoid overall, divided ca. 2/3 its length into broadly subulate, apically slightly divergent styles 0.8–1.2 mm long, extrorse, stigmas vertically elliptic. *Fruits* 1.6–3 × 2.2–3 cm diam (to 4 cm diam fresh), usually (depressed-) globose, rarely very slightly oblong or obovoid (then the apex obtuse), maturing yellow, surface smooth, dull. *Seedlings* (Pennington et al. 17244, NY): cotyledons 2.1–2.4 cm long, with several parallel veins; first eophylls opposite, trifoliolate, petiolules with sparse curved hairs, the leaflets ovate, margin glabrous, sparsely toothed, the teeth concave-convex.

*Leaflet venation*: Fimbrial vein absent; secondary veins in 9–15 pairs, straight to slightly arcuate, the spacing decreasing toward apex and base, the angle decreasing toward the apex and increasing toward base, insertion on midvein decurrent; intersecondaries occasional, parallel to secondaries and almost reaching the intramarginal vein; intercostal tertiaries few, most of them arising from near the intramarginal vein and forming strong composite admedials parallel to secondaries, with some irregular reticulation; quaternaries irregular-reticulate and freely admedially ramified; areolation usually at quaternary rank, FEVs 5+- branched, dendritic, tracheoid idioblasts absent; marginal ultimate venation usually looped (sometimes incompletely); on abaxial side all veins narrowly prominent or sometimes the secondaries and tertaries prominulous, occasionally discolorous; on adaxial side all veins narrowly prominulous to almost flat or occasionally all but the midvein slightly impressed; on both surfaces the midvein with scattered longer hairs and bristles near the base and (sub)glabrous distally, sometimes glabrescent.

#### Distribution.

*Spondias globosa* is a western Amazon element, apparently disjunct to Zulia and Barinas in western Venezuela.

#### Ecology.

This is very much a lowland taxon, ranging only between 100–350(500) m elevation. It is most often found in formations such as floodplain forests or *tahuampa* forest on poorly drained, periodically or seasonally inundated soils, although it has been reported from a range of soils including not just black alluvial soils but also oxisols, lateritic soils, and red and yellow clay soils. Apart from flooded formations it is found in primary forests in well-drained soils, including on undulating or hilly terrain. Occasionally it grows in secondary forest, bamboo-dominated forest, or rarely pasture or shrubby disturbed vegetation.

In SW Amazonia, this species is known to flower in Sep-Nov and fruit Oct-May, but in NW Amazonia the collection data indicate that it can be found flowering and fruiting all year.

The yellow-footed tortoise, Geochelone (Chelonoidis) denticulata, has been observed dispersing the fruits of *Spondias globosa* (as *Spondias venulosa*) in Amazonian Peru and Colombia (Rodríguez-Bayona and Rylander 1985 and Stevenson et al. 2007, respectively). These fruits comprise an important food source in times of scarcity for primates such as the woolly monkey (Stevenson 2005).

#### Common names.

Brazil, Acre: cajá (Cid Ferreira & Nelson 3066, NY), taperibá (Daly et al. 10039, NY), taperebá (Silveira et al. 1622, NY); Venezuela: jobo (Steyermark 102015, NY); Ecuador: aurumuyo (Quichua, Zuleta 191, NY) azua muyo (Quichua, Moya & Reyes 146, NY); mientuhue (mientuhuem for fruit)(Huaorani, Miller et al. 703, NY); mamantunim (Shuar, Jua (RBAE) 69, NY); mïyëtowëmo (Wao, Ríos 576, NY); mientohuemo (Huaorani, Aulestia & Gonti 1769, NY); mientuhueno (Aulestia et al. 3020, NY); ovu muyo (Huaorani, Aulestia et al. 402, NY); mijentuemo (Huaorani, Dik & Andi 906, NY); Peru, Loreto: ubos (Martin & Lau-Cam 1252, ECON), huvos (Torres 88, GH), hubus (Schunke 250, A), hubos (Torres 350, ECON), ubos colorado (Chota 5, NY).

#### Economic botany.

Fruit edible (Schunke 250, A); bark cooked with water taken for diarrhea (Plowman et al. 7257, ECON); fruit pulp used to make a fruit juice (Daly et al. 10039, NY); for chronic diarrhea, make tea from 1 kg of finely chopped bark and drink twice daily, or use liquid concoction as vaginal douche to treat *flor blanca* (‘yeast infections?’), or apply to infected wounds (Chota 5, NY); fruits are edible, much appreciated and frequently sold in Iquitos market (Peters & Hammond 164, NY); branches and trunk used as firewood (Jua (RBAE) 69, NY); eaten by a number of animals (Miller et al. 703, NY), eaten by game animals (Lizarralde ML307, NY). In Acre, Brazil, Kainer and Duryea (1994) observed preparation of a type of *tucupi* sauce that combines hot peppers with the juice of *Spondias globosa* fruits.

#### Etymology.

The specific epithet refers to the usually globose fruits characteristic of this taxon.

#### Selected specimens examined.

**BOLIVIA**. **Beni**: Prov. Ballivián, Estación Biológica Beni, 56 km E of Río Maniqui on road to Trinidad, then 18 km NNE to Estancia 07, then 6 hrs to Río Maniquicito, 250 m, 14°44'S, 66°20'W, 6 Nov 1985, Solomon 14593 (NY); **Pando**: Manuripi, vicinity of La Conquista, elev. 160 m, 19L FH95, 30 Jan 1983, Fernández-Casas & Susanna 8566 (MO, NY); **Santa Cruz**: Prov. Ichilo, E side of Río Tapacani at junction with Río Surutu, 0.5 km upstream and S from bridge over Río Yapacani at Villa Tapacani, 17°24'S, 63°50'W, 30 Oct 1990, M. Nee 39607 (MO, NY, TEX). **BRAZIL. Acre**: Mun. Sena Madureira, basin of Rio Purus, Rio Iaco, right bank, Nova Olinda, between Igarapé Santo Antônio and Ig. Boa Esperança, 10°07'S, 69°13'W, 22 Oct 1993, Daly et al. 7836 (HUFAC, NY, TEX); **Amazonas** [erroneously sited in Acre state on label]: Mun. Boca do Acre trail from W bank of Rio Iaco to Rio Purus, 3 km above confluence, 5 Oct 1968, Prance et al. 7873 (GH, MG, NY, R). **COLOMBIA**. **Amazonas**: Aduche, Asentamiento Muinane, south bank of río Caquetá, 0°41'30"S, 72°06'00"W, 11 May 1999, Arévalo & Reyes 57 (NY); **Meta**: Parque Nacional Natural Tinigua, Serranía Chamusa, Centro de Investigaciones Primatológicas La Macarena, 7 Mar 1990, Stevenson 109 (COL). **ECUADOR**. **Morona**-**Santiago**: Centro Shuar-Yukutais, 3°30'S, 78°10"W, 18 Apr 1989, Bennett & Gómez A. 3711 (NY); **Napo**: Orellana, Parque nacional Yasuní, km 46-52 Maxus road under construction, elev. 250 m, 00°47'S, 76°30'W, 1–11 Sep 1993, Aulestia et al. 402 (NY); **Pastaza**: “Moretecocha” oil well of ARCO, río Landayacu, 75 km E of Puyo, elev. 580 m, 1°34'S, 77°25'W, 4 Dec 1990, Gudiño 1158 (NY); **Sucumbios**: Cuyabeno, Parroquia Tarapoa, Siona community of Sototsiaya, 50 min. downstream from Poza Honda on Río Aguarico, 00°14'27"S, 76°26'15"W, elev. 230 m, 25 Feb 2005, Miranda & Moya 446 (MO). **PERU**. **Amazonas**: Río Santiago, behind community of Caterpiza, elev. 200 m, 4 Sep 1979, Huashikat 392 (NY); **Huánuco**: Prov. Puerto Inca, Dtto. Yuyapichis, Unidad Modelo de Manejo y Producción Forestal Dantas, 9°40'S, 75°02'W, 16–30 Nov 1989, Kröll 694 (NY); **Loreto**: Río Nanay, Puerto Almendras, ca. 20 km WSW of Iquitos, ca. 3°46'S, 73°20'W, 15 Mar 1989, Chota 5 (NY); **Madre de Dios**: Prov. Tambopata, Zona Reservada Tambopata-Candamo, along trails of Explorer’s Inn, 12°49'S 69°18'W, 22 Apr 1991, Phillips & Chávez 636 (NY); **Ucayali**: Prov. Coronel Portillo, Carretera Marginal, 22 km S of km 86 on PucallpaTingo Maria Highway, 75°00'W, 8°41'S, 11 Feb. 1981, Gentry et al. 31215 (NY). **VENEZUELA**. **Barinas**: Reserva Forestal Caparo, 16–18 km SE of Campamento Cachicamo, E of El Cantón, elev. 100 m, 9 Apr 1968, Steyermark et al. 102015 (NY); **Zulia**: along Quebrada Perayra, tributary of Río Tokuku (Tocucu), SW of Misión de Los Angeles de Tokuku, SW of Machiques, 29 Aug 1967, Steyermark 99828 (NY).

#### Conservation status.

This taxon is widespread in Amazonia and can be considered of Least Concern except in Zulia, Venezuela (Maracaibo watershed), where very little lowland forest remains and where it has been collected only once.

#### Discussion.

Although *Spondias globosa* is geographically sympatric with *Spondias mombin* in many localities,the two appear to have undergone niche partitioning: in interviews with forest residents in the middle Ucayali and upper Purus rivers, they readily recognized the two as distinct taxa long before botanists came to the same conclusion, pointing out not only differences in the bark and fruits but also that *Spondias globosa* tends to keep to the floodplains (vs. terra firme) and flowers and fruits later in any given locality. In the middle Ucayali the prevailing common name for *Spondias globosa* is “uvos colorado,” referring to its mostly red (versus usually pale pink) inner bark.

Morphologically, the two can be distinguished by *Spondias globosa* lacking corky tubercular or spinose projections, the inner bark usually (pale) red-and-white striate (vs. inner bark pale red to pink to orange, sometimes striate with beige), the leaves 3–5 (–7)-jugate (vs. 3–7 (–12)-jugate), the leaflets with composite admedial tertiaries arising at or near the intramarginal vein (vs. tertiary veins primarily irregular-reticulate, some admedial branching), fimbrial vein absent and the marginal ultimate venation incompletely looped (vs. fimbrial vein present), the flower pedicel 1.2–3 mm long (vs. 2–4.5 mm long), the fruit usually (depressed-)globose, rarely very slightly oblong or obovoid (vs. oblong or less often ellipsoid or slightly oblong-ovoid), and occurring in W Amazonia plus Zulia and Barinas in Venezuela (vs. central Mexico S to SE Brazil and widely cultivated in the moist tropics).

Table 4 summarizes the morphological characters that separate the two species rather consistently. The existence of possible hybrids between *Spondias mombin* and *Spondias globosa* is discussed in the section on Hybridization and Intermediates in the Introduction; some examples are Grández & Jaramillo 2042 (MO, NY), Spichiger & Encarnación 1095 (MO), and Vásquez et al. 4873 (MO, NY).

**Table 4.** Comparison of *Spondias mombin* and *Spondias globosa*.

Character	*Spondias mombin*	*Spondias globosa*
Outer bark	Often with corky tubercular or spinose projections in sunny growing conditions	Sometimes rough but lacking spinose projections
Inner bark	Pale red to pink to orange, sometimes striate with beige	Red with white (less often beige) striations, or red-and-white striate
No. of juga	3–7 (–12)	3–5 (–7)
Intersecondary veins	Weak, reticulating (not Reaching intramarginal vein)	Strong, parallel, reaching Intramarginal vein
Intercostal tertiary veins	Primarily irregular-reticulate, some admedial branching	Composite admedial tertiaries arising at or near the intramarginal vein
Quaternary veins	Predominantly irregular-reticulate, some freely ramifying	Predominantly freely ramifying, some irregular-reticulate
Fimbrial vein	Present	Absent
Marginal ultimate veins	Marginal FEVs between fimbrial and intramarginal veins	Usually looped
Fruit shape	Oblong or less often ellipsoid or slightly oblong-ovoid	Globose to perdepressed-ovoid; rarely very slightly oblong or obovoid
Geography	Native to S Mexico S to Paraguay, possibly native to E Brazil, widely cultivated in the moist tropics	W Amazonia plus Zulia and Barinas in Venezuela

### Spondias macrocarpa

Taxon classificationPlantaeSapindalesAnacardiaceae

Engl. in Mart., Fl. bras. 12(2): 375, pl. 78. 1876.

Figs 2 , 15 , 16 , 19

Spondias dulcis Parkinson var. macrocarpa (Engl.) Engl. in A. DC. & C. DC., Monogr. phan. 4: 247. 1883. Type. Based on *Spondias macrocarpa* Engl.

#### Type.

BRAZIL. Rio de Janeiro: Canta Gallo [Cantagalo], 1859, Peckolt 224 (Lectotype: BR-572018, here designated).

#### Description.

*Hermaphroditic trees*, reproductive height 12–22 m. Trunk 24–60 cm diam; *outer bark* brown, rough, thin, scaly, shed in usually large irregular plates; *inner bark* red with tan striations. *Trichomes* of three types: long, white, flexuous to nearly straight, sometimes uncinate, 0.7–1 mm long on vegetative parts; short, erect hairs usually less than 0.1 mm long, primarily on basal portions of inflorescence; and orange capitate glandular hairs to 0.05 mm long (these rare). *Leaves* 7–12jugate, 12–30 cm long; petiole 3–5 cm long, petiole and rachis with dense long white hairs; lateral petiolules 1–3 mm long, the terminal one 3–20 mm long, petiolules with hairs as on rachis; basal leaflets 1.7–4.8 × 0.7–1.8 cm, ovate or less often lanceolate, other laterals 5–7 × 1–2 cm, (falcate)lanceolate to elliptic, terminal leaflet 2–6 × 0.7–1 cm, ovate to lanceolate; leaflet apex long-acuminate, the acumen 4–13(20) mm long; lateral lamina medially and basally asymmetrical, acroscopic side obtuse to cordate, basiscopic side attenuate to cuneate, basal insertion on petiolule (sub)symmetrical and excurrent or slightly decurrent; margin flat to slightly revolute (often slightly so at base) and sparsely serrulate, the teeth usually concave-convex, sinus appearing glandular; leaflets membranaceous to chartaceous, both surfaces dull. *Inflorescences* (sub)terminal, developing with leaf flush, 5–15 cm long, ca. 4 mm diam at base, secondary axes to 2 cm long, axes with sparse to dense short erect hairs toward base, distal portions glabrescent, bracts on inflorescence axes to 0.5–1.2 mm long, bracteoles 0.3–0.5 mm long, all bracts ovate to subulate; pedicel 2.5–3.5 mm long overall, portion distal to articulation 0.7–2.7 mm. *Calyx* 0.5–0.6 mm long overall, aestivation apert, divided nearly to base, the lobes 0.3–0.5 mm long, (depressed-)deltate, margin occasionally papillate; petals 2.5–2.6 × 1.3–1.6 mm, essentially elliptic, acute or usually slightly acuminate, white or cream, glabrous, reflexed at anthesis; stamens spreading, the antesepalous and antepetalous ones 2.9–3.0 and 2.5–2.6 mm long, respectively, the anthers 0.7–0.9 mm long, oblong in both dorsiventral and lateral views; disk (0.1) 0.3–0.6 mm tall, 0.1 mm thick, summit shallowly undulate and outer margin nearly entire; pistil 1.5 mm overall, depressed-ovoid to subcylindrical overall, divided halfway to 2/3 its length into 5 broadly subulate, apically connivent styles 0.8–1 mm long, stigmas extrorse, obovate. *Fruits* 3.5–4.2 × 2.3–2.5 cm (dry), maturing yellowishgreen to (orange-)yellow, oblong to slightly (ob)ovoid, surface sparsely whitishlenticellate, mesocarp whitish, sweetsour.

*Leaflet venation*: Fimbrial vein absent; secondary veins 10–15 pairs, straight to slightly arcuate, insertion on midvein excurrent or abruptly decurrent, spacing irregular, angle usually irregular, slightly acute to nearly perpendicular; intersecondaries and/or epimedial tertiaries sometimes present, parallel to secondaries, nearly reaching intramarginal vein, branching admedially; intercostal tertiaries few per secondary vein, strongly admedially branched, sometimes also sparsely branched toward margin; quaternary veins irregular-reticulate and freely ramified; areolation at tertiary and quaternary ranks; FEVs 3+-branched, dendritic, terminating in tracheoid idioblasts; marginal ultimate venation incompletely looped; on abaxial side all veins narrowly prominent, on adaxial side the midvein narrowly prominulous (almost keeled) but sunk in a groove, rest of veins flat to impressed and obscure; both sides sparsely to densely pubescent.

#### Distribution.

*Spondias macrocarpa* is native to moist upland forests of the Mata Atlântica Complex, in southern Bahia, Rio de Janeiro, Espírito Santo, and extreme southeastern Minas Gerais.

#### Ecology.

This species appears to be rare where it does occur. It has been recorded in *mussununga* forest (dense forest with discontinuous canopy 8–15 m high, on level terrain, in sandy soils (spodosols) that are often poorly drained)(Stefano et al. 200, NY), and *tabuleiro* forest (dense forest with continuous canopy 20–25 m high, on level terrain, in sandy clay soils (oxisols))(Stefano et al. 225, NY). The species is known to flower Jun–Feb and to fruit Mar–Apr (Jun).

#### Common names.

Brazil. Espírito Santo: cajá mirim (Farias 475, NY); Rio de Janeiro: acajá (Peckolt s.n. (BR-571916, BR), cajá (Peckolt 224, BR).

#### Selected specimens examined.

**BRAZIL**. **Bahia**: São Paulinho, on road to Catolesinho, 9 Nov 1942, Fróes 12671/37 (A, NY); by Itatinga road, [15°15'S, 40°15'W] 7 Oct 1945, Fróes 20079 (IAN, NY); Mun. Santa Cruz Cabrália, Estação Ecológica do Pau Brasil-ESPAB, ca. 16 km W of Porto Seguro, BR-367 (Porto Seguro-Eunápolis) highway, 18 Dec 1987, F. Souza Santos 820 (CEPEC); Mun. Juçari, Fazenda Sto. Antônio Alciato de Carvalho, ca. 6 km N of Juçari, 0.5 km from “Fazenda de Cacau,” 22 Jun 1991, Thomas et al. 6823 (CEPEC, NY); **Espírito Santo**: Linhares, Reserva Natural Vale, next to native plant nursery, 26 Nov 1991, Farias 475 (CVRD, NY); Mun. Linhares, Reserva Natural Vale, 1700 m on Farinha Seca Road, 27 Apr 1992, Folli 1614 (CVRD, NY); Mun. Linhares, Reserva Florestal da Sooretama, 10 Feb 1993, Hatschbach & Silva 60062 (TEX); Mun. Pinheiros, Pinheiros, km 12 of Pinheiro-Montanha road, 50 m from asphalt, 23 Nov 1991, V. Souza 267 (CVRD, NY); Reserva Florestal da CVRD, Linhares, 11 Nov 1977, J. Spada 013/77 (CVRD, NY); Mun. Linhares, Reserva Vale (BR-101 Norte, km 122), Estrada Flamengo, 19°7'14"N, 39°54'59"W, 1 Mar 2011, Stefano et al. 200 (CVRD, NY, RB); **Minas Gerais**: Alegria, near Caraça, 12 Oct 1882 (1883 on BR sheet), Glaziou 13678 (BR [on-line image seen], K, P, R); Serra da Carajá, 12 Aug 1882, Glaziou 13679 (P); Coronel Pacheco, Estação Experimental do Café, 6 Sep 1940, Heringer 60 (RB); **Rio de Janeiro**: Canta Gallo [Cantagalo], 1859, Peckolt 224 (BR 571985) (BR), Jan 1860, Peckolt s.n. (BR 571916)(BR), 1860, Peckolt s.n. (BR 571982)(BR).

#### Conservation status.

We classify this species as “Vulnerable,” although it is relatively widespread in Atlantic Forest Complex of Brazil, because of the relatively small number of known specimens represented in a well-collected region. It should be noted that many of the collections are rather old and may be from localities that are no longer forested.

#### Discussion.

The species referred to as *Spondias macrocarpa* in *Árvores Brasileiras* (Lorenzi 1998), *Brazilian Trees* (Lorenzi 2002), and *Brazilian Fruits and Cultivated Exotics* (Lorenzi et al. 2006) is in fact *Spondias expeditionaria*; these books were published before discovery of the latter. Indeed, *Spondias macrocarpa* most closely resembles *Spondias expeditionaria*; a comparison of the two appears under the latter species.

### Spondias mombin

Taxon classificationPlantaeSapindalesAnacardiaceae

L., Sp. pl. 371. 1753.

Figs 2 , 7 , 9 , 11 , 12

Spondias myrobalanus L., Syst. nat. ed. 10, 2: 1036. 1759 (non L., Fl. jamaic. 1759), nom. illegit. Type. Based on *Spondias mombin* L.
Spondias lutea L., Sp. pl. ed. 2, 1: 613. 1762, nom. illegit.; *Spondias lucida* Salisb., Prodr. stirp. Chap. Allerton 172: 1796, nom. illegit. Type. Based on *Spondias mombin* L.
Spondias cirouella sensu Tussac, Fl. Antill. 3: 37, t. 8. 1824, excl. synonyms of *Spondias purpurea* L.
Spondias pseudomyrobalanus Tussac, Fl. Antill. 4: 97, t. 33, 1827. Spondias lutea L. var. pseudomyrobalanus (Tussac) Marchand, Rév. Anacardiac.: 156. 1869.
Spondias mombin Type [icon]: Tussac, Fl. Antill. 4: t. 33. 1827 (lectotype, here designated).
Spondias graveolens Macfadyen, Fl. jamaica 1: 228. 1837.
Spondias mombin Type. Ghana, Thonning s.n. (C n.v.).
Spondias lutea L. var. glabra , Engl. in Mart., Fl. bras. 12(2): 374. 1876.
Spondias mombin Type. Brazil. Minas Gerais: Contendas, w/o date, Martius s.n. (M, n.v.)
Spondias lutea L. var. maxima Engl. in Mart., Fl. bras. 12(2): 374. 1876.
Spondias mombin Type. Haiti, pro pertum Principes [Port-au-Prince], w/o date, Jaeger 208 (lectotype: P!, here designated; isolectotypes US!, W!, WU!).
Spondias myrobalanus sensu Sessé & Mociño, Flora mexic. 8(6): 119, pl. 105 (Torner coll. 0587, 0796). 1894.

#### Type

[icon]: Merian, Metamorph. Insect. Surinam 13, t. 13. 1705 (lectotype designated by Bornstein in Howard 1989).

#### Description.

*Hermaphroditic trees*, sometimes facultatively deciduous, reproductive height (3)6–25 m. Trunk 20–56 cm diam; *outer bark* brown or gray, rough (rarely smooth), usually deeply fissured, usually with corky, sometimes spinose projections, shed in rectangular plates; *inner bark* pinkish and orange-striate. *Trichomes* of two types: white and erect to 0.2 (0.3) mm long, and fine erect bristles to 0.05 (0.1) mm long. *Leaves* (1)3–10(–12)jugate, 14.5–42.5 cm long; petiole 3.5–13.5 cm long, petiole and rachis glabrous to sparsely pubescent; petiolules pubescent or glabrous, lateral petiolules (2–)3–10 mm long, terminal petiolule 5–40 mm long; basal leaflets 3.2–8.8 × 1.7–4.5 cm, (broadly) ovate, less often lanceolate or rotund, other laterals 5–25 × 2.4–6(8) cm, (oblong-)lanceolate to (oblong-)elliptic, less often ovate or oblong-oblanceolate, terminal leaflet 3.1–7.5 × 1.7–3.8(4.8) cm, lanceolate to narrowly ovate or elliptic; leaflet apex usually broadly and gradually acuminate, acumen 2–25(30) mm long, less often acute or rounded; lamina of lateral leaflets medially and usually basally asymmetrical, acroscopic side rounded to truncate or cuneate, basiscopic side cuneate to obtuse or attenuate, basal insertion usually asymmetrical and excurrent; leaflet margin flat and (sub)entire (on seedlings, first expanded leaflet blades crenate to serrate), sparsely ciliate with bristles; leaflets chartaceous to coriaceous, surface dull. *Inflorescences* (sub)terminal, developing when mature leaves are present, 15–60 cm long, 3–8 mm diam near base, broadly branched, secondary axes 2–24 cm long, axes with dense to sparse hairs to 0.3 mm long, sparser toward base or occasionally glabrous, also with sparse to dense bristles at branching points of inflorescences as well as on ultimate branches and on pedicel; bracts subtending inflorescences to 4.5 mm long, lanceolate and acuminate, bracts subtending secondary branches to 5 mm long, slightly broader than primary bracts, those on higher-order axes to 2 mm long, ovate, bracteoles 0.3–0.4 mm, deltate to ovate, bracts and bracteoles with usually dense bristles; pedicel 2–4.5 mm long overall, portion distal to articulation 1–2.6 mm long. *Calyx* 0.5–0.8 mm long, aestivation apert, lobes 0.2–0.6 mm long, (rounded-)deltate, with sparse hairs to 0.2 mm long and sparse bristles abaxially and on the margin; petals 2.5–3.2 × (1)1.3–1.4 mm, (ob)lanceolate, white, glabrous, reflexed at anthesis, apex slightly acuminate; stamens spreading, the antesepalous and antepetalous ones 2.5–2.7 and 2–2.3 mm long, respectively, the anthers 1–1.3 mm long, in dorsiventral view oblong, in lateral view oblong or less often elliptic; disk 0.3–0.7 mm tall, 0.1–0.2 mm thick, summit undulate and outer margin deeply sulcate, yellow; pistil 1.3–1.6 mm long, slightly ovoid overall, divided ca. half to 2/3 its length into subulate, apically divergent styles 0.7–1 mm long, stigmas extrorse, vertically elliptic. *Fruits* 2–4 × 1.8–2.7 cm, oblong or less often ellipsoid or slightly oblong-ovoid, maturing yellow or orange, apex and base rounded to truncate, surface smooth; endocarp oblong. *Seedlings* (fide Garwood 2009): first eophylls trifoliate, the petiole, rachis, and lower midvein with sparse stiff hairs, eophyll leaflets ovate, sparsely toothed, the margin glabrous.

*Leaflet venation*: Fimbrial vein present; secondary vein pairs 10–16, mostly straight, the spacing slightly decreasing near base only, the angle decreasing toward apex and increasing toward base, insertion decurrent; inter-secondaries present, average <1 per pair of secondaries and parallel to them, longer than halfway to margin but zig-zagging; intercostal tertiaries irregular-reticulate and admedially ramified, quaternaries irregular-reticulate and admedially ramified, areolation at tertiary and quaternary ranks; FEVs highly branched, dendritic, some slight thickening; fimbrial vein present; on abaxial side the midvein and secondaries prominent (secondaries rarely prominulous to flat), glabrous except midvein and secondary veins sometimes sparsely pubescent; on adaxial side the midvein prominulous or rarely flat, secondaries prominulous to impressed, glabrous.

#### Distribution.

*Spondias mombin* is widely cultivated in the moist tropics, but it is native in Mexico south to SE Brazil; it may be native to E Brazil but this is uncertain.

#### Ecology.

Native populations of *Spondias mombin* occur in tropical moist to semi-deciduous forests as well as gallery forests and forest islands in savannas, less often in floodplain forests (e.g., Little 8092, NY; see Peters and Hammond 1990); one collection is from white-sand dunes (Prance & Silva 24231, NY). In Central America, *Spondias mombin* has been considered a relatively early-successional species (Nason and Hamrick 1997). In drier or more open conditions, the bark tends to be thicker and to produce spinose projections; reportedly it insulates against fire damage to the cambium (Pinard and Huffman 1997).

Given this species’ broad distribution, its known phenology is broken down by region. Mexico: flowering Mar-May, fruiting May-Jul and Sep-Nov; Central America: flowering Mar-May (Sep); fruiting Mar-Oct; West Indies: flowering Mar-Jun (Dec); fruiting Apr-Aug (Dec); NW South America W of the Andes: flowering Nov-Jun (Sep), fruiting all year; N Venezuela and the Guianas: flowering Oct-Jun, fruiting Oct-Jun; W Amazonia: flowering Oct-May, fruiting Jan-Jun; NW Amazonia: flowering Oct-May, fruiting Jan-Jun; E and C Amazonian Brazil: flowering Jul-Apr, fruiting Nov; SW Amazonia: flowering Oct-Nov; fruiting Oct-Mar; C & E Brazil (S of the Amazon): flowering Aug-Feb, fruiting Sep-Apr.

In Rio de Janeiro state, Brazil, *Spondias mombin* is evergreen (Rodrigues and Samuels 1999), whereas in other parts of its range, such as C Panama and NW Costa Rica, it can be facultatively deciduous for up to two months (Croat 1974a and Janzen 1985, respectively). In Guanacaste Province, NW Costa Rica, the species flowers toward the end of the 6-month long dry season (late April to early May). In C Panama, flowering can range Feb-May, and the local period of flowering is ca. two months, with the trees of any given population hightly synchronized (Adler and Kielpinski 2000). At that same site, fruits required approx. five months to mature, and they ripened Jul-Oct with a peak in Aug-Sep. The fruiting season tends to be highly regular, but fruit production varies greatly among years (Milton et al. 2005).

In addition to the animals that are hunted below *Spondias mombin* trees (see below under Economic Botany), animals that disperse the fruits include deer, peccaries (collared and white-lipped), coatis, kinkajous, squirrels, spiny rats, agoutis, saki monkeys, several species of bats, and reptiles such as ctenosaurs (Hladik and Hladik 1969, Smythe 1970, Croat 1974b, Heithaus et al. 1975, Vàsques-Yanes et al. 1975, van Roosmalen 1981, Kiltie 1982, Orozco-Segovia and Vázquez-Yanes 1982, Janzen 1985, Fleming 1988, Barreto et al. 1997, Adler and Kielpinski 2000, Henry et al. 2000, Lobova et al. 2009).

#### Common names.

As noted, Morton (1981) listed 96 different common names for S. mombin. This species has been recorded as being called jobo in Belize, Cuba, the Dominican Republic, northern Colombia, Ecuador (Esmeraldas), Guatemala, Nicaragua, Mexico (Oaxaca, Veracruz), Panama, and Puerto Rico, and called hog plum in Belize, Jamaica, Tortola, and Trinidad and Tobago. The species is generally called taperebá (taperibá) in Brazilian Amazonia but more commonly called cajá or cajazeiro in the rest of Brazil (Ducke 1946, Smith et al. 2007). Other common names include the following. Bolivia, Beni: cedrillo (Oscar et al. 1000, NY), aquiachá (Sirionó, Vargas et al. 423, NY), Santa Cruz: azucaró de monte; nusucarr (Toledo et al. 556, NY); Brazil, Acre: cajá (Daly et al. 10175, NY); Amazonas: taperebá (Krukoff 8329, NY); Bahia: cajá mirim (F. Souza Santos 820, NY), cajarana (Hage 2186, NY); Pará: kaijuwa’ywa (Assurini, Balée 2569, NY), tawa-wa-’y (“yellow fruit tree,” Guajá Indians, Balée 3380, NY), taperiwa’y (Ka’apor, Balée 2212, NY), akãija’i (Araweté, Balee 2033, NY), taperebá (S. A. M. Souza et al. 1226, NY); Roraima: cajá (Portuguese), canaxaron (Uaicá-Mucajaí, Prance et al. 10979, NY); Dominica: mobin (creole-patois, Stijfhoorn et al. 867, GH, NY); Dominican Republic: jobo de puerco (Ososki & Saborío 468, NY); El Salvador: jocote jobo (Villacorta & Giammattei 2548, NY); Guadeloupe and Martinique: monbin, prûne monbin (Duss 3272, 322, NY), faux mirobolan (Tussac Fl. Antill. 4: 97, tab. 33. 1827); Guyana: plumtree (English), kubu (Arawak), Usiarao (Wr)(Reinders 81, NY); Jamaica: Jew plum (Yuncker 17086, NY); Mexico, Oaxaca: beea-chi (Zacateca, A. Miller et al. 318, NY), jumuy (Zoque, Hernández G. 2695, TEX), a^2^ hma^3^ o^3^ nei^23^ (Chinantec, Sabino, s.n., NY); Puebla: kwawxokot (Nahuatl, Mendoza & Amith 1430, NY); San Luis Potosí: k’inim (Huastec, Alcorn 1519, TEX); Netherlands Antilles (Curaçao): hoba (Arnoldo-Broeders 3902, NY); Nicaragua: jocote jobo (Guzmán et al. 578, NY), walak (Ulwa; Coe 2275, MO); Panama: jobo de montaña, (Miller et al. 261, NY); Surinam: mopé (Carib), hobo (Arawak)(Stahel 168, A); Venezuela, Amazonas: mopiyo’ (Panare, Boom & Grillo, NY), tanomami (Yanomami, Fernández 6814, NY).

#### Economic botany.

The range and habitats of introduced *Spondias mombin* overlap significantly. The species has a long pre-Columbian history of use (e.g., Ducke 1946); carbonized endocarps are abundant in middens of the extinct Marajoara culture of Marajó Island at the mouth of the Amazon (Roosevelt 1991, as *Spondias lutea*), and it is described and illustrated (as *caia*) in Frei Cristóvão de Lisbôa’s *História dos Animais e Árvores do Maranhão* (1968, fascimile of ca. 1625 manuscript). It continues to be an important plant resource in Amazonia (Smith et al. 2007). In the West Indies, it was probably introduced, as suggested by its occurrence primarily in disturbed areas. It is well-established as an invasive species in tropical West African forests and savannas (Ghazanfar 1989). It is more commonly cultivated in tropical Africa than S and SE Asia (Kostermans 1991).

Most collections that cite uses note the edible fruit. The species can be dominant in some periodically flooded riverine habitats (Peters and Hammond 1990), and individual trees can be highly productive, producing up to 10,000 fruits per tree (Adler and Kielpinski 2000). The second most reported observation is that the fruits of *Spondias mombin* are eaten by game animals and in these cases *Spondias mombin* serves as a “waiting tree” where locals go to hunt in the fruiting season. The animals include *Ateles* (Croat 12291, NY), *Alouatta* and *Cebus* monkeys (Balée 3380 and 2569, NY, respectively) as well as yellow-footed tortoises and pacas (Balée 2033, NY), tapirs (Ayres and Ayres 1979), and toucans (Miller et al. 217, NY).

Ayoka et al. (2008) provided a useful review of the economic and traditional uses of the species. The primary use of the species is for its fruits, reportedly high in vitamins C and B1 (Keshinro 1985, Bora et al. 1991). The pulp is stewed, or made into preserves, or used to prepare juices and alcoholic beverages (fermented or for flavoring)(Cavalcante 1976, Lorenzi et al. 2006); one fermented product is referred to in Brazil as *vinho de taperebá*. (Severo et al. 2007) The juice is available in restaurants and foodstores in Brazil, and the frozen pulp is commercialized throughout the country. The tree is commonly planted as a living fence or in home gardens, or planted for shade and food for livestock (Morton 1981). In the Dominican Republic the fruits are fed to pigs (*Zanoni et al. 3028*, NY).

Other important uses of *Spondias mombin* are in traditional medicine (see review in Duke et al. 2009), both in its native range and where it has been introduced. The ethnobotanical literature and herbarium specimen labels provide many accounts of the uses of its roots, leaves, flowers, fruits (rarely) and especially bark for medicinal purposes, to treat myriad medical problems such as wounds, fever (Balée 2569, NY), dysentery, vaginal bleeding, genital ulcers, respiratory conditions, intestinal and digestive ailments, (Morton 1981; Grenand et al. 2004), malaria (Milliken 1997), leishmaniasis (Fleury 1991), colds (Yuncker 17086, NY), and as a contraceptive and abortifacient (Offiah and Anyanwu 1989, Schultes and Raffauf 1990). Most often the preparations are infusions or decoctions that are either ingested or applied to the affected area. In Amazonia, the inner bark is ground into a powder and used as a disinfectant for wounds, and the boiled powder is used as an oral disinfectant (Nelson 749, 785, NY). In Nicaragua, a decoction of bark and leaves is used to treat malaria, diarrhea, infections, skin rashes, and sores (Coe 2275, MO).

Recently, compounds purified from *Spondias mombin* have been tested for a range of biological activity in lab animals, including antifungal, antimicrobial (Corthout et al. 1994, Rodrigues and Hasse 2000), anthelminthic (Ademola et al. 2005), anti-viral (Corthout et al. 1991, Ayoka 2008), and psychoactive properties (Akubue et al. 1983, Ayoka et al. 2006).

The wood of *Spondias mombin* is used as a fuelstuff (Balée 2212), but it is of poor quality because it is susceptible to rot and attack by insects (Record 1939, Ter Welle et al. 1997); even so, it is used occasionally for construction, carpentry, and fenceposts (Ayoka et al. 2008). The thick bark is carved to make handcrafts.

#### Additional specimens examined.

**BELIZE**. Little Coquericot, Belize River, 27 May 1933, Lundell 4356 (K). **BERMUDA**: Montrose, Sep 1913, Brown et al. 1656 (NY). **BOLIVIA. Beni**: Prov. Yacuma, Bosque de Chimanes, ca. 65 km SE of San Borja and 65 km SW of San Ignacio, Fatima logging concession, along base of Serranía Eva Eva, S of Río Chinzi near logging camp El Combate, ca. 15°30'S, 66°15'W, 27 Oct 1989, R. Foster & W. Terceros 13386 (F, NY); **La Paz**: Prov. Iturralde, 3 km NE of Buena Vista, 14°22'S, 67°33'W, elev. 180 m, 25 Apr 1995, DeWalt 316 (MO); **Santa Cruz**: Prov. Velasco, Reserva Ecológica El Refugio, 1 km W of camp on trail to saltpeter mine of Cerro La Pista elev. 250 m, 14°45'53"S, 61°02'21"W, 25 Jan 1995, Guillén & Roca 3034 (NY). **BRAZIL Acre**: Mun. Xapuri, Rio Acre, @ 3 hrs. by boat downstream from Xapuri and 1 hr. walking inland from left bank, 10°45'S, 68°20'W, 6 Nov 1991, Daly et al. 7174 (HUFAC, NY); **Alagoas**: Mun. Quebrangulo, Reserva Biológica de Pedra Talhada, 9°15'S, 36°25'W, 13 Jan 1994, Cervi et al. 7358 (NY); **Amapá**: Rio Oiapoque, E of Cachoeira Manauá, 2°18'N, 52°38'W, 17 Sep 1960, Irwin et al. 48317 (MG, NY); **Amazônas**: Mun. Manaus Reserva Florestal Ducke, km 26 Manaus-Itacoatiara road, 8 Jul 1995, M. Hopkins et al. 1454 (NY); **Bahia**: Ilhéus, campus of Centro de Pesquisas do Cacau, 14 May 1965, Belém & Magalhães 973 (NY); **Goiás**: Mun. Vila Boa, near Vila Chamada, ca. 15°10'S, 47°00'W, ca. 650 m, 19 Oct 1995, B. Pereira & D. Alvarenga 2895 (NY); **Rio de Janeiro**: Rio de Janeiro, Morro do Telégrafo, 18 Oct 1930, Brade s.n. (R 73761 (R). **COLOMBIA**. **Amazonas**: Aduche, Asentamiento Muinane, south bank of río Caquetá, 0°41'45"S, 72°05'45"W, 11 May 1999, Arévalo A. & Reyes R. 58 (NY); **Antioquia**: Mpio. San Luis, Corr. El Prodigio, Finca Dormene y Serranías, 6°06'N, 74°48'W, elev. 350–400 m, 25 Jun 1990, Cárdenas et al. 2866 (MO); **Bolívar**: Mun. Acandí, Corr. Triganá, Reserva Zazardí, 8°20'N, 77°10'W, elev. 100 m, 23 Mar 2006, Cardona-N. et al. 1644 (NY); **Chocó**: Mpio. Acandí, Corr. Triganá, Reserva Zazardí, 8°20'N, 77°10'W, elev. 100 m, 23 Mar 2006, Cardona et al. 1644 (NY); **Magdalena**: Santa Marta, elev. 800 m, 1898–1901, H. H. Smith 912 (COL, GH, MPU); **Meta**: Parque Nacional Natural Tinigua, Serranía Chamusa, Centro de Investigaciones Primatológicas La Macarena, Aug 1990, Stevenson 178 (MO). **COSTA RICA**: **Guanacaste**: Parque Nacional Palo Verde Area de Conservación del Tempisque, main trail do Sendero Guayacancito, 10°21'N, 85°22'W, 27 Mar 1992, Chavarría 590 (NY). **CUBA.** Santa Clara, Guajimica, 23 Mar 1910, Britton et al. 5817 (NY). **CURAÇAO**: Christoffelpark, 8 Feb 1999, van Proosdij et al. 581 (NY). **DOMINICAN REPUBLIC**: **Prov. Barahona**, Sierra de Baoruco, at the cross of El Platán, 9.4 km NNE of Paraiso on road parallel to Río Nizaito, 18°03.5'N, 71°13'W, 23 May 1984, Zanoni et al. 30298 (NY). **ECUADOR**. **Esmeraldas**: Mataje, left bank of río Mataje, elev. 140 m, 9 Sep 1991, Jaramillo et al. 13845 (MO); **Napo**: Estación Científica Yasuní, Río Tiputini, NW of confluence with Río Tivacuno, 6 km E of Carretera Maxus, km 44 of branch to pozo Tivacuno, 0°59'S, 77°45'W, elev. 200–300 m, 26 Jun 1999, Romoleroux & Grefa 3239 (NY). **Guayas**: Guayaquil, highway W of town, 8 Mar 1955, Asplund 15634 (NY, R). **EL SALVADOR**. **Ahuachapán**: San Francisco Menéndez, beach of río San Francisco, 13°49'N, 89°56'W, 4 Mar 1994, S. Martínez s.n. (LAGU ISF0063) (MO). **HONDURAS**: Valle San Francisco, near El Zamorano, 13°58'11"N, 86°59'38"W, 789 m, 13 July 2001, Miller et al. 149 (NY). **FRENCH GUIANA**. Mont La Fumée, 3°37'N, 53°12'W, elev. 200–400 m, 4 Dec 1982, Mori & Boom 15324 (NY). **GRENADA**: St. George, Annandale Falls, 12°05'N, 61°43'W, 12 Jun 2001, Hawthorne et al. 480B (NY). **GUATEMALA**. **Izabal**: vicinity Lago Izabal, 15°15–25'S, 89°0–25'W, 2 May 1966, G. Jones & L. Facey 3240 (NY). **GUYANA**: **Barima-Waini Region**, Waini Peninsula, Shell Beach Sea Turtle Monitoring Camp, 8°23'57"N, 59°45'14"W, elev. 1 m, 5 May 2000, Hollowell 309 (NY). **JAMAICA**: **St. Andrew**: Campus Univ. College of West Indies, Mona, 15 Oct. 1957, Yuncker 17086 (NY). **MEXICO**. **Chiapas**: Escuintla, Jul 1938, Matuda 2617 (A, NY, TEX). **Guerrero**: Arcelia, Coyuca, 15 Sep 1934, Hinton 6599 (BM, GH, NY); **San Luis Potosí**: Mpio. Aquismon, Tancuime, 23 Aug 1978, Alcorn 1519 (TEX); **Sinaloa**: Mazatlán, 10 m, 26 Feb 1926, Gonzáles O. 5670 (K); **Tabasco**: roadside on levee, Aldama, near Comalcalco, 8 May 1963, West 25/1 (GH); **Yucatán**: Mun. Sucilá, 3 km W of Sucilá, toward Buctzotz, 21°14'30"N, 88°20'45"W, 22 Sep 1996, Durán et al. 2616 (GH, TEX). **NICARAGUA**: **Carazo**: Quebrada La Chota, trib. of río Escalante, ca. 7 km NE of Chococenter station, ca. 11°35'N, 86°09'W, elev. 100 m, 19 Mar 1983, A. Grijalva 2415 (MO). **PANAMA**. **Bocas del Toro**: Punta Peña, 8°54'52"N, 82°11'10"W, elev. 90 m, 30 Aug 2001, Miller et al. 259 (MO). **PARAGUAY**. **Central**: Trinidad, Asunción, Jardín Botánico y Zoológico [likely cultivated], 25°20'S, 57°28'W, Dec 1991, B. Pérez 1426 (NY). **PERU**. **Huánuco**: Prov. Puerto Inca, Dtto. Yuyapichis, Unidad Modelo de Manejo y Producción Forestal Dantas, 9°40'S, 75°02'W, 115 Oct 1990, Tello 258; **Lambayeque**: Prov. Chiclayo, Reque, 25 m, 7 Mar 1994, Quiroz 3451 (NY); **Loreto**: lower Río Huallaga, elev. 155–210 m, Oct-Nov 1929), Williams 4933 (A); **Madre de Dios**: Tambopata, Comunidad Nativa de Infierno, Hermosa Chica, 12°50'S, 69°17'W, 260 m, 10 Feb 1989, Alexiades & Pesha 262 (NY). **PUERTO RICO**: Caribbean National Forest, near Catalina Field Office, 18°17'N, 65°47'W, 31 July 1986, Boom & Rivera 6793 (NY); **SURINAM**: Tumuc Humac mountains, Litani River, 2°31'N, 54°45'W, elev. 190 m, 14 Aug 1993, Acevedo-Rodríguez et al. 6033 (TEX). **TRINIDAD**: coastal hillside, Pointe Gourde, 31 Mar 1921, Britton & Broadway 2647 (NY). **UNITED STATES**. **Florida**: Monroe County, Homestead, cultivated at Subtropical Experiment Station 19 Oct 1971, Gillis 11119, MO). **VENEZUELA**. **Amazonas**: Depto. Río Negro, elev. 540 m, 2°16'N, 63°31'W, Oct 1991, Chaviel 52 (NY); **Apure**: Distrito Pedro Camejo, bank of the río Orinoco, 35 airline km NE of Puerto Paéz, just NE of Isla El Gallo, 6°05'N, 67°13'W, elev. 40 m, 23 Feb 1978, Davidse & González 14459 (NY); **Aragua**: Tovar, elev. 3000 ft., 5 Jun 1855, Fendler 1310 (GH). **Bolívar**: Mun. Padre Chien, 24 km from El Palmar toward Río Grande, elev. 275 m, 8°01'30"N, 61°51'40"W, 12 Apr 1997, Diaz et al. 3172 (NY); **Zulia**: Dtto. Páez, near Misión de Guana, between highway and km 4 of road E of CarrasqueroGuanaGuarero highway, elev. 100–150 m, 5 Jun 1977, Bunting 5130 (NY).

#### Conservation status.

We consider this species to be of Least Concern because of its broad range and often large populations, moreover it is widely cultivated.

#### Discussion.

This species is compared to *Spondias globosa* in the discussion under the latter species. The occurrence of a number of distinct intermediates between *Spondias mombin* and several other species suggests the possibility that this species is prone to hybridization (see discussion above in sections treating the genus as a whole).

### Spondias purpurea

Taxon classificationPlantaeSapindalesAnacardiaceae

L., Sp. pl., ed. 2, 1: 613. 1762.

**Figure 22.**
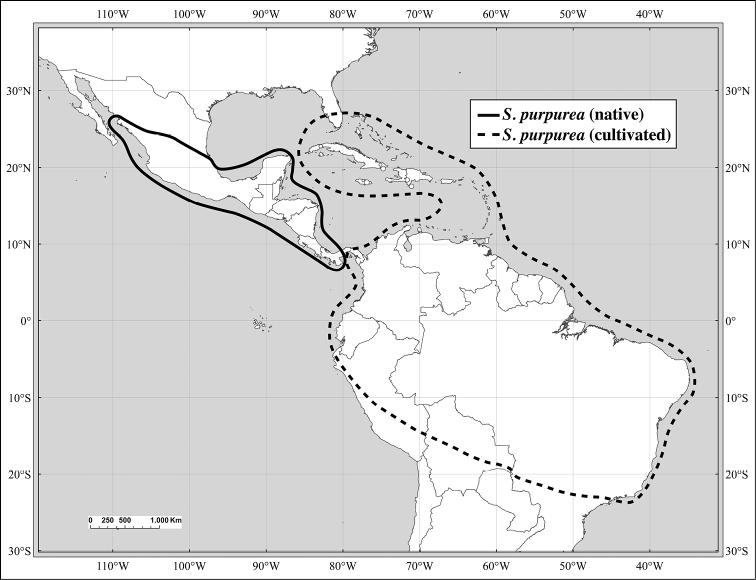
Distribution of *Spondias purpurea*.

Figs 2 , 3 , 4 , 14 , 16 , 22

Spondias myrobalanus L., Fl. jamaic. 16. 1759, nom. illegit. (based on same type as *Spondias purpurea*, vide Jarvis 2007: 871; not homotypic with *Spondias myrobalanus* L., Syst. nat., ed. 10, 2: 1036. 1759). Type. Based on *Spondias purpurea* L.
Spondias myrobalanus sensu Jacq., Select. stirp. amer. hist., 139, t. 88. 1763, non L., Fl. jamaic., 1759.
Spondias cirouella sensu Tussac, Fl. Antill. 3: 37, t. 8. 1824, excl. synonyms of *Spondias mombin* L. *Spondias jocote-amarillo* Kosterm., Kedondong, Ambarella, Amra – The Spondioideae (Anacardiaceae) in Asia and the Pacific area: 27. 1991, nom. nov. based on *Spondias cirouella* Tussac).
Spondias purpurea Type [icon]: Tussac, Fl. Antill. 3: 37, t. 8. 1824 (lectotype, designated by Kostermans, l.c.: 27).
Warmingia pauciflora Engl. in Mart., Fl. bras. 12(2): 281, t. 57. 1874.
Spondias purpurea Type. Peru. Tarapoto (cult.), Jul 1855, Spruce 4093 (lectotype: K!, here designated; isolectotypes: C, GH!, P!).
Spondias mexicana S. Watson, Proc. Amer. Acad. Arts 22: 403. 1887.
Spondias purpurea Type. Mexico. Jalisco: Tequila, Aug-Sep 1886, E. Palmer 408 (lectotype: US-2 sheets!, here designated; isolectotypes: GH!, K!, NY!, PH!).
Spondias purpurea L. var. munita I.M. Johnst., Sargentia 8: 182–183. 1949.
Spondias purpurea Type. Panama. San José Island, Perlas Archipelago (55 mi. SSE of Balboa), 29 Mar 1945, *I.M. Johnston 573* (lectotype: US!, here designated; isolectotype: GH!).
Spondias negrosensis Kosterm., Kedondong, Ambarella, Amra – The Spondiadeae (Anacardiaceae) in Asia and the Pacific area: 27. 1991.
Spondias purpurea Type. Philippines, Luzon, Rov. Rizal, Panay, Merrill, Sp. blancoan. 639 (holotype: BO n.v.; isotypes: NY!, US!, W!).

#### Type

[icon]. “*Myrobalanus minor folio fraxini alato, fructu purpureo*” etc. (lectotype, designated by Bornstein in Howard 1989: Sloane, Voy. Jamaica 2: 126, t. 219, Figs 3–5. 1725. (Typotype): herb. Sloane 7: 66 (BM-SL!)).

#### Description.

*Dioecious trees*, sometimes shrubby and broadly branching, often deciduous for long periods, reproductive height 3–15 m.Trunk 10–50 cm diam, *outer bark* pinkishgray to dark gray, smooth or ornamented with corky spinose projections to 6.2 cm long (*Johnston 964*, GH), sometimes with spinose short shoots; *inner bark* whitish with brown striations. *Trichomes* of three types: uncinate to crispate (rarely erect) 0.1–0.2 mm long, erect bristles to 0.05 mm long (only sometimes on calyx), and capitate glandular hairs (when present, on bract margin, distally on pedicel, and on calyx). Often leafless for extended periods. *Leaves* 2–13jugate, 6–28 cm long; petiole 2–5.2 cm long, rachis sometimes subalate, petiole and rachis glabrous or with dense uncinate hairs; lateral petiolules 1–4 mm long, the terminal one to 15 mm long, hairs as on rachis; basal leaflets 1.4–5 × 0.9–2.6 cm, obovate, other laterals 3–6.8 × 1–2.7 cm, elliptic, oblanceolate, or obovate, terminal leaflet 2.6–5.6 × 1.1–2.9 cm, obovate; leaflet apex obtuse to acute, occasionally retuse or slightly acuminate, the acumen to 5 mm long, apex tip mucronate and glandular, often breaking off; lateral lamina usually medially and basally asymmetric, the acroscopic side of base obtuse, the basiscopic side cuneate or attenuate, basal insertion symmetric and decurrent, leaflets chartaceous and dull; margin flat, entire to serrulate toward apex, the teeth irregularly spaced, concavo-convex, the tooth apex setose (setae often deciduous). *Inflorescences* axillary and trees often ramiflorous, developing before or during leaf flush, staminate inflorescences 1–16 cm long, 2–3 mm diam near base with secondary axes to 1.4 cm long, the pistillate inflorescences 1–4.5 cm long with secondary axes to 8 mm long; axes glabrous or with sparse to dense uncinate hairs; bracts subtending inflorescences to 1.5 mm long, those subtending secondary axes to 1–2 mm long, bracteoles 0.6–0.8 mm long, lanceolate to ovate, apex acute; pedicel (0.5) 1.7–3.6 mm long, portion distal to articulation 0.3–1 mm long, the distal portion sometimes with capitate glandular hairs. *Calyx* 1–1.6 mm long overall, aestivation quincuncial, lobes 0.7–1 mm long, rotund to broadly ovate, usually red to purple (Fonnegra & Corral 1633, NY), abaxially with sparse to dense erect bristles to 0.05 mm, the margin ciliate; corolla aestivation quincuncial, petals 2.5–3(3.5) × 0.85–1.2 mm, oblong-lanceolate to narrowly ovate, apex acute, usually red to purple, sometimes yellow, cream, or white, glabrous, the margin ciliate with straight to curved whitish hairs to 0.1 mm, suberect at anthesis; in *staminate flowers* the stamens suberect, the antesepalous and antepetalous ones 2–2.35 and 1.45–1.7 mm long, respectively, the anthers 0.8–1 mm and 0.65–0.85 mm long, respectively, in dorsiventral view broadly oblong to ovate, in lateral view oblong, the disk 0.25–0.5 mm tall, 0.4–0.7 mm thick, summit rugose and outer margin crenellate, pink; pistillode consisting of (3)4–5 erect, awn-shaped, parenchymatous styles 0.6–1 mm long; in *pistillate flowers* the antesepalous and antepetalous staminodes 2.3–2.5 and 1.5–2 mm, respectively, the anthers 0.4–0.55 and 0.5–0.6 mm, respectively, in dorsiventral view broadly oblong (antesepalous ones) or ovate (antepetalous ones), in lateral view oblong; disk 0.4–0.6 mm tall, 0.25–0.5 mm thick, summit very slightly undulate and outer margin nearly entire; pistil 1–1.5 mm long, depressed-ovoid to subcylindrical overall, tinged red toward apex, divided less than half its length into thickly subulate, divergent styles ca. 0.3–0.8 mm long, shorter than the ovary and separated by the flat roof of the ovary, each terminating in a thin, introrse, obovate stigma. *Fruits* 2.5–5 × 0.5–3 cm (dry), oblong to obovoid, maturing yellow to (reddish) orange or red, the surface smooth, often glossy; pyrene oblong, bony. *Seedlings* (Magallanes 3887, NY) with linear cotyledons (Duke 1965), first eophylls alternate and trifoliate, serrate, subsequent ones pinnate.

*Leaflet venation*: Fimbrial vein absent; secondary veins 5–10 pairs, usually arcuate, spacing decreasing toward apex and increasing toward base, angle acute and slightly irregular but increasing toward apex and decreasing toward base, insertion excurrent or decurrent; intersecondaries average <1 per intercostal area, perpendicular to midvein and slightly acroflexed; epimedial tertiaries 1 per intercostal area, perpendicular to midvein; intercostal tertiary fabric irregular-reticulate and strongly admedially ramified; quaternaries irregular-reticulate and freely ramified, areolation at tertiary and quaternary ranks, FEVs highly branched, dendritic, with some terminal thickening; marginal ultimate venation mostly looped; on abaxial side the midvein and secondaries flat to prominulous, glabrous except midvein often sparsely pubescent toward the base, the surface sparsely to densely micro-pustulate, on adaxial side the midvein prominulous and secondaries flat to prominulous, glabrous except the midvein sparsely to densely pubescent.

#### Distribution.

*Spondias purpurea* naturally grows in tropical deciduous forests from NW Mexico to Panama and possibly N Colombia; its cultivated distribution extends well beyond its native distribution, and its true range is complicated by its long association with humans. In some areas where the species has been assumed to be native, some evidence suggests that it has been introduced; for example, Janzen (1985) reported that *Spondias purpurea* and *Spondias mombin* co-occurred in Guanacaste Province in Costa Rica, but he observed that the former was usually found near roads and trails. Miller (Miller and Schaal 2005) and others have noted that *Spondias purpurea* produces fruit parthenocarpically where it is not native, and it is known that the species is propagated asexually in many places, but the observations of Janzen and others suggests that it can reproduce sexually outside its native range.

#### Ecology.

*Spondias purpurea* grows in highly seasonal tropical (semi-)deciduous forests (Miller and Knouft 2006), and it appears to thrive best in dry conditions; for example, at Chamela Biological Reserve in Jalisco, Mexico, it was much less abundant in semi-deciduous forest than in adjacent deciduous forest (Mandujano et al. 1994), where it is sympatric with *Bursera* spp., *Cyrtocarpa procera* and *Comocladia* sp. (Lott 2002). Still, it thrives in a broad range of habitats and soil types, and its cultivated range is primarily in more humid environments. It has been found (probably cultivated) at elevations up to 1200 m in both Mexico and Ecuador.

Given this species’ broad distribution, its known phenology is broken down by region. Mexico: flowering Dec-Aug, fruiting May-Sep; Central America: flowering Dec-Sep, fruiting Mar-Oct; NW South America west of the Andes: flowering Feb-Oct; remainder of N South America: flowering (Jan) Sep–Nov.

Several studies in Mexico have shown that this species is markedly deciduous and flowers when leafless, while the fruits mature near the interface of the dry/leafless and wetter/leafy seasons. In Sinaloa, the trees were leafless from Jan-May, flowering in Feb-Mar and fruiting in Jun, and in Puebla the trees were leafless Jan-Apr, flowering Dec-Jan and fruiting Apr–May (Cuevas 1994), while in Chamela (Jalisco) the trees were leafless Nov-Jun, flowering in Feb and the fruits maturing in May (Bullock and Solís-Magallanes 1990).

*Spondias purpurea* dispersal was studied in detail by Mandujano et al. (1994) in a deciduous forest in Chamela, Mexico. They found that the major dispersers were white-tailed deer, collared peccaries (see also Martínez-Romero and Mandujano 1995), coatis, gray foxes, chachalacas, orioles, and ctenosaurs. At Chamela there was no evidence of bat dispersal, although Lobova et al. (2009) cite references containing observations of this species being dispersed by several species of bats. White-faced capuchin monkeys were observed dispersing fruits of *Spondias purpurea* at Santa Rosa National Park in Costa Rica (Freese 1977).

#### Common names.

This species has been recorded as being called ciruela or ciruelo in Colombia, the Dominican Republic, Mexico (Jalisco), Puerto Rico, Brazil (as ciriguela), Ecuador, Panama, Honduras, and Bolivia, and called jocote in Costa Rica, Nicaragua, Belize, Panama, Honduras, and El Salvador. Other common names include the following: Bahamas: Hog plum (Howard & Howard 10049, NY); Belize: ab-úl (Maya, Balick et al. 2467, NY), huhun (Maya, Lentz et al. 2436, NY), mombin, golden plum, hog plum (Riesema & Beveridge 52208, NY); Colombia, Antioquia: hobo colorado, jobo colorado (Fonnegra G. & Corral 1633, NY); Costa Rica: jocote invierno (Miller & Paschke 206, NY); Dominican Republic: jobo (Peláez F. 1230, NY); El Salvador: jocote de iguana (Rosales 2198, NY); jocote de pava (Rosales 297, NY; Sandoval 1210, MO); French West Indies: monbin cirouellier (Tussac 3: 37, t. 8. 1824); French Guiana: mope, puune (Boní; Fleury 729, NY); sewal (Haitian; Prévost 1988, NY); Grenada: Chili plum (Broadway s.n., NY); Guatemala: té-pah pom, jocote de coche (Castillo & Castillo 1792, NY); Guyana: Surinam plum (Omawale & Persaud 118, NY); Honduras: jocote rojo, tronadora (Miller et al. 149, NY), jobo de chancho (Molina & Molina 35164, NY); Jamaica: plum (Yuncker 18612, NY); Martinique: prune d’Espagne (Duss 326, NY); Mexico, Chiapas: xo-ko (Nahuatl, Miller et al. 324, NY); jobo (Miller et al. 327, NY); pitch-kuhl (Popoluca, Miller et al. 323, NY); Guerrero: ciruelo de jardín (Germán et al. 239, NY); México: ciruelo del zorro (Hinton 3217, NY); Oaxaca: cuachalalate (Flores M. 1218, NY); Sinaloa: ciruelo de coyote (Gentry 7108, NY); Puebla: guajite (Nahuatl, Mendoza & Amith 1447, NY), ciruela simarrón (Spanish, Mendoza & Amith 1447, NY); Veracruz: ciruelo natural (Miller et al. 314, NY); Nicaragua: walak (Ulwa, Coe 2959, MO) Puerto Rico: jobillo (Little, Jr. 16442, NY); Panama: ciruela san juanero (Miller et al. 294, NY), ciruela morada (Miller et al. 237, NY), jobito (Miller et al. 258, NY), ciruela traqueadora (Miller et al. 241, NY); ciruela amarilla (Miller et al. 252, NY); ciruela de casa (Miller et al. 255, NY); Peru. San Martin: ushún (Schunke Vigo 14495, NY).

#### Economic botany.

Some cultivated populations preserve genetic diversity of the species which may have been lost from wild populations (Miller and Schaal 2005); this is due to the highly fragmented and reduced extent of tropical dry forests in Mexico and Central America (Mooney et al. 1995). In addition to being introduced and possibly naturalized in tropical America, it has also been introduced in the Paleotropics, especially in the Philippines (not coincidentally a former Spanish colony).

Most cultivated populations of *Spondias purpurea* are apparently parthenocarpic (Juliano 1932, Miller and Schaal 2005); the major means of propagation is by stem cuttings (Cuevas 1994, Macía-Barco and Barfod 2000). The species is sometimes grown in large orchards, but it is most commonly planted as a living fence or as individual fruit trees in home gardens.

The primary use is for its fruits, which are eaten raw or made into juices, alcoholic beverages (for flavoring or fermented), or less often preserves (Morton 1987, Cuevas 1994, Macía-Barco 1997). The fruits are reportedly high in vitamin C (Kolzio and Macía-Barco 1998). The fruits of cultivated varieties range in color from red, orange, green, or purple; the pulp (mesocarp) is usually thicker, sweeter and less acid than that of wild populations. The leaves and young shoots have been boiled or used in salads (Lim 2012). Phylogeographic evidence suggests multiple domestications of the species (Miller and Schaal 2005, 2006; Miller 2008).

A limited number of medicinal uses for the species have been recorded. In French Guiana it has been used to purify the blood (“clarifie le sang,” Prévost 1988, NY; plant part and preparation not specified). In Panama, a leaf infusion has been used for skin problems (*Miller et al. 260*, NY). In Sinaloa, Mexico, the leaves are eaten raw (*H. S. Gentry 7108*, NY). In Nicaragua, a decoction of the bark and leaves is used as an abortifacient and to treat fever, malaria, diarrhea (Coe 2959, MO).

#### Selected specimens examined.

**BAHAMAS**. **Andros**: Mangrove Cay, along ridge road in Grant’s Town, 23 July 1978, Correll 50042 (NY). **BELIZE**. **Cayo District**: Arenal Village, on Guatemalan border near Benque Viejo del Carmen, 17 Aug 1981, Ratter R4669 (NY). **BOLIVIA**. **Santa Cruz**: Prov. Andrés Ibánez, 8–10 km S of center of Santa Cruz city, 17°52'S, 63°10'W, 1 Jan 1992, B. Mostacedo 237 (MO); Prov. Velasco, Parque Nacional Noel Kempff M., Campamento La Torre, 13°38'24"S, 60°47'45"W, 23 Nov 1993, Quevedo et al. 2548 (NY). **BRAZIL**. **Acre**: Mun. Rio Branco, Fazenda Experimental Catuaba, off km 22 of BR-364 (Rio Branco-Porto Velho), 500 m from highway by house along un paved road leading to station, 10°06'S, 67°36'W, 23 Sep, Daly 13105 (HUFAC, NY); **Amazonas**: Manaus, Chapéu de Palha, Vila Municipal (cultivated), 22 Dec 1973, Prance & Steward 20105 (NY); **Bahia**: Mun. Juazeiro, 7 km S of Juazeiro along BR407, grounds of Pousada Juazeiro (cultivated), 9°25'S, 40°35'W, 23 Jan 1993, Thomas et al. 9567 (NY); **Mato Grosso do Sul**: Mun. Jardim, Boqueirão, (cultivated), 14 Mar 2004, Hatschbach et al. 77113 (US). **COLOMBIA**. Antioquia: Mpio. de Venecia, 4.2 km E of Bolombolo on road to Venecia, Hacienda La Plata (cultivated), 6°01'N, 75°48'W, elev. 920 m, 12 Mar 1987, Zarucchi & Echeverry 4665 (MO, NY); **Chocó**: Riosucio, Sautata, Parque Nacional Las Katios, 7°50'N, 77°06'W, 90 m, 11 Feb 1992, Palacio 25 (MEDEL); **Huila**: outskirts of Garzón town, near 2°11'57"N, 75°38'59"W, elev. ca. 780 m, 23 Oct 2010, (cult.) Daly et al. 13968 (COAH, NY). **Santa Marta**: Masinga, elev. 250 ft., 27 Mar 1898, H. H. Smith 1746 (GH, NY). **COSTA RICA**. **Guanacaste**: Cantón Bagaces, Valle del Tempisque, Sendero La Venada y Sendero Guayacancito, 10°21'N, 85°21'W, 24 May 1994, Chavarría 958 (NY); **Puntarenas**: Cabo Blanco Nature Reserve, 0–200 m, 9°35'N, 85°06'W, 1–7 Dec 1969, Burger & Liesner 6667 (NY). **CUBA**. **Camaguey**: Cayo Ballenato Grande (cultivated), 22 Mar 1909, Shafer 1035a (NY); **Pinar del Río**: Guayabal, 24 Feb. 1911, Britton et al. 9592 (NY). **DOMINICAN REPUBLIC**. **La Vega**: 2–3 km from Higuero (de Bayacanes) toward Jagua Gorda, 19°15'N, 70°36'W, elev. 280 m, 29 Sep 1981, Zanoni et al. 16840 (NY). **ECUADOR**. **Esmeraldas**: Borbón, edge of town (“planted?”), 25 Apr 1943, Little 6366 (NY); **Guayas**: Isla Puná, path from Puná Nueva to Las Pozas, 2°45'S, 79°54'W, 28 May 1987, J. Madsen 63428 (NY); **Loja**: Bosque Petrificado de Puyango on Loja side of river, 3°54'S, 80°54'W, elev. 380 m, 24 Aug 1996, Lewis 2520 (MO). **EL SALVADOR. Ahuachapán**: Río Paz, along Canaleto Road, 13°54'02"N, 90°01'96"W, elev. 101 m, 6 Mar 2002, Monro et al. 3624 (MO). **FRENCH GUIANA**: Bourg de Maripasoula, basin of Maroni River (cultivated), 3°37'N, 54°05'W, 9 Dec 1988, Fleury 729 (NY). **GRENADA**: St. Patrick, River Sallee, 12°12'N, 61°37'W, 15 June 2001, Hawthorne et al. 519 (FHO, NY). **GUATEMALA**. **Alta Verapaz**: Kobán, elev. 1350 m, 1907, von Türckheim II=1778 (GH); **Izabal**: Puerto Barrios, ca. 20 km from town on road to Machacas (cultivated), 15°46'N, 88°32'W, 9 Mar 1988, Marshall et al. 356 (NY). **GUYANA**: **Demerara**: east coast, Nabaclis (cultivated), 30 Jun 1970, Omawale & Persaud 118 (NY). **HONDURAS**. **Comayagua**: 75 mi. SW of Salitron, 15°00'58"N, 87°35'14"W, 15 May 1981, Meigs 1197 (BRIT). **JAMAICA**. **Manchester**: Marshall’s Pen, 2.25 map miles NW of Mandeville, 2300 ft, 8 Jun 1976, Thorne & Proctor 48066 (NY). **MARTINIQUE**: SaintPierre (cultivated), 1879, Père Duss 326 (NY). **MEXICO**. **Campeche**: Mpio. Calakmul, Zoh-Laguna, elev. 290 m, 18°35'39"N, 89°24'48"W, 19 May 1997 (MO). **Chiapas**: San Miguel Chimalapa, old road to Sta. María Chimalapa y Cofraclia, 16°42'20"N, 93°31'56"W, elev. 652 m, 14 Jun 2002, Miller et al. 323 (NY); **Colima**: Manzanillo, 1–31 Dec 1890, Palmer 998 (GH, NY); **Guerrero**: Mun. Eduardo Neri (Zumpango del Río), Amyaltepec, between there and Xalitla, Puerto el Rancho, hill E of intersection of roads to Amyaltepec, Xalitle, San Juan, toward río Tepecuacuilco, 17°58'00"N, 99°31'50"W, elev. 900 m, 4 Oct 2001, Amith & Hall 241 (NY); **Jalisco**: Chamela: Mpio. La Huerta, Estación de Biología, Chamela (UNAM), 12 Dec 1982, Bullock 1061 (MO); San Francisco de Ixcatán, Paso de Guadalupe (cult. from wild-collected seed), elev. 934 m, 20°50'16"N, 103°19'37"W, 23 May 2002, Miller et al. 275 (NY); **México**: Dto. Temascaltepec, Tejupilco, elev. 1340 m, 27 Jan 1933, Hinton 3217 (A, NY); **Michoacán**: Mun. Apatzingan, Chiquihuitillo, ca. 5 km SE of Apatzingan along road to Nueva Italia (cultivated), 257 m, 19°00'31"N, 102°20'16"W, 2 Jun 2002, Miller & Avila-Días 306 (NY); **Nayarit**: Mun. Tepic, Guadalajara-Mazatlán highway, elev. 632 m, 21°37'02"N, 104°58'03"W, 24 May 2002, Miller et al. 279 (MO, NY); **Oaxaca**: Mpio. San Miguel Chimalapa, old trail to Sta. Maria Chimalapa y Coraclia (cultivated), 16°42'52"N, 94°14'53"W, elev. 125 m, 13 Jun 2002, Miller et al. 322; **Sinaloa**: Culiacán and vicinity, Laguna Colorado, 21 Oct 1944, H. Gentry 7108 (GH, NY); **Sonora**: Quirosoba, Río Fuerte 17 Mar 1935, Gentry 1435 (A); **Veracruz**: km 28 of Tuxtepec-Valle Nacional road (cultivated), elev. 684 m, 17°52'37"N, 96°12'04"W, 10 Jun 2002, Miller et al. 314 (NY). **NICARAGUA**. **Boaco**: N slope of Cerro Mombachito and adjacent plain, between Cerro and main road (Boaco-Camoapa), ca. 12°24'N, 85°32'W, 8 Oct 1979, Stevens & Grijalva 14724 (NY). **PANAMA**. **Bocas del Toro**: Punta Peña (cultivated), 8°54'52"N, 82°11'10"W, elev. 90 m, 30 Aug 2001, Miller et al. 258 (NY). **Darién**: Mamey Village, 8 Mar 1982, Whitefoord & Eddy 434 (BM). **PERU**. **Cusco**: La Convención, Dist. Echarate, Papelpata, 12°45'45"S, 72°35'03"W, elev. 320 m, 28 Feb 2008, Suclli et al. 2926 (MO); **Loreto**: Yurimaguas, lower río Huallaga, elev. 135 m, 22 Aug–9 Sep 1929, Killip & Smith 27661 (NY); **Cajamarca**: San Ignacio, San Martín del Chinchipe, 5°19'16"S, 78°41'55"W, elev. 1000 m, 15 Sep 1999, Flores et al. 151 (MO); **Cusco**: La Convención, Dist. Huayopata, Abra de Málaga, 13°08'20"S, 72°18'16"W, elev. 370 m, 3 Dec 2003, Valenzuela et al. 2457 (NY); **San Martín**: Pongo de Cainarachi, río Cainarachi, tributary of río Huallaga, elev. 230 m, Sep–Oct 1932, Klug 2610 (A, NY). **PUERTO RICO**: Mun. Ciales, Reserva Tres Picachos, N of Road 533 at km marker 3.5, 18.232705° N, 66.540775°W, elev. 630 m, 20 Aug 2008, Atha et al. 6690 (NY). **ST THOMAS**: Bluebeard’s Castle (cultivated), 1–9 Mar 1924, Britton & Britton 218 (NY). **TRINIDAD**: Tahaquite, 30 Oct 1918, Broadway s.n. **UNITED STATES**. **Florida**. Miami: in pinelands, 1–30 Nov 1904, Small 2283 (NY). **VENEZUELA**. **Aragua**: Tovar, valley of Macarao, elev. 1000 m, 1854–55, Fendler 1308 (GH); **D.F.**: Caracas and vicinity, elev. 3000–3500 ft., 20 Jan 1921, Bailey & Bailey s.n. (NY).

#### Conservation status.

Although this species often occurs in dry forests and although local populations may be threatened, we consider this species to be of Least Concern because it has a relatively broad range, moreover it is widely cultivated.

#### Discussion.

*Spondias purpurea* is the most distinctive of the species occurring in the Neotropics. It is strictly dioecious and often ramiflorous, the inflorescence a pseudoracemose panicle or botryoid (versus a much-branched panicle), the calyx red, the petals petals red to purple (yellow in one cultivar), spreading to suberect at anthesis (vs. white to cream to greenish-yellow and reflexed), the disk often pink, and styles much less than half the length of the pistil. Moreover, it usually flowers before (not during or after) leaf flush, the sepals are slightly imbricate at base and rotund to ovate vs. apert and deltate or less often triangular, and the stigmas are slightly introrse to capitate (vs. extrorse) as the ovary develops.

### Spondias radlkoferi

Taxon classificationPlantaeSapindalesAnacardiaceae

Donn. Sm., Bot. Gaz. (Crawfordsville) 6: 194. 1891.

**Figure 23.**
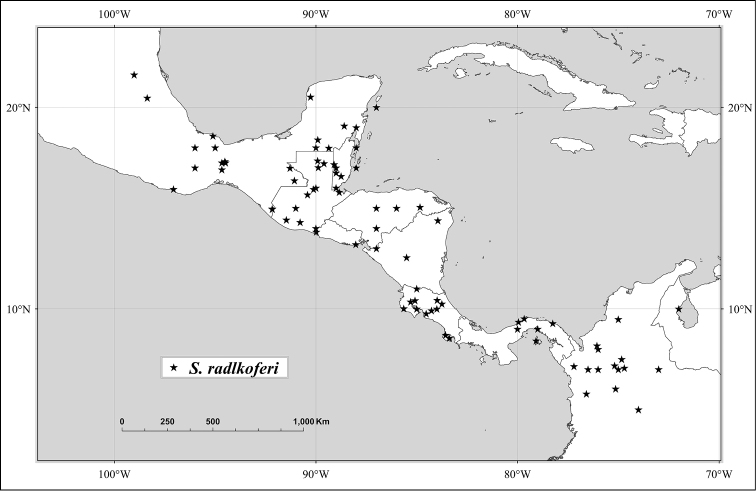
Distribution of *Spondias radlkoferi*.

Figs 2 , 5 , 15 , 23

Spondias nigrescens Pittier, Contr. U.S. Natl. Herb. 18: 75, Fig. 82. 1914.
Spondias radlkoferi Type. Costa Rica. Nicoya, May 1900, A. Tonduz 13925 (holotype: US-861287!; isotypes: GH!, K! (2 sheets), MO! (fragment)).

#### Type.

GUATEMALA. Esquintla [Escuintla]: Esquintla, Apr 1890, J. Donnell Smith 2087 (lectotype: US-1381173!, here designated; isolectotypes: GH!, K!, US-1381174!).

#### Description.

*Hermaphroditic trees*, reproductive height 5–30 m. Trunk 2.5–60 (90) cm diam.; *outer bark* (brownish) gray, smooth or occasionally rough, with shallow longitudinal fissures, sometimes with colums of warts; *inner bark* (brownish) red with white striations. *Trichomes* of three types: (1) curved (rarely flexuous), erect or appressed whitish hairs 0.3–0.6 mm long; (2) erect, fine, sharp white bristles to 0.05 mm long; and (3) erect, thick, blunt hairs to 0.05 (0.1) mm long. *Leaves* sometimes facultatively deciduous, 4–14-jugate, 13–58 cm long; petiole 3.2–11.5 cm long, petiole and rachis glabrous or more often with dense curved hairs; lateral petiolules 2–13 mm long, the terminal one 10–20 mm long, petiolules with dense curved hairs, less often glabrous except for scattered to sparse shorter curved hairs to 1 mm long (also on rachis between leaflets); basal leaflets 1.5–6.5 × 1.1–3.1 cm, other laterals (2.5) 3–13.6 × 1.6–6 cm, all laterals medially asymmetric, acroscopic side semi-ovate to semi-lanceolate, basiscopic side (narrowly) semi-elliptic; terminal leaflet 3.6–8.5 × 1.8–3 cm, slightly (ob)ovate to oblanceolate or rarely elliptic; apex either (1) obtuse, rounded, or retuse or sometimes broadly short-acuminate (populations in Petén, Veracruz, parts of Oaxaca), or (2) abruptly and narrowly long-acuminate (remainder of range), the acumen 3–14 mm long, often mucronate; lateral lamina usually basally asymmetrical, the acroscopic side obtuse to slightly cordate, the basiscopic side acute to attenuate, basal insertion asymmetrical; leaflet margin sometimes revolute, entire to slightly crenate, occasionally with a few concave-convex teeth, sometimes ciliate with sparse curved hairs; leaflets chartaceous to membranaceous, both surfaces dull. *Inflorescences* subterminal, developing along with new flush of leaves, 16–33 (60) cm long, 2.8–10 mm diam at base, flowers congested toward ends of axes, secondary axes 2–22 cm long, axes (sub)glabrous (populations in Veracruz, Petén, parts of Oaxaca) or with scattered bristles and usually with dense to sparse curved hairs (elsewhere); bracts on primary and secondary axes ca. 2.5–6 mm long, lorate to subulate, bracts on higher-order branches and bracteoles 0.3–0.5 mm long, ovate or deltate, semi-clasping, the apex acuminate, all bracts ciliate with curved hairs; pedicel 0.7–2.5 (3.5) mm long, portion distal to articulation 0.4–1 (0.9) mm long, glabrous or pubescent as on inflorescence axes (but curved hairs only to 0.2 mm). *Calyx* 0.5–0.6 mm long overall, aestivation apert or slightly imbricate, divided nearly to base, the lobes 0.4–0.5 mm long, broadly rounded-ovate, glabrous or with sparse bristles, the margin ciliate with bristles or short blunt hairs; petals 1.8–2.2 × 0.8–0.9 mm, oblong-elliptic, apex acute to slightly acuminate, variously reported as white, greenish yellow, rose-white, or greenish cream, glabrous, reflexed at anthesis; stamens spreading, antesepalous and antepetalous ones 1.8–2.2 and 1.5–1.6 mm long, respectively, the anthers 0.5–0.6 mm long, in dorsiventral view oblong, in lateral view oblong(-elliptic); disk 0.4–0.6 mm tall, 0.4–0.7 mm thick, summit slightly undulate and outer margin sulcate, dark purple (Wendt et al. 316, NY), surface markedly papillate; pistil 0.4–0.8 mm long, depressed-globose overall, divided nearly to base into subulate, apically slightly divergent styles, often with a few hairs to 0.2 mm long, the stigmas extrorse and broadly vertically oblong. *Fruits* 2.2–4 × 1.5–2 cm (dry), oblong to slightly obovoid, the apex distinctly umbonate (dry), sometimes oblique at base, orange to green when ripe, surface dull, not lenticellate but sometimes warty. *Seedlings* (from Garwood 2009): cotyledons ligulate, entire; first two eophylls opposite and trifoliolate, then alternate, the leaflets ovate and sparsely but regularly toothed; petiole, petiolules, midvein, and margin with dense thin stiff hairs.

*Leaflet venation*: Intramarginal vein present or sometimes appearing to have a marginal secondary, occasionally hidden by revolute margin. Secondary veins in 5–12 pairs, often arcuate but straight near base, spacing decreasing toward apex, the angle almost uniform but decreasing toward apex; insertion on midvein abruptly decurrent or less often excurrent; some inter-secondaries present, 0–1 per pair of secondaries and usually perpendicular to the midvein, long and reticulating or basiflexed; epimedial tertiaries present, short, parallel to secondaries or perpendicular to midvein, reticulating; intercostal tertiaries alternate-percurrent and irregular-reticulate with some admedial branching; quaternaries irregular-reticulate and freely ramified, areolation at tertiary or quaternary ranks, FEVs 2–3-branched, dendritic, terminating in highly branched sclereids; on abaxial side the midvein and secondaries prominulous to prominent and discolorous, higher-order veins flat to prominulous, on adaxial surface the midvein narrowly prominulous and the secondaries and higher-order veins flattened to slightly impressed, on both sides the midvein and secondary veins often with dense to scattered curved hairs, rest of surfaces glabrous or with sparse to scattered hairs, glabrescent with age except abaxial surface usually with hairy-tuft domatia in the axils of secondary veins.

#### Distribution.

*Spondias radlkoferi* has been recorded from Mexico (Mexico State) S to NW Colombia and Venezuela (Zulia), with one record from Los Ríos in Ecuador. As noted above, different leaflet forms are associated with different parts of its range.

#### Ecology.

This species is rather versatile ecologically, growing in primary to secondary formations or even roadsides, in seasonally dry tropical forest, tall evergreen forest, and pluvial forest, on limestone, black clay, and reddish brown stony soils. It occurs on slopes and in valleys at elevations ranging from 10–1000 m.

Given this species’ relatively broad distribution, its known phenology is broken down by region. Mexico: flowering Mar-May, fruiting May-Dec; Central America: flowering Dec-Jul, fruiting May-Jan.

In central Panama, populations of *Spondias radlkoferi* often flower 4–6 weeks later than *Spondias mombin* populations (Croat 1974a). The species is known to flower Apr-Jul (peaking in May-Jun) and to fruit Sep-Dec with a peak in Oct-Nov. Croat and others have suggested the fruit becomes an important source of food for mammalian species in times of food scarcity (Croat 1974a, b) and/or forest fragmentation (spider monkeys; Chaves et al. 2012).

As with the other *Spondias* species whose dispersal has been documented, the fruits of *Spondias radlkoferi* are often dispersed by frugivorous bats (Bonaccorso 1978, Bonaccorso and Humphrey 1984, Medellín and Gaona 1991); the endocarps of this species accounted for 50.5% of diaspores collected beneath the leaf tents of the tent-making bat *Artibeus watsoni* (Melo et al. 2009).

#### Common names.

Belize: hog plum, ho-bo (Arvigo 899, NY), pook (Maya, Balick 1824, GH, NY), rum-p’ok (Kekchi Maya, Arvigo 627, NY); Honduras: jovo (Hagen & Hagen 1096, NY), ciruela monte (Hagen & Hagen 1259, NY); Salvador: jocote (verde)(Rosales 394, NY).

#### Economic botany.

References to the economic botany of *Spondias radlkoferi* in the literature are scarce, because the species was treated as a synonym of *Spondias mombin* in the *Flora of Panama* (Blackwell and Dodson 1967) and the Flora of Ecuador (Barfod 1987), so some of the uses ascribed to *Spondias mombin* in these references should be applied to *Spondias radlkoferi*.

According to herbarium specimen data from Belize, the fruits of this species are edible; the bark is used to treat diarrhea, skin rashes, and fevers; a decoction is used as a mouthwash (*Arvigo 899*, NY); the leaves are boiled and drunk for bladder infections; a drink is prepared from the bark for internal bruises (*Arvigo 825*, NY); a tea from roots bark and buds is used to treat diarrhea; a tea from roots, bark, and buds is used to treat gonorrhea; boiled roots, bark and buds are used as an eye-wash; leaves and bark are boiled to make a tonic bath during pregnancy; and an infusion of leaves is used as a gargle for sore throats and to treat skin sores (*Balick 1824*, GH, NY).

#### Selected specimens examined.

**BELIZE**. **Belize District**: South of Yalbac Hills, Terra Nova Medicinal Plant Reserve, 17°21'N, 89°55'W, elev. 40 m, 19 July 1995, Walker et al. 1497 (NY); **Toledo District**: Bladen Watershed, Quebrada de Oro tributary, 16°35'N, 88°45'W, 16 Mar 1988, Brokaw 42 (NY). **COLOMBIA**: **Antioquia**: Mun. Dabeiba, km 4 Dabeiba Chigorodo road, 30 Jul 1987, Callejas et al. 4767 (MO, NY); **Caldas**: Mpio. Norcasia, Magdalena Medio, Hacienda Playa Alta, 260 m, 22 Jul 2001, Garzón & Lopera 103 (NY); **Chocó**: km 41–56 on QuibdóBolívar road, 5°47'N, 76°35'W, 11 Jun 1982, Gentry & Brand 36710 (NY); **Córdoba**: Mpio. Tierralta, installations of Urrá Dam, elev. 260 m, 9 Jun 2003, Fonnegra-G. et al. 7850 (NY). **COSTA RICA**. **Alajuela**: La Garita Dam, 25 Jul 1967, Lent 1148 (NY); **Heredia**: La Selva (OTS Field Station), on Río Puerto Viejo just E of confluence with Río Sarapiqui, elev. ca. 100 m, 8 Jun 1985, Jacobs 3293 (GH, BM, NY); **Puntarenas**: km 15 RincónPuerto Jiménez road, 8°33'N, 83°23'W, 4 Mar 1985, Croat & Grayum 59795 (MO, NY). **ECUADOR**. **Los Rios**: Vinces, Jauneche forest, km 70 Quevedo-Palenque, via Mocachi, 1°16'S, 79°42'W, elev. 70 m, (no date), Dodson et al. 8837 (GUAY, MO, SEL). **EL SALVADOR**. **Dept. Ahuachapán**, San Francisco Menéndez, El Corozo [Coroso], Mariposario, 13°49'N, 89°59'W, elev. 380 m, 24 Mar 2000, Rosales 394 (NY). **GUATEMALA**. **Alta Verapaz**: SE of Finca Yalpemech, near Alto Verapaz-Petén boundary, 23 Mar 1942, Steyermark 45214 (TEX); **Petén**: road to Melchior, 10 Jul 1965, Aguilar 37 (NY); Tikal National Park, Tikal, near airfield, 20 Aug 1959, Contreras 85 (GH, TEX). **HONDURAS**. **Atlantida**: Lancetilla Valley, near Tela, elev. 20–600 m, 6 Oct–20 Mar 1928, Standley 54022 (A); **Yoro**: Subirana, Oct 1937, C. von Hagen & W. von Hagen 1096 (NY); **MEXICO**. **Campeche**: Tuxpeña, 2 Nov 1931, Lundell 894 (GH, NY); Mpio. Calakmul, km 17 S of gate house for entry to Calakmul, 18°23'29"N, 89°54'W, 27 Jul 1998, Madrid et al. 1264 (MO); **Chiapas**: Mpio. Ocosingo, 0.2 km W of Nuevo Guerrero, 16°59'11"N, 91°17'14"W, elev. 210 m, 8 May 2002, Calónico Soto et al. 23370 (TEX); **Quintana Roo**: km 5 Las PanterasMargarita Maza road, on short cut to Mérida, 5 Aug 1982, E. Cabrera & H. Cabrera 3331; **Vera Cruz**: Mpo. San Andrés Tuxtla, Estación de Biología Tropical Los Tuxtlas, 18°3436'N, 95°0409'W, 15 Apr 1975, Calzada 1813 (NY). **NICARAGUA**. **Boaco**: Comarca San Isidro, ca. 17 km N of Camoapa, ca. 12°33'N, 85°30'W, 17 Jul 1984, Estrada et al. 9 (MO). **Zelaya**: Awas Tingni, 40 km S of Waspán, 14°23'N, 83°57'W, elev. 20 m, 20 Mar 1971, Little 25273 (MO). **PANAMA**. **Canal Zone**: Barro Colorado Island, 18 Apr 1968, Croat 4929 (NY), **Colón**: Distrito Portobelo, banks of Río Guanche, elev. 100 m, 9°31'N, 79°40'W, 18 Jan 1995, Galdames & Guerra 1928 (NY); **San Blas**: Comarca de San Blas, Playón Chico, aqueduct trail, 9°17'N, 78°15'W, 11 Sep 1994, Herrera & Arosemena 1834 (NY). **VENEZUELA**. **Zulia**: Sierra de Perija, vicinity of Kasmera (Estación Biológica de la Universidad del Zulia), SW of Machiques, 25 Aug 1967, Steyermark & Fernández 99734 (NY).

#### Conservation status.

We consider this species to be of Least Concern because of its broad range and relatively large populations in Central America, S Mexico and Colombia, although the population(s) in the heavily deforested region of Zulia, Venezuela may be endangered.

#### Discussion.

*Spondias radlkoferi* most closely resembles *Spondias mombin* because of its usually densely fissured bark, leaves 3–14-jugate, the midvein of the leaflet usually prominent abaxially, and the petals glabrous abaxially. The former can be distinguished by the intramarginal secondary vein (sometimes (sub)marginal) (vs. always removed from the margin); the costal secondary veins usually distinctly arcuate with excurrent insertion on midvein, sometimes with hairy tuft domatia in the axils abaxially (vs. essentially straight to very slightly arcuate with decurrent insertion on midvein, without hairy tuft domatia); the pedicel 0.7–2.5 (3.5) mm long (vs. 2–4.5 mm); and the fruit maturing green (rarely orange), obovoid with abruptly short-acuminate apex (vs. maturing yellow or orange(-brown), oblong to ellipsoid to globose, apex rounded to truncate).

Moreover, the tertiary veins are alternate-percurrent and irregular-reticulate (vs. irregular-reticulate and/or admedially ramified); FEVs 1–2-branched, terminating in highly branched sclereids (vs. 3+-branched and not terminating in branched sclereids); on pedicel the portion distal to the articulation almost always shorter than basal portion (vs. distal portion longer); the sepals slightly imbricate at base (vs. calyx apert); the disk markedly papillate (vs. not), the pistil often with with a few trichomes to 0.2 mm long (vs. glabrous).

### Spondias testudinis

Taxon classificationPlantaeSapindalesAnacardiaceae

J. D. Mitch. & Daly, Brittonia 50: 447–451. 1998

**Figure 24.**
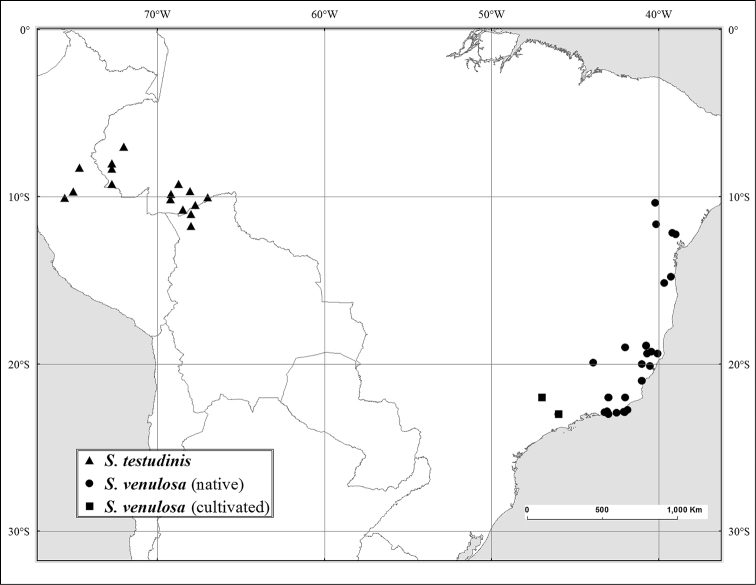
Distributions of *Spondias testudinis* and *Spondias venulosa*.

Figs 2 , 15 , 16 , 17 , 24

#### Type.

BRAZIL. Acre: Mun. Cruzeiro do Sul, Rio Juruá, left bank, Igarapé Viseu, 15 min. upstream by canoe, ca. 8°18'S, 72°44'W, 21 Mar 1992, D. C. Daly, J. Ramos, L. Ferreira & F. Walthier 7559. (Holotype: HUFAC!; isotypes: AAU!, BIOT!, INPA!, L!, MG!, MO!, NY!, RB!, US!).

#### Description.

*Hermaphroditic trees*, reproductive height 15–38 m. Trunk 35–65 cm diam.; *outer bark* grayishbrown, rough, shallowly to deeply fissured, with vertical stripes of raised lenticels; *inner bark* red and whitestriate. *Trichomes* of three types: white, flexuous to uncinate or suberect hairs to 0.3 (0.4) mm long (on leaves); capitate glandular hairs; and yellow, appressed to suberect, blunt glandular trichomes to 0.2 mm long (on inflorescences). *Leaves* (5) 7–13jugate, 20–33 cm long; petiole 3.1–5.6 cm long, petiole and rachis with dense flexuous to uncinate hairs; lateral petiolules 1–2 mm long, the terminal one 0.7–1.7 cm long, petiolules with hairs as on rachis; basal leaflets 2.9–3.8 × 1.5–2 cm, obliquely ovate to broadly elliptic, other laterals 5.2–7.6 × 1.6–2.3 cm, obliquely lanceolate to elliptic, terminal leaflet 5.5–5.9 × 1.4–1.8 cm, oblanceolate to elliptic; leaflet apex acuminate, acumen 4–13 mm, glandular-mucronate; lateral lamina medially and basally asymmetrical; acroscopic side rounded to obtuse, basiscopic side acute; basal insertion sometimes asymmetrical and slightly decurrent; leaflet margin often slightly revolute, subentire to sparsely serrate, the teeth concave-convex with acute (sometimes spiculate) apex; leaflets chartaceous to membranaceous, both surfaces dull *Inflorescences* (pseudo-)terminal, produced with new flush of leaves, ca. 9–17 cm long, secondary axes 5–8 cm long, axes with scattered to dense hairs and sparse to scattered appressed glandular hairs; bracts subtending secondary and higherorder axes 0.5–0.6 mm long, bracteoles 0.25–0.4 mm long, all bract(eole)s lanceolate to narrowly ovate, sparsely pubescent to glabrous, the margin with sparse glandular hairs; pedicel 1.5–1.7 mm long overall, portion distal to articulation 1.25–1.3 mm long, with scattered hairs. *Calyx* 0.6–0.9 mm long overall, aestivation apert, divided nearly to base, 0.4–0.6 mm long, deltate, margin entire to slightly erose; petals ca. 2–2.5 × 0.9–1.2 mm, narrowly ovate to elliptic, acute, greenish white, glabrous or with a few scattered hairs, reflexed at anthesis; stamens spreading, antesepalous and antepetalous ones 2.4–3 and 1.5–2.7 mm, respectively, the anthers 0.7–1.2 mm long, in dorsiventral view oblong-ovate, in lateral view oblong-elliptic; disk 0.75–0.8 mm tall, 0.15–0.2 mm thick, summit craggy and outer margin deeply sulcate, yellow; on recently opened flowers the pistil 1.3–1.6 mm long, cylindrical to slightly ovoid overall, divided ca. half its length into thickly subulate, apically divergent styles 0.6–0.8 mm long, the stigmas extrorse, obovate. *Fruits* (3.8) 4.9–6.3 × 2.3–2.7 (3.4) cm (dry), essentially oblong, apex truncate to rounded or slightly acuminate, base truncate; maturing dull (grayish) yellow to orangebrown, the surface (densely) raised-lenticellate, the endocarp 4.8–5.2 × 3–3.2 cm, oblong(-ellipsoid). *Seedlings* (based on Daly et al. 7251, NY): cotyledons linear, ca. 3.6 cm long; eophylls imparipinnate, 2–3jugate, leaflets of eophylls lanceolate, serrate, teeth laciniate.

*Leaflet venation*. Fimbrial vein absent; secondary veins 15–17 pairs, essentially straight, spacing somewhat irregular and decreasing toward base, angle acute and uniform, insertion on midvein abruptly decurrent; inter-secondaries present, ca. 1 per pair of successive secondaries, parallel to secondaries and more than 50% their length, usually with strong (often composite) admedial branching; some epimedial tertiaries present, parallel to secondaries; intercostal tertiaries irregular-reticulate and with (often composite) admedial branching; areoles poorly developed at tertiary and quaternary ranks, FEVs 5+-branched, dendritic, sometimes terminating in tracheoid idioblasts; marginal ultimate venation incompletely looped; on abaxial side the midvein prominent, secondaries prominulous; on adaxial side the midvein prominulous, secondaries flat to impressed, on both sides the midvein densely to sparsely pubescent, hairs scattered to sparse along remaining veins.

#### Distribution.

*Spondias testudinis* is endemic to southwestern Amazonia in Acre, Brazil and nearby portions of Bolivia (Pando) and Peru (Huánuco, Ucayali).

#### Ecology.

This species occurs in lowland dry to wet tropical forest on terra firme, elev. 200–780 m. It is known to flower in Sep-Oct and to fruit Feb-Jun.

#### Common names.

Brazil, Acre: cajá de jabotí (Daly et al. 7559, NY), cajarana (Cid Ferreira et al. 10116A, NY), cajarana da mata (Silveira et al. 475, NY), cajarana de anta (Figueiredo et al. 639, NY); Peru, Huánuco: ubos (Tello 354, NY); Ucayali: ubos colorado (Magín 112, NY), ushum (Vásquez & Jaramillo 10481, NY); Bolivia, Pando: casharana del monte (Jardim 717, MO). The fruits are eaten fresh or made into a juice; the juice is drunk sweetened, or added to a distilled alcoholic drink, or mixed with hot peppers to make a sauce (pers. obs.).

#### Selected specimens examined.

**BOLIVIA. Pando**: Manuripi, 35 km N of Puerto América, old well of Mobil, 11°44'S, 67°59'W, elev. 200 m, 13 May 1994, Jardim 717 (MO); Candelaria, km 36 Cobijaextremo Pando, 15 Jun 1978, Brig. Meneces 690 (INPA). **BRAZIL**. **Acre**: Mun. Brasiléia, Reserva Extrativista Chico Mendes, road to Seringal Porongaba, Colocação Santo Antônio, 30 km from Brasiléia, 25 May 1991, Cid Ferreira et al. 10116A (HUFAC, INPA, UFAC, NY); Mun. Cruzeiro do Sul, left bank of Rio Juruá, Igarapé Viseu, ca. 8°18'S, 72°44'W, 21 Mar 1992, Cid Ferreira et al. 10879 (HUFAC, NY); Mun. Senador Guiomard, Área de Estudos Florestais of FUNTAC, km 68 of BR-317 highway, 10°27'53"S, 67°44'30"W, 17 Mar 1997, Costello & Saraiva 25 (HUFAC, NY); Mun. Rio Branco, Reserva Florestal of EMBRAPA, km 14 of BR-364 (Rio Branco-Porto Velho) highway, 10 Jun 1997, Costello et al. 50 (HUFAC, NY); Mun. Bujari, Riozinho do Andirá, tributary of Rio Acre, approx. 9°39'S, 68°02'W, 12 Jun 1997, Costello & Saraiva 88 (HUFAC, NY); Mun. Xapuri, Rio Acre, 3 hrs downstream by boat downstream by boat from Xapuri, then 1 hr walking inland from left bank, 10°45'S, 68°28'W, 9 Nov 1991, Daly et al. 7251 (HUFAC, NY); Mun. Sena Madureira, basin of Rio Purus, Rio Macaua, below Colônia Barro Alto, 9°12'.48'S, 68°44.17'W, 4 Apr 1994, Daly et al. 8182 (INPA, NY); Mun. Marechal Taumaturgo, basin of Rio Juruá, Reserva Extrativista do Alto Juruá, Colocação Ceará, 9°12'S, 72°44'W, 5 Apr 1993, Silveira et al. 475 (CAS, CTES, HPA, HUFAC, INPA, MEXU, NY, RB). **PERU**. **Huánuco**: Prov. Puerto Inca, Dtto. Yuyapichis, Unidad Modelo de Manejo y Producción Forestal DANTAS, 9°40'S, 75°02'W, 1–15 Oct 1990, Tello 354, 396 (NY); **Pasco**: Prov. Oxapampa, trail between Pozuzo and Yanahuanca, 10°03'S, 75°33'W, 17 Mar 1984, Smith et al. 6406 (MO); **Ucayali**: Yarina Cocha, Nueva Esperanza de Panaillo, 8°15'S, 74°40'W, elev. 148 m, 1 Apr 1988, Vásquez & Jaramillo 10481 (MO, NY).

#### Conservation status.

We classify this species as of Least Concern; although it has a more limited geographic distribution than most of its congeners, it is relatively common where it occurs in SW Amazonia, and that region is still mostly forested.

#### Discussion.

*Spondias testudinis* closely resembles *Spondias macrocarpa* because both have leaves (5) 7–13-jugate, the leaf rachis densely pubescent; intersecondary veins sometimes present, parallel to secondaries and with admedial branching; anthers 0.7–0.9 mm long (to 1.2 mm in *Spondias testudinis*), and the disk much taller than thick. The former is distinguished by the following characteristics: leaflet teeth spiculate (vs. not), secondary veins 15–17 (vs. 10–15) pairs; pedicel 1.5–1.7 mm (vs. 2.5–3.5 mm) long; and fruits (3.8) 4.9–6.3 cm (vs. 3.5–4.2 cm) long.

It differs from the sympatric *Spondias mombin* by a number of features, including the following: trichomes on leaves flexuous to uncinate or sub-erect (vs. always straight), to 0.3 (0.4) mm (vs. to 0.2 mm); leaves (5) 7–13-jugate (vs. 3–7-jugate), the midvein on adaxial side sparsely or more often densely pubescent (vs. glabrous or sometimes with trichomes on midvein and secondary veins); fruit surface lenticellate (vs. smooth); eophylls on seedlings imparipinnate, 2–3jugate (vs. trifoliolate) and the eophyll leaflets lanceolate (vs. ovate) and laciniately (vs. simply acutely) serrate (*Spondias mombin* seedlings described and illustrated in Vogel 1980).

### Spondias tuberosa

Taxon classificationPlantaeSapindalesAnacardiaceae

Arruda in Koster, Trav. Brazil: 496. 1816.

Figs 2 , 13 , 15 , 16

#### Type.

BRAZIL. Bahia: Mun. Rio das Contas, km 7 Rio de ContasLivramento do Brumado road, 13°38'S, 41°50'W, 12 Dec 1988, R. M. Harley, B. Stannard, J. R. Pirani, A. Furlan & J. Prado 27127 (neotype: SPF!, here designated; iso-neotypes: CEPEC!, K!, NY!).

#### Description.

*Hermaphroditic (sometimes andromonoecious) trees or shrubs*, often deciduous for extended periods, reproductive size 2–10 m tall × 11.5–41 cm diam, with dense, low, tortuous branching, often the crown broader than tree height, often the outer branches forming a weeping habit, these sometimes rooting in the ground; short shoots often present and sometimes becoming spinose. *Roots* tuberous. *Outer bark* gray, frequently with wavy fissures, surface irregular but relatively smooth, apparently shed in rectangular plates. *Trichomes* of two types: (1) flexuous to curved white hairs to 0.6 mm long; and (2) erect to ascending hairs to 0.25 mm long. *Leaves* 1–4 (5)-jugate, 6–17 cm long; petiole 1.7–5 cm long, petiole and rachis glabrous or sometimes with sparse to scattered (rarely dense) flexuous hairs; lateral petiolules 0–4 mm long, sometimes reddish, the terminal one 0.3–1.4 cm long, petiolules with sparse to dense flexuous hairs; basal leaflets 2.5–5.1 × 1.5–3.2 cm, (broadly) elliptic to ovate, the other laterals 2–6.5 × 1.2–3.5 cm, (broadly) ovate or sometimes broadly elliptic, terminal leaflet 3–5.5 × 1.6–2.7 cm, obovate to (broadly) elliptic, leaflets often plicate; leaflet apex sharply but usually gradually acuminate (then the acumen 2–7 mm) or less often acute to obtuse or rounded, rarely emarginate, mucronate; lateral lamina usually medially and basally (sub)symmetrical, the base obtuse to subcordate, basal insertion usually (sub)symmetrical and excurrent, leaflets chartaceous to membranaceous, both surfaces dull; leaflet margin sometimes slightly revolute and thickened (sometimes red on juvenile leaflets), usually entire, occasionally 1–2 crenulations on a leaflet, densely ciliate with flexuous hairs to 0.4 mm long, especially on young leaflets. *Inflorescences* terminal, initiated with or before a new flush of leaves or sometimes on branchlets with mature leaves, 5.5–20 cm long, 1–1.4 mm diam near base, broadly branched, secondary axes to 4.8 cm long, the axes glabrous or more often provided with sparse erect to ascending hairs or with flexuous hairs, the latter to 1 mm long near base; bracts on axes 1–8.5 mm long, bracteoles to 0.6 (1.5) mm long, all bracts linear to lanceolate and ciliate, sometimes with ascending hairs abaxially; pedicel 2.2–3.5 mm long, portion distal to articulation 1.5–2.4 mm long, with hairs as on axes. *Calyx* (0.3) 0.4–0.7 mm long overall, aestivation apert, lobes (0.1) 0.25–0.35 mm long, (depressed-)deltate, glabrous or more often provided with pubescence as on pedicel and ciliate, the longer hairs sometimes stiff; petals (1.2) 2–2.7 × (0.65) 0.8–1.5 mm, lanceolate to almost elliptic, less often ovate, apex acute, white or cream, abaxial surface glabrous or provided with scattered stiff hairs to 0.25 mm long, petals (slightly) reflexed at anthesis; stamens spreading, antesepalous and antepetalous ones 1.5–2.1 and (1.35) 1.5–1.85 mm long, respectively, the anthers 0.5–0.9 mm long, in dorsiventral view elliptic, in lateral view oblong, yellow, sometimes the antesepalous ones larger than the antepetalous ones,the filaments white; disk 0.2–0.5 mm tall, 0.4 mm thick, summit markedly undulate and outer margin deeply sulcate, (greenish-)yellow, markedly papillate; pistil 0.65–1.1 mm long, subcylindrical overall, divided almost to base into subulate, apically connivent styles 0.45–0.5 mm long, the stigmas extrorse, vertically oblong. *Fruits* 2.6–3.3 × 1.5–2.2 cm (dry, see note below regarding *umbu-cajá*), obovoid to subglobose, the apex obtuse to truncate, often the widely separated style scars raised and still evident at maturity, the base usually truncate, sometimes substipitate, maturing (greenish to whitish) yellow, surface smooth and dull; mesocarp thick and fleshy; endocarps 2–2.5 × 1.3–2 cm, essentially obliquely obovoid but laterally compressed and very slightly 1-carinate, entirely enveloped by a smooth, skin-like layer that when peeled away reveals a smooth, hard inner layer with four small and one larger peri-apical, fiber-filled, circular pores and two smaller pores straddling the keel near the proximal end; fruits often 1-seeded.

*Leaflet venation*: Fimbrial vein absent; secondary veins in 10–20 pairs, straight, attenuate at both ends, spacing uniform, angle nearly perpendicular, insertion on midvein abruptly decurrent; intersecondaries and epimedial tertiaries absent; intercostal tertiaries with some irregular-reticulate veins but primarily composite-admedial departing from secondaries or the intramarginal vein, usually attenuate at both ends; quaternaries irregular-reticulate and freely ramified, areolation at tertiary and quaternary ranks, FEVs 3–4+-branched, dendritic, terminating in only slightly thickened tracheoid idioblasts (see Fig. 3, p. 253 in Silva 1973); marginal ultimate venation looped; on both surfaces the midvein narrowly prominulous (seldom prominent abaxially or flat adaxially), secondaries and higher-order veins flat to prominulous on both sides, sometimes impressed adaxially, the surface glabrous or when young with dense flexuous hairs, often glabrescent except hairs persisting on midvein and toward leaflet base; adaxial side glabrous or when young with scattered flexuous hairs, glabrescent with age.

#### Distribution.

The native range of *Spondias tuberosa* is from Maranhão E to Paraíba and south to Minas Gerais in Brazil; it is also cultivated in SE Brazil (see below).

#### Ecology.

This species is a constituent of the arid *caatinga* in NE Brazil, known to flower in Aug-Mar and fruit Oct-May (Machado et al. 1997, plus data from exsiccatae). Nadia et al. (2007) suggested that this species can be andromonoecious, and recent observations of the current senior author in Canudos, Bahia, Brazil indicate that this may be correct.

In Paraíba state, Brazil, two species of bee and one species of wasp were the principal pollinators of *Spondias tuberosa* flowers (Nadia et al. 2007). The fruits are dispersed principally by mammals (Griz and Machado 2001), notably collared peccaries (Olmos 1993); in anthropic landscapes, cattle can be important dispersal agents (Griz and Machado 2001).

#### Common names.

Brazil, Bahia: cajá do sertão (Arbo et al. 7239, GH, NY), imburana (V. Souza 329, NY), caya (Arbo et al. 5776, COL, GH, NY); Maranhão: umbu, umbuzeiro (Eiten & Eiten 10810, NY); Minas Gerais: imbu verdadeiro (Ratter et al. 2702, E), umbu (Andrade & Figueiredo 115, NY); Pernambuco: imburana de cambão (Costa 176, NY), imbuzeiro (Costa 17, NY).

#### Economic botany.

In the species’ native range, its fruits are often wild-collected or collected from managed (spared) trees (Popenoe 1948). It is cultivated in SE Brazil, propagated by cuttings or seeds in home gardens or occasionally in orchards (e.g., Lorenzi et al. 2006). It was illustrated in Piso and Markgraf’s (1648) *Historia naturalis brasiliae*, suggesting a long pre-Columbian history of use. The fruit pulp is used primarily to make a popular juice and ice cream, also to make a drink called *umbujada* made by boiling the pulp with milk, curds and sugar (Koster 1816, Popenoe 1948, both as *imbuzada*; Lins Neto et al. 2010). The fruits and roots are high in Vitamin C (Cavalcanti et al. 2000). The seeds have potential as a source of cooking oil due to the high oil content, high mineral concentration, and fatty acid composition (Borges et al. 2007). The roots have been used as a famine food in times of drought (Nascimento et al. 2012).

Like other species of the genus, *Spondias tuberosa* has several medicinal uses. In Bahia, the leaves have been used for medicinal baths and a tea of the bark is used to treat colds and dysentery (Mattos Silva 2301, NY).

The species could be a valuable addition to the economic flora and diet of many other dry tropical regions.

#### Selected specimens examined.

**BRAZIL**. **Bahia**: Mun. Uauá, Serra do Jerônimo, 9°43'23"S, 39°19'56"W, 30 Mar 2000, Alves et al. s.n. (ALCB 47955) (CEPEC); Mun. Andaraí, 33 km NE of Mucujé, toward Nova Redenção, approx. 12°49'S, 41°12'W, ca. 450 m, 25 Nov 1992, Arbo et al. 5776 (COL, GH, NY); Mun. Candeal, 8 km N of Tanquinho, trail to Ichu, elev. 200–300 m, approx. 11°54'S, 39°06'W, 15 Jan 1997, Arbo et al. 7239 (CEPEC, GH, NY); Mun. Iaçu, Fazenda Lapa, 12°42'S, 39°56'W, 26 Feb 1983, Bautista 727 (MG); Japirá, Vila do Barra, 1840, Blanchet 3078 (GH, NY, W)(syntype of S. venulosa); Quijingue, Serra das Candéias, 5 km W of Quixabá do Mandacaru village, 10°55'20"S, 39°04'59"W, 350–632 m, 13 Nov 2005, Cardoso et al. 876 (NY); Tucano, Pedra Grande village, road to Serra do Pai Miguel, 11°07'24"S, 38°46'25"W, elev. 224 m, 20 Dec 2007, Cardoso & Ferreira 2234 (NY); Riacho Congú, Cachoeira, valley of Paraguaçu and Jacuipe rivers, 12°32'S, 39°05'W, Nov 1980, Cavalo et al. 942 (NY); Novo Remanso, 9°17'S, 41°32'W, 520 m, (w/o date), Coradin et al. 5945 (K); Andorinha, road to pond, 10°12'44"S, 39°54'46"W, 430–470 m, 18 Feb 2006, França et al. 5464 (NY); road to Manoel Vitorino from Jequié, 14°00'S, 40°10'W, 9 Feb. 1985, Gentry & Zardini 49965 (MO, NY); Mun. Paramirim, km 10–16 ParamirimLivramento do Brumado road, 13°33'S, 42°12'W, 2 Dec 1988, Harley & Taylor 27064 (K, NY, SP); Mun. Rio de Contas, by road 11.5 km from Rio de Contas toward Marcolino Moura, 13°35'52"S, 41°45'22"W, 1 Nov 2004, Harley et al. 55198 (NY); Mun. Bom Jesús da Lapa, Rio das Rãs, elev. 450 m, 15 Nov 1991, Hatschbach et al. 55163 (US); Mun. Glória, Raso da Catarina [ca. 9°40'S, 38°40'W], 31 Jan 1982, Rocha et al. 790 (GUA); caatinga near Caldeirão, Oct 1906, Ule 7255 (K). **Ceará**: near Lavras da Mangabeira, povoado São Francisco, 30 Jan 1968, Carauta 552 (GUA, NY); **Espírito Santo**: pasture near corral of Sr. Wilson Machado, km 7, 15 Dec 1992, Folli 1757 (NY); **Maranhão**: Mun. Loreto, “Ilha de Balsas,” region between Balsas and Parnaíba rivers, ca. 35 km S of Loreto, 7°23'S, 45°05'W, elev. 300 m, 9 Feb 1970, Eiten & Eiten 10523 (K, NY), Loreto city, 1 Mar 1970, Eiten & Eiten 10810 (K, NY, US); **Minas Gerais**: Mun. Januária, district of Fabião, road to Abrigo Bichos, 15°00'–14°57'S, 44°24–30'W, 25 Oct 1997, Lombardi 2080 (NY); **Paraíba**: W of Campina Grande, between Sta. Luzia and Joazeiro (= Taperoá)[ca. 7°12–13'N, 35°52'–36°49'W], 8 Oct 1927, Ginzberger 1489 (WU); Mun. Barra de Santa Rosa, Fazenda Quandu, region of Curimatan, 30 Jan 1970, Souto 43 (RB); **Pernambuco**: Mun. Venturosa, Parque Pedra Furada, 8°34'30"S, 36°52'45"W, elev. 783 m, 28 Feb 1998, Costa 17 (NY), Bodocó, near town, 12 Feb 1991, Lisboa & Silva 4521 (MG); Paruaru [Caruaru] (cult.), 4 Nov 1931, D. B. Pickel 590 (GH); **Piauí**: Mun. Teresina, near Rio Paty, 4 Jul 1907, Ducke 799 (MG); São Miguel do Tapuio, 22 May 1979, Fernandes s.n. (EAC 6044)(NY); Mun. São Raimundo Nonato, Fundação Ruralista, +/- 8–10 km NNE of Curral Novo and 220 km ENE of Petrolina, ca. 9°00'S, 42°00'W, elev. 320 m, 22 Jan 1982, Lewis & Pearson 1154 (K); **Rio de Janeiro**: Jardim Botânico (cult.), 2 Oct 1939, Kuhlmann s.n. (RB 40595) (RB); Campos, Atafona (cultivated), Apr 1939, Sampaio 8219(R); **São Paulo**: Cajuru, Fazenda Sta. Carlota (cult.?), 13 Nov 1986, Bernacci 174 (SPF). **UNITED STATES**. **Florida**: Miami-Dade County, Homestead, 611 West Pierce Ave. (cult.), elev. 10 m, 3 May 1928, Fisher s.n. (BRIT).

#### Conservation status.

We consider this species to be of Least Concern. It is broadly distributed in the *caatinga* vegetation of NE Brazil, and even where its habitats are highly disturbed, it is spared because of its highly prized fruits. Moreover, it is cultivated both within and beyond its range. On the other hand, one must consider the possibility that native populations are declining due to disturbance.

#### Discussion.

This species first appeared in print (as *Spondias tuberosa*) in the appendix of Koster’s *Travels in Brazil* (1816), in which Koster translated text and a large number of nomina nuda ascribed to “Arrud. Cent. Plant. Pern.” These referred to Manuel Arruda Câmara’s *Centuriae plantarum pernambucensium*, which was never published but formed the foundation for Pinto’s (1873) *Diccionario de Botanica Brasileira* (see summary in Kirkbride 2007, also Britten 1896). Although no type exists, the plant’s distribution, common names, and uses leave no doubt as to its identity, so a neotype has been selected.

The circumscription of *Spondias tuberosa* is complicated by the occurrence in Bahia of an entity – most or all of whose individuals are cultivated – that is recognized by local people as distinct enough to merit a different common name, *umbu-cajá* or *cajá-umbu*. The fruit is indeed distinct: the pyrene is larger (2.7–2.8 × 1.8–1.9 cm vs. 2–2.5 × 1.3–2 cm); more significantly, the stony endocarp is overlain by a thinner fibrous matrix, moreover the five peri-apical pores are subequal in size (vs. four small and one larger), larger than in *Spondias tuberosa*, and oblong (vs. circular), and the pyrene has four (vs. 1) keels or trabeculae (beams)(based on *Mattos Silva et al. 2299*, NY). The fresh fruit is ca. 4–4.7 × 3.2–3.4 cm and the pyrene 2.7–2.8 × 1.8–1.9 cm (Lorenzi et al. 2006).

On the other hand, the vegetative and floral morphology of the material referred to *umbu-cajá* is almost indistinguishable from those of *Spondias tuberosa*, although the leaflets tend to be larger and more broadly ovate, and the apex to be narrowly acuminate. The revolute leaflet base with rather dense long hairs more closely resembles that of *Spondias venulosa*. We are not aware of any differences in habit or in bark morphology.

The fruits of *umbu-cajá* are consistently obovoid, but this is within the range of variation of *Spondias tuberosa* s.s.; the fruit surface is sparsely lenticellate, which we have not observed in *Spondias tuberosa* s.s. (Marlon Marchado, pers. comm., 4/2013).

In his book on Brazilian fruits, Lorenzi et al. (2006) treated *umbu-cajá* (e.g., *Lorenzi 6074*, NY) as a separate entity from *Spondias tuberosa*, observing that it produces only sterile seeds, that it is propagated only by cuttings, and that it is known only in cultivation in Bahia, Alagoas, and Pernambuco states.

Silva Júnior et al. (2004) suggested that *umbu-cajá* is a hybrid between *Spondias tuberosa* and *Spondias mombin*, but Almeida et al. (2007) examined karyology and genomic *in situ* hybridization of *Spondias* spp. and concluded that the *umbu-cajá* (1) is not of hybrid origin and (2) is distinct from both putative parents. In the present treatment, this entity is considered a variant of *Spondias tuberosa* unless and until further genetic/molecular investigations suggest otherwise.

Several collections can be referred to umbu-cajá. Brazil. Bahia: Mun. Itabuna, city center, 2 Apr 1998, Carvalho & Kersul 6493 (cajá-umbu, NY); Mun. Cruz das Almas, cultivated at EMBRAPA, 14 Feb 2006, Lorenzi 6074 (E, HPL, MO, NY); Mun. Maracás, Fazenda Tanquinho (entrance at km 23 of MaracásPalnaltino road), 3 Mar 1988, Mattos Silva et al. 2299 (cajá-imbu, CEPEC, NY), 3 Mar 1988, Mattos Silva et al. 2301 (umbu-cajá, CEPEC, NY).

### Spondias venulosa

Taxon classificationPlantaeSapindalesAnacardiaceae

(Mart. ex Engl.) Engl. in A. DC. & C. DC., Monogr. phan. 4: 245. 1883

Figs 2 , 6 , 15 , 16 , 24

Spondias purpurea var. venulosa Mart. ex Engl. in Mart., Fl. bras. 12(2): 373. 1876.
Spondias venulosa Type. Based on Spondias venulosa (Mart. Ex Engl.) Engl.
Spondias myrobalanus sensu Vell., non L., Fl. flumin. 4: 197, t. 185. 1825[1829].

#### Type.

BRAZIL. Rio de Janeiro: Rio de Janeiro (cultivated), November (w/o year), Martius Obs. 274 (lectotype: M, n.v., here designated; F-photo!; G-photo!; NY-photo!).

#### Description.

*Hermaphroditic trees*, reproductive height 5–30 m. *Trunk* 11–92 cm diam., *outer bark* (brownish) gray, rough, sparsely to densely fissured, the fissures broad, deep and wavy, shed in long, thick, irregular plates; *inner bark* soft and whitish with beige to pale red striations. *Trichomes* white to yellowish, erect to flexuous, 0.4–0.6 (0.8) mm long. *Leaves* 3–5jugate, 12.3–23 cm long, the petiole, rachis and midvein often red when young; petiole 5.3–7 cm long, petiole and rachis glabrous; lateral leaflets subsessile or the petiolules to 3 mm long, the terminal one 7–15 mm long, petiolules pubescent; basal leaflets 2.2–7 × 1–3.5 cm, ovate to broadly elliptic, other laterals 3–8 × 1.2–4.2 cm, usually (broadly) elliptic or ovate or less often lanceolate, terminal leaflet 2.8–8.7 × 1.1–4.2 cm, elliptic to obovate; leaflet apex abruptly and narrowly acuminate, the acumen 3–16 mm long, the apex tip sharply acuminate; lateral lamina often medially strongly asymmetrical and then the acroscopic side ovate and basiscopic side elliptic, base subsymmetric or asymmetric, the acroscopic side cuneate to cordate, the basiscopic side acute to rounded, basal insertion often slightly asymmetric, often decurrent; leaflet margin usually revolute, strongly so at base, entire or occasionally serrulate, with blunt convex-convex teeth; leaflets chartaceous to coriaceous, often both surfaces glossy (mature leaflets); intramarginal vein submarginal. *Inflorescences* subterminal and terminal, developing with leaf flush, (3.5) 7–19.5 long, 1.3–2.3 mm diam near base, broadly branched, secondary axes 0.5–5 cm long; bracts on primary and secondary axes 0.6–2.4 mm long, lanceolate to lorate and acuminate, bracteoles 0.4–0.6 mm long, ovate to subulate and often semi-clasping; pedicel 1.8–3.5 mm long overall, portion distal to the articulation 1.5–2.7 mm long. *Calyx* 0.7–1.4 mm long overall, aestivation apert, lobes 0.4–0.8 mm long, deltate to triangular, the margin erose; petals 1.8–2 × 0.7–1 mm, lanceolate to deltate, slightly acuminate, white to yellowish, reflexed at anthesis; stamens inflexed, antesepalous and antepetalous ones 1.2–1.3 and 0.9–1.1 mm long, respectively, the anthers 0.4–0.5 mm long, elliptic in both dorsiventral and lateral views; disk 0.35–0.4 mm tall, 0.3 mm thick, summit undulate and outer margin sulcate, yellow; pistil 1–1.2 mm long, thickly subcylindrical overall, divided nearly to base into subulate, apically connivent styles 0.5–0.7 mm long, the stigmas extrorse, obovate. *Fruits* 3.6–6 × 1.9–3.6 cm (dry; when fresh 4–8 × 3.2–3.8 cm), (slightly) oblong(-obovoid) to slightly (ob)ovoid, often lumpy, apex rounded, truncate, or sometimes acuminate, base obtuse to truncate, maturing yellow and glossy, surface shallowly pitted and very sparsely lenticellate, the lenticels flat; endocarp usually slightly obovoid and usually acuminate.

*Leaflet venation*: Secondary veins 10–20, straight, spacing uniform, angle slightly acute and decreasing toward apex, insertion decurrent; intercostal tertiaries few per pair of successive secondaries, usually arising from near intramarginal vein, with (usually composite) admedial branching; areolation at tertiary rank, quaternaries freely ramified; FEVs 4+-branched, dendritic, terminating in tracheoid idioblasts; marginal ultimate venation mostly looped; on abaxial side all venation narrowly prominent, pubescent on revolute base (sometimes extending onto midvein), usually glabrescent; on adaxial side all venation flat or more often broadly prominulous but fluted, veins often discolorous (drying blackish).

#### Distribution.

*Spondias venulosa* ranges from eastcentral Bahia to southern Rio de Janeiro and extreme southeastern Minas Gerais; cultivated as far south as Campinas and São Paulo city in São Paulo state.

#### Ecology.

This species occurs in moist upland forests of the Mata Atlântica Complex, also also in the *tabuleiro* forests that occur on red-yellow dystrophic podzols in the low, flat, subcoastal tablelands of Espírito Santo.

There is no literature or herbarium label data that shed light on the pollination or dispersers of this species. It is known to flower in Aug-Feb and fruit Jan-Sep.

#### Common names.

Brazil, Espírito Santo: taipá (*Spada 51*, NY), cajá (*Folli 1608*, NY).

#### Economic botany.

The fruits of *Spondias venulosa* are edible and occasionally used to make juices; most fruits are wild-collected (Lorenzi et al. 2006). The species is not widely planted in home gardens; it is found in some Brazilian parks, botanical gardens and arboreta, e.g., Belo Horizonte (*Macedo 5451*, US), Rio de Janeiro (pers. obs. and *Kuhlmann s.n. (GUA 11051)*, GUA), Itabuna (Bahia, *Hage 230*, NY), and São Paulo (*Gehrt [Hatschbach] s.n. (SP 39886)*, NY, SP).

#### Selected specimens examined.

**BRAZIL. Bahia**: Mun. Senhor do Bonfim, Povoado de Estiva, Serra de Santana, 10°21'57"S, 40°11'51"W, 689 m, 13 July 2005, Cardoso et al. 716 (NY); Anguera, Fazenda Retiro, ca. 18 km from Feira de Santana, on Feijão-Ipirá road, 12°09'42"S, 39°11'02"W, elev. 300–600 m, 22 May 2007, Cardoso & Santos 1935 (NY); Mun. Mairi, km 41 Capim Grosso-Mairi road, 11°39'S, 40°08'W, elev. 460 m, 21 Sep 1996, Pereira-Silva et al. 3638 (NY); Mun. Feira de Santana, Campus of Universidade Estadual de Feira de Santana, 12°15'S, 38°58'W, 31 Jan 1992, Queiroz 2604 (NY); Mun. Itajú da Colônia, 7.5 km SE of Itajú do Colônia on road to Palmira, 15°09'13.1"S, 39°39'27.6"W, elev. 250 m, 19 Mar 2001, Thomas et al. 12363 (MO, NY); **Espírito Santo**: Mun. Brejal, 15 km N of Colatina, dirt path along left bank of rio Pancas, approx. 19°23'S, 40°41'W, 28 Jan 1997, Arbo et al. 7777 (CEPEC, GH); Águas Claras, Escola Agroecológica, 18°53'32"S, 40°43'48"W, elev. 300–500 m, 6 Jun 2006, Demuner et al. 2382 (NY); Rio Bananal, Alto Bananal, property of Jonas Graci, 19°14'56"S, 40°24'59"W, elev. 300-600 m, 25 Apr 2007, Demuner et al. 3783 (NY); Reserva Vale (BR-101 Norte, km 122), Estrada Flamengo, 19°07'14"N, 30°54'59"W, 1 Mar 2011, Stefano et al. 201 (NY, RB); **Minas Gerais**: Aimorés, km 15.5 of BR-259 highway, in pasture, 18 Oct 2004, Luz 248 (CVRD, NY); Belo Horizonte, Praça Benjamin Guimarães, no cruzamento com avenidas Getúlio Vargas, e Afonso Pena, 15 Sep 1988, Macedo 5451 (US)(cultivated); Parque Florestal Rio Doce, 2 Nov 1992, Stehmann s.n. (BHCB 20855) (BHCB, NY); **Rio de Janeiro**: Quinta da Boa Vista, 22 Oct 1930, Brade s.n. (R 73762)(R); Mun. Armação dos Búzios, Fazenda Caravelas, S slope between Peró and Caravelas beaches, 4 May 2000, Farney et al. 4083 (NY, RB); Rio de Janeiro, w/o date, Gaudichaud 826 (P; syntype of S. venulosa); Rio de Janeiro, Morro do Inglez [Inglês], Corcovado, 22 Aug 1886, Glaziou 827 (K, P; syntype of S. venulosa); Mun. São Pedro d’Aldeia, Morro de Sapiatiba [Serra de Sepetiba], elev. 200–400 m, 10 Sep 1987, Leitman et al. 284 (NY, RB); Mun. Cabo Frio, new road to Búzios, Baia Formosa, entrance to Capão da Pedra, Fazenda of Sr. Henrique Massala, 6 May 1987, Lima 2883 (NY, RB); Mun. Niterói, road to Itaipu, near entrance to Itacoatiara, base of slope of Serra da Tiririca, 25 Sep 1990, Lima et al. 3988 (NY, RB); Sete Pontes, 9 Feb 1876, Rohan 43 (R 73728)(R); Rio de Janeiro, 1816-1821, St.-Hilaire 1026 (K, P); Niterói, Pico do Alto Mourão [Moirão], between Niterói and Maricá, 6 Aug 1991, Santim [Santin?] et al. s.n. (RB 300478) (NY, RB); Mun. Niterói, between Campos and Morro do Côco, 8 Sep 1964, Trinta & Fromm 1050 (NY); **São Paulo**: São Paulo, 29 Nov 1938, Gehrt [Hatschbach] s.n. (SP 39886) (NY, SP) (cultivated); Campinas, 18 Nov 1936, Hoehne & Gehrt [Hatschbach] s.n. (SP 36835)(NY) (cultivated).

#### Conservation status.

We consider this species to be of Least Concern. It is rather widespread in the Atlantic Coastal Forest of Brazil, and despite severe fragmentation of that region’s lowland forests, it appears to thrive in even rather small forest fragments.

#### Discussion.

The similarities and differences between this species and *Spondias admirabilis* are discussed under the latter species.

### Excluded taxa

*Spondias acida* Solander ex Benth., Fl. austral 1: 492. 1863 non Bl. = *Pleiogynium timoriense* (DC.) Leenh.

*Spondias angolensis* O.Hoffm., Linnaea 43: 125. 1881. = *Pseudospondias microcarpa* (A.Rich.) Engl.

*Spondias axillaris* Roxb., Hort. bengal. 34. 1814. = *Choerospondias axillaris* (Roxb.) Burtt & Hill.

*Spondias birrea* A.Rich. in Guill. & Perr., Fl. Seneg. tent. 152. t. 41. 1830-33. = *Sclerocarya birrea* (A.Rich.) Hochst.

*Spondias borbonica* Baker, Fl. Mauritius 62. 1877. = *Poupartia borbonica* (Baker) J.F.Gmel.

*Spondias brunea* Urban, Symb. Antill. 7(2): 266. 1912. = *Bursera brunea* (Urban) Urban & Ekman.

*Spondias chakua* Buj. ex Baker, Fl. Mauritius 63. 1877. = *Poupartia castanea* Engl.

*Spondias chinensis* (Merr.) F.P.Metcalf, Journal of the Arnold Arboretum 12: 270. 1931. = *Allospondias lakonensis* (Pierre) Stapf.

*Spondias edmonstonei* Hook.f., Trans. Linn. Soc. London, Bot. 20: 230. 1847. = *Bursera graveolens* (H.B.K.) Triana & Planch. (Wiggins & Porter, 1971).

*Spondias elliptica* Rottb. ex Hook.f., Fl. Brit. India 2: 23. 1876. = *Buchanania lanzan* Spreng. (syn.: *Buchanania latifolia* Roxb.)

*Spondias falcata* Meisn., Flora 27: 349. 1844 (nom. rej.). = *Harpephyllum caffrum* Bernh. ex Krause.

*Spondias guianensis* (Aubl.) Klotzsch ex Engl. in A. DC. & C. DC., Monogr. phan. 4: 277. 1883 = *Tapirira guianensis* Aubl.

*Spondias haplophylla* Airy Shaw & Forman, Kew Bull. 21: 17. 1967. = *Haplospondias brandisiana* (Kurz) Kosterm.

*Spondias indica* (Wight & Arn.) Airy Shaw & Forman, Kew Bull. 21: 16. 1967. = *Solenocarpus indicus* Wight & Arn.

*Spondias klaineana* Engl., Bot. Jahrb. Syst. 36: 215. 1905. = *Antrocaryon klaineanum* (Engl.) Pierre.

*Spondias lakonensis* Pierre, Fl. forest. Cochinch. t. 375. 1898. = *Allospondias lakonensis* (Pierre) Stapf.

Spondias lakonensis Pierre var. hirsuta C.Y.Wu & T.L.Ming, Fl. Yunnan. 2: 374. 1979. = *Allospondias lakonensis* (Pierre) Stapf.

*Spondias laxiflora* (Kurz) Airy Shaw & Forman, Kew Bull. 21: 14. 1967. = cf. *Allospondias laxiflora* (Kurz) Lace.

*Spondias microcarpa* A. Rich. in Guill. & Perr., Fl. Seneg. tent. 151. 1830–1833. = *Pseudospondias microcarpa* (A. Rich.) Engl.

*Spondias oghigee* G. Don, Gen. hist. 2: 79. 1832. = *Lannea coromandelica* (Houtt.) Merr.

*Spondias parviflora* Willd. ex Schltdl., Linnaea 14: 295. 1840. = *Tapirira guianensis* Aubl.

*Spondias petelotii* (Tardieu) Kosterm., Reinwardtia 11(1): 55. 1992. (taxonomic syn. of *Dracontomelum* [sic] *petelotii* Tardieu) = cf. *Choerospondias*.

*Spondias philippinensis* (Elmer) Airy Shaw & Forman, Kew Bull. 21: 15. 1967. = *Solenocarpus philippinensis* (Elmer) Kosterm.

*Spondias pleiogyna* F.Muell., Fragm. 4(26): 78. 1864. = *Pleiogynium timoriense* (DC.) Leenh.

*Spondias pubescens* Baker, Fl. Mauritius 62. 1877 (non Bouton ex Steud.). = *Poupartia pubescens* (Baker) Engl.

*Spondias romblonensis* Elmer, Leafl. Philipp. Bot. x. 3683. 1939. = *Parishia malabog* Merr. *sensu* Ding Hou 1978.

*Spondias solandri* Benth., Flora austral. 1: 492. 1863. = *Pleiogynium timoriense* (DC.) Leenh.

*Spondias simplicifolia* Rottl., Ges. Naturf. Freunde Berlin Neue Schriften 4: 187. 1803. = *Buchanania angustifolia* Roxb.

*Spondias soyauxii* Engl., Bot. Jahrb. Syst. 36(2): 215. 1905. = ?*Antrocaryon soyauxii* (Engl.) Engl.

*Spondias tonkinensis* Kosterm., nom. illegit. based on same type as *Dracontomelon petelotii*.

*Spondias ?wirtgenii* [sic] Hassk. in Flora 25(2) Beibl. 46. 1842. = *Lannea coromandelica* (Houtt.) Merr.

### Dubious taxa

*Spondias aurantiaca* Schumach. & Thonn., Beskr. Guin. pl. 225. 1827. [no specimens cited]

*Spondias dubia* A.Rich. in Guill. & Perr., Fl. Seneg. tent. 1: 153. 1830. (taxonomic syn. of Spondias lutea L. var. dubia (A. Rich.) Marchand, Rev. Anacardiac.: 156. 1869) [no specimens cited; no illustration]

Spondias dulcis L. var. mucroserrata Engl. [type photo at NY of alleged Pavón coll. deposited in G no. 23181].

*Spondias nigra* Arruda ex Almeida, Diccionario de botanica brasileira: 177. 1873. [no type or specimens cited]

Spondias purpurea L. forma lutea Fawc. & Rendle, Fl. Jamaica 5: 17. 1926. [no specimens nor illustrations cited.]

*Spondias venosa* Colla ex Mart., nom. dub.; Herbarium Pedemontanum 2: 37. 1834. (no specimen found at TO). Probable orthographic variant/error based on *Spondias venulosa*.

*Spondias viridiflora* Fée, Cat. Method. Pl. Strasb.: 110. 1836. [no specimens cited]

*Spondias zanzee* G. Don, Gen. Hist. 2: 79. 1832. = ?*Pseudospondias microcarpa* (A.Rich.) Engl.

## Supplementary Material

XML Treatment for Spondias

XML Treatment for Spondias admirabilis

XML Treatment for Spondias dulcis

XML Treatment for Spondias expeditionaria

XML Treatment for Spondias globosa

XML Treatment for Spondias macrocarpa

XML Treatment for Spondias mombin

XML Treatment for Spondias purpurea

XML Treatment for Spondias radlkoferi

XML Treatment for Spondias testudinis

XML Treatment for Spondias tuberosa

XML Treatment for Spondias venulosa

## Figures and Tables

**Figure 21.**
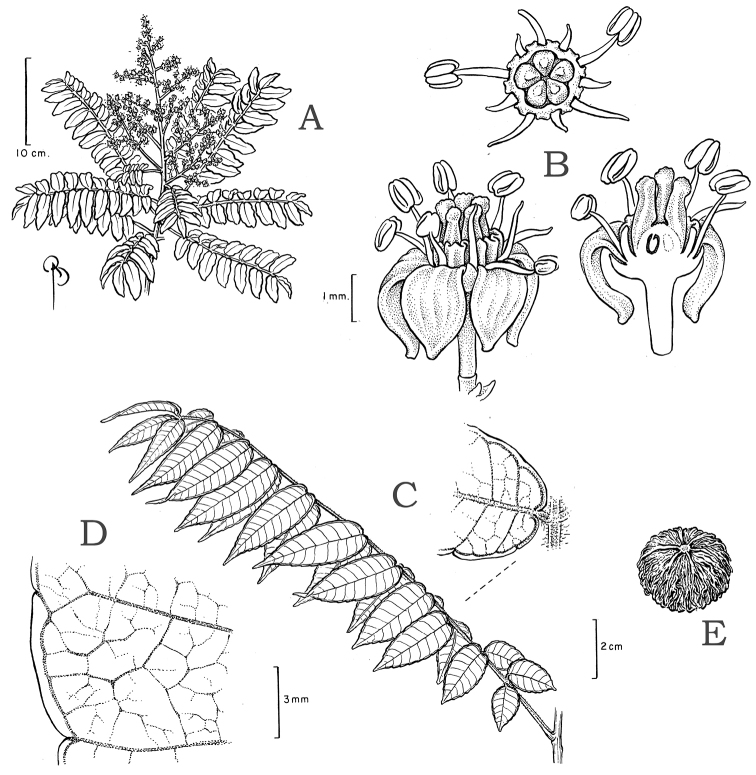
*Spondias expeditionaria*. **A** Flowering branchlet **B** Flower at anthesis and articulated pedicel (left), longisection through flower at center (right), and top view of stamens (most missing anthers), disk, and pistil **C** Whole leaf and detail of rachis and leaflet base (inset) **D** Detail of leaflet venation and margin **E** Endocarp. **A–E** from *Lorenzi s.n.* (NY).

## Supplementary materials

Supplementary material 1 Exsiccatae of *Spondias* in the Neotropics Data type: Index of specimens examined Explanation note: Index of collectors, collector numbers, and corresponding *Spondias* species. This dataset is made available under the Open Database License (http://opendatacommons.org/licenses/odbl/1.0/). The Open Database License (ODbL) is a license agreement intended to allow users to freely share, modify, and use this Dataset while maintaining this same freedom for others, provided that the original source and author(s) are credited.John D. Mitchell, Douglas C. Daly
