# Supplementary material for: A revision of Spondias L. (Anacardiaceae) in the Neotropics
Source: PhytoKeys. 2015 Aug 5;(55):1–92. doi: 10.3897/phytokeys.55.8489 (PMC4547026; doi:10.3897/phytokeys.55.8489)
Supplement: Supplementary material 1 — Exsiccatae of Spondias in the Neotropics [file phytokeys-055-001-s001.docx]

**Exsiccatae of *Spondias* in the Neotropics**

*Acevedo-Rodríguez, P. 3436 (mombin).*

*Acevedo‑Rodríguez, P. & Reilly, A. 1963 (mombin).*

*Acevedo-Rodríguez, P. et al. 6033, 6837 (mombin).*

*Agra, M.F. & Silva 1518 (tuberosa).*

*Aguilar, R. 37 (radlkoferi).*

*Alcorn, J. C05, 1519, 3037 (mombin).*

*Alexiades, M. & Pesha 262 (mombin).*

*Allemão, F. & Cysneiro 328 (R14676, 73732) (mombin); 329 [R 14675, 73733], s.n. [R 73587] (tuberosa).*

*Allen, P. 272 (mombin); 7207 (purpurea)*

*Allen, P. & van Severen 6890 (radlkoferi); 7301 (mombin)*

*Almeida, S.S. & Lima 246 (mombin).*

*Alvaro, P. & Alvarez 736 (purpurea)*

*Alves, M. et al. [ALCB no.] 47955 (tuberosa).*

*Amith, J. & Hall 241 (purpurea).*

*Amith, J. & Rojas 390 (purpurea).*

*Anderson, A.B. & Rosário 1437 (mombin).*

*Anderson, A.B. et al. 2016 (mombin).*

*Andrade, W.M. & Figueiredo 115 (tuberosa).*

*Andreata , R.H.P. et al. 357, 504, 915 (admirabilis).*

*Angeli, C. 703 (admirabilis); 704 (dulcis)*

*Araújo, D. 7828 (admirabilis).*

*Araújo, D. & Souza 8229 (admirabilis).*

*Arbo, M. et al. 5776, 7239 (cf. tuberosa); 7777 (venulosa).*

*Arévalo A. & Reyes 57, 64 (globosa); 58 (mombin).*

*Ariza-Julia, L. & Jiménez 5914 (mombin)*

*Arnold, L. 549 (dulcis).*

*Asplund, E. 15634 (mombin), 17575, 20522 (purpurea).*

*Atha, D. 1085 (radlkoferi).*

*Atha, D. and Romero 1372 (dulcis)*

*Atha, D. et al. 6690 (purpurea).*

*Aulestia M. & Bainca 3521 (mombin × globosa).*

*Aulestia M. & Gonti 1769, 1947 (globosa).*

*Aulestia, M. & Grefa 125 (mombin × globosa).*

*Aulestia, M. et al. 332 (mombin × globosa); 402 (globosa); 1069, 1246 (mombin × globosa); 3020 (globosa).*

*Ayala, F. & Criollo 3982 (dulcis).*

*Ayala, F. et al. 3392 (globosa).*

*Baker, C.F. 95, 192, 205 (purpurea).*

*Balée, W. (mombin).*

*Balée, W. & Ribeiro 1897 (mombin).*

*Balick, M.J. et al. 1824 (radlkoferi), 2467 (purpurea).*

*Barbosa, C. 699 (mombin).*

*Barbosa, S. 138 (mombin).*

*Barkley, F.A. & Hernández 40729 (mombin).*

*Barkley, F.A. & Nickle 40151 (mombin).*

*Barrier, S. 3712 (dulcis); 5817 (mombin)*

*Bautista, H.P. 727 (tuberosa).*

*Beck, H.T. & Souza 156 (mombin).*

*Beck, S. et al. 19614 (mombin).*

*Belém, R.P. & Magalhães 973 (mombin).*

*Bennett, B. & Gómez 3711 (globosa).*

*Berendsohn, W.G. [LAGU ref. no.] WB‑01127 (purpurea).*

*Berlin, B. 870 (dulcis).*

*Bernacci, L. 174 [SPF no. 70509] (tuberosa).*

*Betancur, J. & Uribe 7518 (mombin).*

*Betancur, J. & Berrio 1961 (mombin).*

*Black, G.A. 54-17704 (tuberosa).*

*Blanchet, J.S. 3078 (tuberosa).*

*Blasido, E. and Becerra G. 213 (dulcis)*

*Boldingh, I. 1873a, 5025 (mombin).*

*Boom, B.M. & Rivera 6793 (mombin).*

*Bordenave, B. 173 (mombin).*

*Bovini, M.G. et al. 1878 (admirabilis).*

*Brade, A.C. s.n. [R 73761] (mombin); s.n. [R no. 7376] (venulosa).*

*Brand, J. & Ascanio 480 (radlkoferi).*

*Brand, J. & Cogollo 176 (radlkoferi).*

*Breedlove, D. 9560 (purpurea).*

*Brenes, A.M. 6799 (mombin).*

*Breteler, F. 3271, 3277, 4021 (mombin).*

*Britton, N.L. 2270 (mombin).*

*Britton. N.L. & Britton 218 (purpurea).*

*Britton, N.L. & Broadway 2647 (mombin).*

*Britton, N.L. & Cowell 125 (mombin).*

*Britton, N.L. & Wilson 343 (purpurea).*

*Britton, N.L. et al. 457, 5817 (mombin); 9592 (purpurea).*

*Britton, E. 2911 (mombin).*

*Bro. León 2425 (mombin).*

*Broadway, W.A. 1780 (purpurea); 3614 (mombin); 5281 (purpurea).*

*Brochado, A.L. & Silva 204 (tuberosa).*

*Brokaw, N. 42, 206 (radlkoferi).*

*Brown. A. et al. 1656 (mombin); 1673 (purpurea).*

*Bullock, S.H. 1061 (purpurea).*

*Bunting, G. 5130, 5446, 7172, 7403 (mombin).*

*Bunting, G. et al. 11234,12453 (mombin).*

*Burchell, W.J. 1197 (venulosa).*

*Burger, W.A. & Liesner 6667(purpurea).*

*Burger, W.A. & Ramírez B. 4074 (purpurea).*

*Cabrera, E. & Cabrera, 3331, 5583, 6536 (radlkoferi).*

*Calderón, S. (radlkoferi).*

*Callejas, R. et al. 4419, (mombin); 4767, 4906 (radlkoferi), 4939 (mombin); 5743 (radlkoferi).*

*Calónico Soto, J. et al. 23370 (radlkoferi).*

*Calzada, J. 1813 (radlkoferi); 6119 (mombin).*

*Carauta, P. 552 (tuberosa).*

*Carballo, R. & Carrillo 325 (purpurea).*

*Cárdenas-L., D. et al. 1644, 2866 (mombin); 10775 (globosa).*

*Cardona N., F. et al. 1644 (mombin).*

*Cardoso, D. & Ferreira 2234 (tuberosa).*

*Cardoso, D. & Santos 1935 (venulosa).*

*Cardoso, D. et al. 716 (venulosa); 876 (tuberosa).*

*Carlson, M.C. 2000, 3312 (purpurea).*

*Carnevali, G. et al. 6514 (radlkoferi).*

*Carter, A. & Chisaki 1265 (purpurea).*

*Carvalho, A. et al. 3767 (tuberosa); 6493 (cf. tuberosa).*

*Castillo, J.J. & Castillo 1792 (purpurea); 1851, 2865 (mombin).*

*Castillo, A. 727 (mombin).*

*Cerón, C. 365 (mombin × globosa).*

*Cerón, C. & Hurtado 3912 (mombin × globosa).*

*Cerón, C. & Palacios 3016 (mombin × globosa).*

*Cervi, A. et al. 7358 (mombin).*

*Chacon, G. 1019 (mombin).*

*Chaplin, G.E. C556 (radlkoferi)*

*Chavarría, M.M. 590 (mombin); 958 (purpurea).*

*Chaviel, A. 52 (mombin)*

*Chota, J. 5 (globosa).*

*Cid Ferreira, C.A. & B. Nelson 3066 (globosa).*

*Cid Ferreira, C.A. et al. 10116A, 10879 (testudinis).*

*Clark, J. (dulcis).*

*Clark, J. et al. 1102, 1143 (mombin × globosa).*

*Clemente, [SP no.] 45460 (venulosa).*

*Coêlho, L. et al. 2017 (testudinis).*

*Combs, R. 24 (mombin).*

*Contreras 85, 6926 (radlkoferi); 8666 (purpurea); 8786, 16635, (radlkoferi).*

*Coradin, L. et al. 5945 (tuberosa).*

*Cornejo, X. & Bonifaz 5390 (mombin).*

*Correll, D. 50042 (purpurea).*

*Costa, S.A. 17, 176, 194 (tuberosa)*

*Costello, A. & Saraiva 25, 88 (testudinis).*

*Costello, A. 10, 11 (globosa).*

*Costello, A. et al. 50 (testudinis).*

*Crane, P.R. 458 (radlkoferi).*

*Croat, T.B. 8432 (mombin); 1824, 3116 (purpurea); 4929, 5736, 6453, 10082, 10218, 11682, 12580, 14004, 14830 (radlkoferi); 19180, 19375, 19628 (globosa); 19752 (dulcis); 20637 (globosa); 24728 (radlkoferi).*

*Croat, T.B. & Graham 59795 (radlkoferi).*

*Cuadros-V., H. 3098 (mombin).*

*Cuatrecasas, J. & Llano 24202 (mombin).*

*Cuatrecasas, J. & Willard 26106 (radlkoferi).*

*Cufodonti 248, Jiménez, Q. 1561 (purpurea.)*

*Cufodontis, G. 634 (mombin).*

*Curran, H.M. 43 (mombin); 322 (dulcis).*

*Curran, H.M. & Haman 1003 (mombin).*

*Daly, D.C. 137, 13105 (purpurea)*

*Daly, D.C. et al. 5620 (mombin × globosa); 7174 (mombin); 7251 (testudinis); 7836 (globosa), 7902 (mombin), 8029, 8056 (globosa); 8182 (testudinis); 8361 (dulcis); 8472 (globosa), 8549 (mombin); 9125, (mombin), 9314, 9379, 9548, 10039 (globosa); 10175, 10206 (mombin); 11002 (globosa), 12052 (testudinis); 12209 (mombin); 13968 (purpurea).*

*D’Arcy, W. 2110 (purpurea).*

*D'Arcy, W. et al. 13354 (mombin.)*

*Davidse, G. & González 14459, 14872 (mombin).*

*Davis, H. et al. 34 (dulcis)*

*de Bruijn, J. 1491 (mombin).*

*de la Cruz, J.S. 1951, 3839 (mombin).*

*de Nevers, G. & Herrera 5634 (mombin).*

*Degener, O. 3709 (mombin).*

*Degener, O. & Degener 26754 (radlkoferi).*

*Demuner, V. 1583 (expeditionaria).*

*Demuner, V. & Bausen 566 (venulosa).*

*Demuner. V. et al. 1945, 2382, 3783 (venulosa.)*

*DeWalt, S.J. 316 (mombin).*

*Díaz, C. & Ramírez 9551 (globosa).*

*Diaz, C. et al. 3172 (mombin).*

*Dik, A. 830 (globosa).*

*Dik, A. & Andi 906, 975 (mombin x globosa)*

*Dik, A. & Enomenga 1150 (mombin × globosa).*

*Dodson, C. et al. 8837 (radlkoferi); 14967 (globosa).*

*Donnell-Smith, J. 1464 (purpurea); 2087, 6472 (radlkoferi).*

*Dorantes, F. 45 (mombin); 2779 (radlkoferi).*

*Drouet, F. 2677(dulcis).*

*Ducke, A. 799 (tuberosa); [MG no.] 1651 (mombin).*

*Duke, J.A. 11423 (mombin); s.n. [1963-1964] (purpurea).*

*Duque‑Jaramillo, L.F. 4101 (globosa).*

*Durán, R. et al. 2616 (mombin).*

*Duss, Père 322 (mombin); 323 (dulcis); 326 (purpurea); 3272 (mombin); 3278 (purpurea); 3760 (dulcis)*

*Dwyer, J.D. 1458, 1699 (radlkoferi); 14638 (purpurea)*

*Edwards, J.G. 370 (purpurea)*

*Eggers, H.F.A. von 389 (mombin).*

*Eiten, G. 388 (mombin).*

*Eiten G. & Eiten 10523,10810 (tuberosa).*

*Ekman, E.L. 5854 (mombin); 12532 (x robe [mombin x purpurea]); 14615 (dulcis).*

*Espina, J. et al. 2707 (mombin).*

*Espinal T. & Pérez F. 700 (radlkoferi).*

*Estrada, U. et al. 8 (mombin); 9 (radlkoferi)*

*Evans et al. 1955, 2663, 2759 (mombin).*

*Fanshawe, D. F930 (mombin).*

*Farias, G. 36, 37, 38, 40 (venulosa); 475 (macrocarpa).*

*Farney de Sá, C. & Gomes 4046, 4172 (admirabilis); 4166 (venulosa)*

*Farney de Sá, C. et al. 2650 (mombin); 3323 (venulosa); 3957 (admirabilis), 4083 (venulosa).*

*Farney de Sá, C. & Mota 1972 (mombin).*

*Fendler 1308 (purpurea); 1310 (mombin).*

*Fendler, A. 238 (mombin).*

*Fernandes, A. s.n. [EAC no. 6044] (tuberosa); 3989 (mombin).*

*Fernández, A. 229, 303, 6814 (mombin).*

*Fernández, A. & L. Mora 1334 (mombin).*

*Fernández-Casas, J. & Susanna 8566, 8567 (globosa).*

*Fishlock, W. 443 (mombin).*

*Fleury, M. 729 (purpurea).*

*Flores M., A. 151, 1218 (purpurea).*

*Flynn, T. & Lorence 5807 (purpurea).*

*Folli, D. A. 1534 (expeditionaria); 1608 (venulosa); 1614 (macrocarpa); 1757 (tuberosa).*

*Fonnegra G., R. & Corral 1633 (purpurea)*

*Fonnegra G., R. et al. 1718 (mombin); 1872, 7850 (radlkoferi).*

*Fontana, V.L. et al. 5616 (mombin).*

*Forero‑González, E. 582 (dulcis).*

*Foster, R. 2317 (mombin).*

*Foster, R. & Terceros 13386 (mombin).*

*França, F. et al. 5464 (tuberosa).*

*Franklin s.n. (Escola Polytécnica 5368), [R no. 73729][LISU 5368] (admirabilis).*

*Freire, E. and Suárez 5007 (globosa)*

*Freitas, L. s.n. [EAC no. 16821] (mombin).*

*Fróes, R.L. 1990 (mombin); 12671/37, 20079 (macrocarpa).*

*Fuchs, H. & Zanella 22052 (mombin).*

*Galdames, C. & Guerra 1928 (radlkoferi).*

*García, A. R. & Martínez 52 (purpurea).*

*Garwood, N.C. et al. 598 (purpurea).*

*Garzón, T.A. & Lopera 103 (radlkoferi).*

*Gaudichaud-Beaupré, C. 826 (venulosa).*

*Gaumer, G.F. 432 (purpurea).*

*Gaumer, G.F. & sons 23885 (radlkoferi).*

*Gehrt [Hatschbach] [SP no.] 39886 (venulosa).*

*Gentle, P. 2739 (mombin); 5017 (radlkoferi).*

*Gentry, A.H. 842 (radlkoferi); 1435 (purpurea); 6310 (radlkoferi).*

*Gentry A.H. & Ayala 15546 (mombin × globosa).*

*Gentry, A.H. & Brand 36710 (radlkoferi).*

*Gentry, A.H. & Rentería 24247 (purpurea).*

*Gentry, A.H. & Zardini 49965 (tuberosa).*

*Gentry, A.H. et al. 26780 (mombin); 29889, 38005, 56212(globosa); 60562a (mombin); 60884, 68896, 72150 (globosa)*

*Gentry, H.S. 7108, 7317 (purpurea)*

*Germán Ramírez, M.T. et al. 239 (purpurea).*

*Gillis, W.T. 5876 (mombin); 9268 (dulcis); 11119 (mombin);*

*Gillis, W.T. & Plowman 10265 (mombin).*

*Gilly, C.L. et al. 86 (purpurea).*

*Ginzberger, A. 1489 (tuberosa).*

*Giraldo‑Cañas, D. 919 (radlkoferi).*

*Glaziou, A.F.M. 827 (venulosa), 2086 (tuberosa); 2912 [R no. 8460] (dulcis); 13678 [R no. 8461], 13679 (macrocarpa); 17584 [R no. 8459] (admirabilis).*

*Glenboski, L.L. C-201 (mombin × globosa).*

*Gómez. J.P.N. et al. 561 (mombin).*

*González, A. & Ortega, F. 1336 (mombin).*

*González, J.C. & Serrano JCG00544 (purpurea).*

*González, O. 82 (mombin).*

*Gooding, E. 80 (mombin).*

*Gottsberger, I. & Gottsberger 11-191182 (mombin); 14-28173 (tuberosa).*

*Graham, J. & Schunke Vigo 555 (dulcis).*

*Grayum, M.H. 11809 (x robe [= mombin x purpurea])*

*Grández, C. & Jaramillo 2042 (mombin × globosa).*

*Gregory, D.P. 592 (purpurea).*

*Griffis, J.D. & Brokx s.n. (mombin).*

*Grijalva, A. 782 (mombin × globosa); 2415 (mombin).*

*Gudiño, E. et al. 2270 (cf. globosa).*

*Guedes, M. L. 5399 (tuberosa).*

*Guillemin, J.B.A. 243 (venulosa).*

*Guillén Villaroel, R. & Roca 3034 (mombin).*

*Gutiérrez R., A. 129 (testudinis).; 162 (globosa); B-05-2-J (mombin)*

*Hage, J.L. 230, 1643, 2186 (venulosa).*

*Hagen, C. & W. von Hagen 1096, (radlkoferi); 1160 (purpurea); 1259 (radlkoferi).*

*Hahn, W. 1432 (mombin).*

*Hall, D. & Bockus 7873 (purpurea).*

*Hampshire, R.J. et al. 1123 (purpurea).*

*Harley, R. & Taylor 27064 (tuberosa).*

*Harley, R. et al. 27127, 55198 (tuberosa).*

*Harris et al. 1174 (mombin) 9230 (mombin)*

*Hatschbach, G. & Silva 60062 (macrocarpa).*

*Hatschbach, G. et al. 55163 (tuberosa); 60365, 65551 (mombin); 65979 (tuberosa); 76338 (mombin); 77113 (purpurea).*

*Haught, O. 1572 (radlkoferi); 2187 (mombin).*

*Hawthorne, W.D. & Hughes 350 (mombin).*

*Hawthorne, W.D. et al. 459 (dulcis); 480, 480B (mombin); 519, 523 (purpurea).*

*Heithaus, E. 119 (radlkoferi).*

*Heller, A. 1225 (mombin).*

*Heringer, E.P. 60 (RB no. 83460) (macrocarpa).*

*Hernández, H. 2694 (radlkoferi); 2695 (mombin).*

*Herrera, H. & Arosemena 1834 (radlkoferi).*

*Hinton, G.B. 3217, 3316, (purpurea); 6599 (mombin); 10037 (purpurea).*

*Hinton, G.B. et al. 5676 (purpurea).*

*Hodge, W.H. 6716 (purpurea).*

*Hoehne, F.C. & Gehrt [Hatschbach] [SP no.] 36835 (venulosa).*

*Hohenkerk, L.S. 869 (mombin).*

*Hollowell, T. 309 (mombin).*

*Holm, R.W. & Iltis 952 (mombin).*

*Hopkins, M. et al. 1454 (mombin).*

*House, P. 1919 (radlkoferi).*

*Howard., R.A. 4911, 11184 (mombin).*

*Howard, R.A. & Howard 9237 (purpurea), 10049 (purpurea); 19455 (mombin)*

*Howard, R.A. & Proctor 13531 (dulcis).*

*Huamán, M. and Francis 84 (testudinis)*

*Huashikat, V. 392 (globosa).*

*Huber, O. & Canales 6342 (mombin).*

*Huber, J. 2099 (dulcis).*

*Hughes, C. 474 (mombin).*

*Hurtado, F. 3030, 3070 (mombin × globosa).*

*Ibarra M., G. & Sinaca 2332 (radlkoferi).*

*Inspectoria Florestal 35 (R no. 27641) (admirabilis).*

*Irvine. D. 653 (dulcis).*

*Irwin, H. et al. 48317 (mombin).*

*Jack, J.G. 5039 (mombin).*

*Jacobs, B. 3293 (radlkoferi).*

*Jacquemont, V. 244 (purpurea).*

*Janovec, J. et al. 2572 (mombin).*

*Jaramillo, J. & Buitrón 15733 (mombin).*

*Jaramillo, J. et al. 13845 (mombin).*

*Jardim, A. 717 (testudinis.)*

*Jardim, A. et al. 1971 (venulosa); 1972 (mombin).*

*Jiménez-Madrigal, Q. & Ramírez 1888 (radlkoferi).*

*Jiménez-Madrigal, A. 590 (mombin).*

*Jiménez S., H. 1325 (mombin).*

*Johnston, I.M. 787, 964 (purpurea).*

*Jones, G. & L. Facey 3240 (mombin).*

*Kayap, R. 191 (mombin × globosa); 776 (globosa).*

*Killeen, T. 3584, 3815 (mombin × globosa); 3815 (mombin).*

*Killeen, T. & Krudenky 3612 (mombin).*

*Killeen, T. et al. 3403 (mombin).*

*Killip, E.P. & Smith 27661 (purpurea); 29266 (globosa).*

*Klug, O. 2610 (purpurea).*

*Kluge, H.C. 39 (radlkoferi); 43 (mombin).*

*Korning, J. & Thomsen (SEF No.) 8836 (cf. globosa).*

*Kröll-Saldaña, B. 694 (globosa).*

*Krömer, T. & Acebey 526 (mombin).*

*Krukoff, B.A. 1554, 5767, 6056, 6434, 8329 (mombin).*

*Kruse, H. 1100 (purpurea).*

*Kubitzki, K. & Poppendieck 79‑267 (tuberosa).*

*Kuhlmann, J.G. (GUA no.) 11051 (venulosa); [RB no. 40595] (tuberosa).*

*Langsdorff, G.H. s.n. (venulosa).*

*Lanjouw, J. & Lindeman 1388, 3152 (mombin).*

*Lanna Sobrinho, J.P. 1587 (GUA 538)(admirabilis).*

*Lao M., R. 83, 112 (testudinis).*

*Laughlin, R.M. 1332 (mombin).*

*Lavastre, Bro. B. A. 2117 (mombin).*

*Leavenworth, W.C. & Hoogstraal 1540 (purpurea).*

*Leitman, P.M. et al. 284 [RB 27217] (venulosa).*

*Lent, R. 1148 (radlkoferi).*

*Lentz, D. 1716 (purpurea).*

*Leonard, E. 4079 (mombin).*

*Leonard, E. & Leonard, 15722 (mombin).*

*Lewis, G.P. & Pearson 1154 (tuberosa).*

*Lewis, G.P. 2520 (purpurea).*

*Liesner, R. & González 5624 (mombin).*

*Lima, H.C. 2883 [RB 288338] (venulosa.)*

*Lima, H.C. et al. 3988 (venulosa).*

*Lima, L. et al. 502 (testudinis).*

*Liogier, A. 19082 (mombin); 3672 (dulcis)*

*Liogier, A. & Liogier 28702 (mombin).*

*Lisboa, P. & Silva 4521 (tuberosa).*

*Lisboa, P. et al. 3018 (mombin).*

*Little, E. 6289 (mombin); 6366, 6487 (purpurea); 8092, 13440 (mombin); 13526 (purpurea); 14914 (dulcis); 16442, 23781,(purpurea); 25273 (radlkoferi).*

*Little, E.L. & Little 9760 (mombin).*

*Little, E. et al. 23839 (mombin).*

*Littmann, E. & Pennington 9036 (mombin).*

*Lizot, J. 1972‑17 (mombin).*

*Lobo, M. 89 (mombin).*

*Lombardi, J. 2080 (tuberosa).*

*Lorence, D.H. 7967(dulcis).*

*Lorenzi, H. 1-548 (mombin); 6074 (cf. tuberosa).*

*Lundell, C.L. 894 (radlkoferi); 3283 (purpurea); 4356 (mombin); 5017, 15969 (radlkoferi).*

*Lundell, C.L. & Contreras 20240, 20700 (radlkoferi).*

*Lutz, A. 864 [R 120202] (venulosa).*

*Luz, A.A. 36, 245, 248 (venulosa).*

*Maceda, A.P. 569 (globosa); 999 (mombin).*

*Macedo 5451 (venulosa).*

*Machado, I.C. s.n. [EAC no. 23729] (mombin).*

*Machado, R.B. et al. 301 (mombin).*

*Macía, M.J. et al. 6638 (mombin).*

*Maciel, U. & Cordeiro 236 (mombin).*

*Maciel, U. & Santos 1922 (mombin).*

*Madrid N., E. et al. 1264 (radlkoferi).*

*Madsen, J. 63428 (purpurea).*

*Magallanes, J. 3887 (purpurea).*

*Magnago, L.F.S. et al. 1061 (venulosa).*

*Mamani M., F. & Saucedo 638 (mombin).*

*Marcano-Berti, L. 157, 395 (mombin).*

*Marin, E. 1328 (mombin).*

*Marshall, N. et al. 356 (purpurea).*

*Martin, G. & Plowman 1781 (dulcis.)*

*Martin, R.T. & Lau-Cam 1252 (globosa).*

*Martinelli, G. 5552 (RB 206648) (admirabilis).*

*Martínez C., G. 1402 (mombin).*

*Martínez S., E. 32251 (mombin).*

*Martínez S., E. et al. 23230 (purpurea); 31492 (radlkoferi).*

*Martínez, S. s.n. [LAGU no. ISF0063], [LAGU no. ISF00102] (mombin).*

*Marulanda, O. & Márquez, S. 1871 (mombin).*

*Mattos Silva, L.A. 1640 (tuberosa); 2299, 2301 (cf. tuberosa)*

*Matuda, E. 2617 (mombin); 18654 (radlkoferi).*

*McDaniel, S. & Rimachi Y. 17543 (globosa).*

*Meigs, R.A. 1197(purpurea).*

*Mejía, M.M. & Zanoni 6781 (purpurea); 7824 (mombin).*

*Mejía, M.M. et al. 13131 (purpurea).*

*Mello-Silva, R. et al. 1412 (tuberosa).*

*Mendoza, P. & Amith 1430 (mombin); 1447 (purpurea).*

*Meneces, Brig. 690 (testudinis).*

*Metcalf, R.D. & Cuatrecasas 30055 (mombin).*

*Miller, A.J. 95 (purpurea).*

*Miller, A.J. & Avila-Días 306 (purpurea).*

*Miller, A.J. & Merello 228 (mombin); 230 (dulcis).*

*Miller, J.S. & Yépez 703 (globosa).*

*Miller, A.J. & Kay 99 (purpurea); 101 (dulcis); 102 (purpurea); 104 (mombin); 105 (purpurea); 106 (mombin); 107 (dulcis); 108 (mombin).*

*Miller, A. et al. 123, 126 (radlkoferi), 149 (mombin); 158,178, 241, 245 (purpurea); 253 (mombin); 255 (purpurea); 256 (mombin); 258 (purpurea); 259 (mombin); 261, 262 (purpurea); 262 (mombin); 263, 275, 279, 284, 286, 288, 290, 294, 314 (purpurea); 318, 321 (mombin); 322, 323, 325, 326, 327, 329, 330, 331, 332, 333, 334, 335 (purpurea).*

*Miranda-Moyano, N. & Moya 446 (globosa).*

*Mitchell, J.D. 121 (mombin); 120 (radlkoferi).*

*Molina, A. & Molina 35164 (purpurea).*

*Monro, A.K. et al. 3624 (purpurea); 3722 (mombin).*

*Monteiro, O.P. & Damião 608 (INPA 56.458) (testudinis).*

*Moraes, M. 1046 (mombin).*

*Moran, R. et al. 6292 (mombin); 6293 (cf. mombin x radlkoferi); 6295 (radlkoferi).*

*Moreno, P. 365 (mombin); 1580, 1931 (purpurea); 2130 (mombin); 2827 (purpurea); 26293 (radlkoferi).*

*Mori, S.A. & Boom 15324 (mombin).*

*Mori, S.A. & Mitchell 18777 (purpurea).*

*Mori, S.A. et al. 11208 (tuberosa); 16508, 21534 (mombin).*

*Mori, S.A. & Kallunki 5776 (mombin).*

*Mostacedo, B. 237 (purpurea).*

*Moya, G. & Reyes 146 (globosa).*

*Müller, F. 1588 (purpurea).*

*Murphy, H. 299 (globosa).*

*Museu Nacional [R no.] 73764 (tuberosa)*

*Nash, G. 392 (purpurea); 934 (mombin)*

*Nee, M. 6729 (radlkoferi); 39607 (globosa); 39811 (mombin); 44157 (dulcis).*

*Nee, M. & Coimbra 36990 (purpurea).*

*Nee, M. & Diggs 24633 (mombin).*

*Nee, M. & Vargas 45018 (mombin).*

*Nee, M. et al. 27751 (purpurea); 50353 (mombin).*

*Neill, D.A. 1695, 1778 (mombin); 7160 (mombin × globosa).*

*Neill D.A. & Palacios 7079 (globosa).*

*Neill , D.A. et al. 6250, 8288 (globosa); 11619 (mombin).*

*Nelson, B.W. 749 (mombin); 786 (globosa).*

*Nevling, L.I. 289 (dulcis).*

*Nevling, L.I. & Gómez-Pompa 485 (purpurea).*

*Nuñez, P. 6541 (purpurea).*

*Nuñez, P. & Tapuy 586 (globosa); 5620 (mombin × globosa).*

*Oliveira, C. 3 (mombin).*

*Omawale & Persaud 94 (dulcis); 118 (purpurea).*

*Ortega 327 (purpurea).*

*Oscar, L.E. et al. 1000 (mombin).*

*Ososki, A. & Saborío 468 (mombin).*

*Palacio, L. 25 (purpurea).*

*Palacios, W. 2280 (globosa).*

*Palacios, W. & Neill 1278 (globosa).*

*Palacios, W. et al. 355 (mombin × globosa).*

*Palmer, E. 402 (mombin); 998 (purpurea).*

*Pearson 10 (tuberosa).*

*Peckolt, T. 224 (BR 571985), s.n. (BR 571916), s.n. (BR 571982) (macrocarpa).*

*Pedra do Cavalo, G^o^. et al. 942 (tuberosa).*

*Peek 913 (mombin).*

*Peña-Chocarro, M.C. et al. 49 (mombin).*

*Pendry, C. et al. 822 (purpurea).*

*Pennell, F.W. 4683 (mombin).*

*Pennington, T.D. & Daza 16635, 16740, 16874 (dulcis).*

*Pennington, T.D. & Sarukhán 9105 (radlkoferi); 9237, 9269 (mombin); 9569, 9579 (radlkoferi).*

*Pennington, T.D. & Tenorio 10677 (purpurea), 10789 (globosa).*

*Pennington, T.D. et al. 13739 (mombin); 17244 (globosa).*

*Pereira, B. 2046 (mombin).*

*Pereira, B. & Alvarenga, D. 2895 (mombin).*

*Pereira, B. et al. 1698 (mombin).*

*Pereira, E. 9727 & Pabst 8616 (tuberosa).*

*Pereira-Silva, G. et al. 174 (globosa); 2391 (mombin); 3638 (venulosa); 5575, 8863 (mombin).*

*Pérez, B. 1426 (mombin).*

*Pérez-Zabala, J.A. et al. 2355 (mombin).*

*Peters, C.M. 6, 19 (globosa); 73 (dulcis); 191, 192 (mombin × globosa).*

*Peters, C.M. & Hammond 164 (globosa).*

*Phillips, O. & Chávez 636 (globosa).*

*Pickel, D. B. 590 (tuberosa); 2938 (mombin).*

*Piery, s.n. [R 73773][LISU 54197] (admirabilis).*

*Pinheiro, R. 33 (mombin).*

*Pinto, G. 623 (tuberosa).*

*Pipoly, J.J. 11742 (mombin).*

*Pirani, J. & Zappi 1019 (SPF no. 38167) (admirabilis).*

*Pirani, J. et al. (SPF no.) 21292 (tuberosa).*

*Pires, J. M. 13108 (mombin).*

*Pires, J. M. & Oliveira 896 (mombin).*

*Pires, J.M. & Santos 16245 (mombin).*

*Pires, J.M. & Westra 48842 (mombin).*

*Pittier, H. 3486 (purpurea); 11687 (mombin).*

*Plowman, T. et al. 7257 (globosa); 8702 (mombin).*

*Ponce C., F. & Cedillo T., R. (MEXU no.) 21530 (mombin).*

*Prance, G.T. & Schaller 26696 (mombin).*

*Prance, G.T. & Silva, M. 2423 (mombin).*

*Prance, G.T. & Steward 20105 (purpurea).*

*Prance, G.T. et al. 7873, (globosa); 8150, 10287, 10979 (mombin); 23806, 24555 (globosa).*

*Prévost, F. 3238 (dulcis).*

*Prior, A. s.n. (1850) (mombin)*

*Proctor, G. 29793 (radlkoferi).*

*Proença, C. 557 [SPF no. 46962] (mombin).*

*Purpus, C.A. 8154, 8157 (radlkoferi).*

*Pursell, R. et al. 8514 (mombin.)*

*Queiroz, L.P. 2604 (venulosa); 3451 (mombin)*

*Quesada, F. 295 (radlkoferi).*

*Quevedo, R.C. & Centurión 549 (mombin).*

*Quevedo, R.C. et al. 2548 (purpurea).*

*Rabelo, B.V. 1815 (mombin).*

*Ramamoorthy, T.P. 2370 (mombin).*

*Rampin, V.T. and Arantes 884 (dulcis)*

*Ratter, G. et al. 2702 (tuberosa); 4398, 6280 (mombin).*

*Ratter, G. R4669 (purpurea).*

*Redden, K. et al. 4879 (mombin).*

*Reinders, M. & Kissoon, J. 81(mombin).*

*Renson, C. 253, 272 (purpurea).*

*Rentería, E. 1628 (purpurea); 3242 (mombin).*

*Rentería, E. et al. 1915 (mombin); 4686 (radlkoferi).*

*Reyes-García, A. 2449 (radlkoferi).*

*Reyes-Gárcia, A. & Hampshire 1930 (purpurea).*

*Rezende, M.A. [RB] 482757 (venulosa).*

*Ribeiro, B. 4A (mombin).*

*Ribeiro, R.D. & Dantas 350 (admirabilis).*

*Ricardo, C.C. 600 (admirabilis).*

*Ricksecker, A. 387 (mombin).*

*Riera, B. 938 (purpurea).*

*Rimachi Y., M. 1815, 2543, 4605 (globosa).*

*Robleto, W. 760 (mombin).*

*Rodríguez, D. et al. 1539 (purpurea)*

*Romaniuc Neto, S. et al. 536 (mombin).*

*Romoleroux, K. 3239 (mombin × globosa).*

*Romoleroux, K. & Grefa 3239 (mombin).*

*Rosas, M. & Cardoso 2602 (mombin).*

*Rosa, N.A. & Cordeiro 804 (testudinis); 1719 (mombin).*

*Rosales, J.M. 394, 1055 (radlkoferi).*

*Rose, J.N. & Russell 19665 (mombin); 19757 (tuberosa).*

*Rose, J.N. et al. 3437, 13792 (purpurea).*

*Rueda, R.M. et al. 13903 (mombin).*

*Ruíz M., J.C. 1194 (mombin × globosa).*

*SEF 8836 (mombin x globosa)*

*Sabatier, D. & Molino 5030 (mombin).*

*Sabino, B. 90‑3‑41 (mombin).*

*Sagot, P.A. 196 (dulcis)*

*Sampaio, A.J. 5420 [R no. 18687] (mombin); 6255 [R no. 28735], 7919 [R 73580] (venulosa); 8219 [R no. 57992] (tuberosa).*

*Sandino, J. 2629 (mombin); 3599 (dulcis).*

*Sanoja, E. 1226 (mombin).*

*Santin, D.A. et al. s.n. [RB300478],[ RB300479], [RB 318764] (venulosa).*

*Saunders, J.G. 613 (mombin).*

*Schipp, W.A. 155 (mombin).*

*Schreiner, E.J. s.n. [R no. 73772] (tuberosa).*

*Schultes, R.E. & Black 8490 (globosa).*

*Schultes, R.E. & Reko 721 (radlkoferi).*

*Schultes, R.E. et al. 24119 (globosa).*

*Schunke, J.M. 250 (globosa).*

*Schunke V., J. 1223 (mombin).*

*Schunke Vigo, J. 14495 (purpurea).*

*Scolnik, R. 1193 (dulcis); 1194 (purpurea).*

*Seaforth, C.E. & Sylvester S2-28045 (mombin).*

*Seidel, R. et al. 5630, 5631 (mombin).*

*Seigler, D.S. DS-12288 (mombin).*

*Sermeño, A. 163 (purpurea).*

*Serna, O.L. 663 (mombin).*

*Sevilha, A.C. & Xavier 1863 (mombin).*

*Shafer, J.A. 996 (mombin); 1035a (purpurea); 1311 (mombin); 1525 (x robe [mombin x purpurea]).*

*Shepherd, J.D. 612 (radlkoferi).*

*Sierra, L. 8 (radlkoferi).*

*Silva, H.G.V. & L. Lima 51 (mombin).*

*Silva, M.A. et al. 3377 (mombin).*

*Silveira, M. et al. 475, 581 (testudinis); 1622 (globosa).*

*Silverstone-Sopkin, P. et al. 6225 (mombin).*

*Small, J.K. 2283 (purpurea).*

*Smith, A. 1777 (radlkoferi).*

*Smith, A.C. 3103 (mombin).*

*Smith, D.N. et al. 6406 (testudinis); 14061 (mombin); 14171 (globosa); 14322 (cf. mombin x testudinis)*

*Smith, H.H. 912, 1738 (mombin), 1746 (purpurea).*

*Smith, H. H. & Smith G160 (mixed collection: inflorescence of dulcis, leaves of S. mombin/*

*Soejarto, D.D. & Rentería 3566, 3572 (radlkoferi).*

*Soejarto, D.D. et al. 3993 (radlkoferi).*

*Solano Camacho, E. 436 (mombin).*

*Solomon, J. 14593 (globosa).*

*Sothers, C. & Santos 80 (globosa).*

*Souto, J. 43 (RB no. 151412) (tuberosa).*

*Souza Santos, F. 820 (macrocarpa).*

*Souza, V. 11 (venulosa); 244 (mombin); 267 (macrocarpa); 329, 340 (tuberosa)*

*Souza, V. et al. 390 [CVRD 4072] (expeditionaria).*

*Spada, J. 013/77 (macrocarpa); 1/77 (venulosa); 51 (venulosa).*

*Spichiger, R. & Encarnación 1095 (mombin x globosa).*

*Spruce, R.E. 4093 (purpurea).*

*St.-Hilaire, A.F.C.P. 186, 1026 (venulosa).*

*Stancik, J.F. et al. 126 (mombin)*

*Standley, P.C. 54022 (radlkoferi); 54289 (purpurea).*

*Stefano, M.V. et al. 200 (macrocarpa); 201 (venulosa); 211 (mombin); 225 (macrocarpa); 231 (venulosa); 250, 259, 260 (admirabilis).*

*Stehmann, J.R. (BHCB no.) 20855 (venulosa).*

*Stergios, B. & Taphorn 4829 (mombin).*

*Stevens, W.D. 20769 (mombin).*

*Stevens, W.D. & Grijalva 14724 (purpurea).*

*Stevenson, P. 60 (mombin); 109 (globosa); 178 (mombin).*

*Steyermark, J.A. 45214 (radlkoferi); 86618 (mombin); 99828 (globosa).*

*Steyermark, J.A. & Fernández 99734 (radlkoferi).*

*Steyermark, J.A. & Manara 110455 (mombin).*

*Steyermark, J.A. et al. 102015 (globosa); 108665 (mombin).*

*Stijfhoorn, E. 867 (mombin).*

*Struwe, L. & Specht 1097 (dulcis).*

*Sucre, D. 8770 (admirabilis).*

*Taylor, E. et al. E1045 (mombin).*

*Tello, J.C. 258 (mombin); 354, 396 (testudinis); 451 (mombin).*

*Tessmann, G. 3233, 3941, 4958 (globosa).*

*Thomas, W.W. et al. 4623 (mombin); 6823 (macrocarpa); 9567 (purpurea); 12363 (venulosa).*

*Thomas, W.R. 7774 (dulcis).*

*Thorne, R. & Proctor 48066 (purpurea).*

*Tina, F. & Tello 2012 (globosa).*

*Tiwari, S and Mengharini 409 (mombin); 624 (dulcis)*

*Toledo, F.R.N. et al. [BHCB 19288] (venulosa).*

*Toledo. F.R.N. et al. 556 (mombin).*

*Tonduz, A. 6997, 9924, 13920 (mombin); 13925 (radlkoferi)*

*Torres M., J. 88, 350 (globosa).*

*Trinta, Z. & Fromm 1050 (venulosa).*

*Trujillo, B. & Pulido 15469 (mombin).*

*Tuberquía, D. et al. 1415 (radlkoferi).*

*Tún-Ortiz, R. 115, 1066, (radlkoferi); 1902 (mombin).*

*Türckheim, H. von 1778 (purpurea); 4099 (radlkoferi).*

*Ule, E. 7255 (tuberosa); 7959 (mombin).*

*Urrego G., L. 538 (globosa).*

*Valenzuela, L. et al. 2457 (purpurea).*

*Vallecillo, L. 48 (purpurea).*

*van der Werff, H. & González 4820 (mombin).*

*van Proosdij et al. 581 (mombin).*

*Vargas, I. et al. 423, 1365, 1831, 1858, 3837, 5262 (mombin).*

*Vásquez, B. 565 (mombin).*

*Vásquez, R. & Jaramillo, N. 778, 2402, 9352, 9445, 9868 (globosa); 10481 (testudinis); 12866 (globosa); 13177 (mombin × globosa); 17379 (globosa).*

*Vásquez, R. et al. 4873 (mombin × globosa); 7031 (mombin); 11310, 12159 (globosa); 25831 (mombin); 34534, 34637 (globosa).*

*Vásquez-Torres, M. 395 (radlkoferi).*

*Vázquez-Torres, M. et al. 638 (mombin); 3366 (radlkoferi).*

*Ventura, E. & López 1800, 3787 (radlkoferi).*

*Ventura, A. 4449 (mombin).*

*Villacorta M., R. & Giammattei 2548 (purpurea).*

*Vincelli, P. 740 (purpurea).*

*Walker R. 1410 (radlkoferi).*

*Wallace, R.B. & Painter 114 (mombin).*

*Wallnöfer, B. et al. 5814 (radlkoferi).*

*Warush, J. [RBAE] 69 (globosa).*

*Watson, S. 53 (radlkoferi).*

*Webster, G. 16786 (radlkoferi).*

*Weissich, P. 213 (purpurea).*

*Wendt, T. et al. 3116, 4013 (radlkoferi).*

*White, S. 416 (mombin).*

*Whitefoord, C. & Eddy 434 (purpurea).*

*Wiemann, D. 38 (radlkoferi); 120 (mombin).*

*Wiggins, I.L. 18510 (purpurea).*

*Wilbert, W. et al. 262 (mombin).*

*Wiley, J.R. 547 (radlkoferi)*

*Williams, L. 1761, 3401 (globosa); 4933 (mombin).*

*Williams, L.O. 12885 (mombin).*

*Williams, L.O. & A. Molina 13440 (radlkoferi).*

*Williams, R.S. 380 (purpurea).*

*V. Wittingthon 44 (dulcis).*

*Woodson, R. et al. 1206 (mombin).*

*Wurdack,J.J. 315 (dulcis); 2152 (mombin).*

*Wurdack, J.J. & Monachino 39727, 39885 (mombin).*

*Wiley, J.R. 547 (radlkoferi).*

*Yuncker, T.G. 4623, 17086 (mombin); 18612 (purpurea).*

*Yuncker, T.G. et al. 8208 (mombin); 8592 (radlkoferi); 8650 (purpurea).*

*Zak, V. & Espinoza 4509 (mombin × globosa).*

*Zamora, N. & Morales 2251 (radlkoferi).*

*Zanoni, T. 15549 (dulcis).*

*Zanoni, T. & Jiménez 44521 (x robe [mombin x purpurea])*

*Zanoni, T. & Mejía 16269 (mombin); 16387 (dulcis).*

*Zanoni, T. et al. 14834 (mombin); 16840 (purpurea); 30298, 38991 (mombin).*

*Zarucchi, J.L. 4965 (mombin).*

*Zarucchi, J.L. & Barbosa 3552 (mombin).*

*Zarucchi, J.L. & Echeverry 4665 (purpurea).*

*Zarucchi, J.L. et al. 4935 (mombin).*

*Zehntner, L. 557, 558 (tuberosa).*
